# Shoe–ground loading responses across three 3D-printed arch-support heights in normal- and high-arched marathon runners: an arch-type-stratified secondary analysis

**DOI:** 10.3389/fspor.2026.1942713

**Published:** 2026-09-24

**Authors:** Xinran Dong, Tianzhe Liang, Yong Ma, Chengliang Wu

**Affiliations:** 1 Key Laboratory of Sports Engineering of General Administration of Sports of China, Wuhan Sports University, Wuhan, China; 2 School of Intelligent Sports Engineering, Wuhan Sports University, Wuhan, China; 3 Engineering Research Center of Sports Health Intelligent Equipment of Hubei Province, Wuhan Sports University, Wuhan, China; 4 Hubei Key Laboratory of Exercise Training and Monitoring, Wuhan Sports University, Wuhan, China

**Keywords:** foot arch morphology, marathon runners, midfoot force impulse, plantar pressure, running biomechanics

## Abstract

**Objective:**

A prior parameter-optimization study identified 9–15-mm support heights at 22%–26% infill as a candidate range for 3D-printed arch-support inserts, but whether responses within this range differ between runners with normal and high arches remains unclear. This arch-type-stratified secondary analysis compared shoe–ground interface loading under Single and 9-, 12-, and 15-mm support conditions at 24% infill and explored the temporal localization of condition-related differences during stance.

**Methods:**

The primary balanced analysis included thirty male marathon runners (15 normal arch, 15 high arch), and a sensitivity analysis included all 55 eligible runners (40 normal arch, 15 high arch). A floor-mounted Footscan® system recorded shoe–ground pressure and regional force data. Primary outcomes were maximum regional peak pressure (MaxPP), maximum metatarsal-region peak pressure (MetaPP), midfoot force impulse fraction (MIF), heel force impulse fraction (HIF), and mediolateral impulse index (MLI). Prespecified comparisons used paired or Welch *t*-tests with Benjamini–Hochberg false-discovery-rate correction, complemented by Arch Type × Condition linear mixed-effects models and exploratory pointwise time-series analyses.

**Results:**

Under Single, none of the five primary outcomes differed between arch groups after FDR correction. In the primary balanced analysis, relative to Single, FDR-supported differences were concentrated in impulse-distribution measures: MIF increased by 17.9%–19.5% in normal-arch runners under 12–24 and 15–24 and by 18.0%–23.0% in high-arch runners across all three supports, whereas MLI decreased by 14.0%–24.0% and 12.0%–19.7%, respectively. In this analysis, no MaxPP or MetaPP comparison met the FDR threshold. In normal arches, MIF was 7.4% higher under 12–24 than 9–24 (*q* = 0.041) and 9.0% higher under 15–24 than 9–24 (*q* = 0.003); no direct support-condition comparison was FDR-significant in high arches. Neither the 15 between-arch change comparisons nor the five Arch Type × Condition interactions reached FDR significance in the balanced analysis, and the same 0/15 and 0/5 pattern was retained in the full-cohort sensitivity analysis. Exploratory temporal differences were localized mainly to early-to-middle stance for midfoot force share and middle-to-late stance for dynamic MLI.

**Conclusion:**

In the primary balanced analysis, condition-related differences were expressed more consistently in MIF and MLI than in peak-pressure measures. Across the balanced and full-cohort analyses, there was insufficient evidence that static arch type systematically modified the height-specific or overall condition-related response pattern. The analyses did not establish overall superiority of either the 12- or 15-mm configuration. Future studies should integrate dynamic arch characteristics, comfort, and longer-term adaptation.

## 1 Introduction

During running, the foot repeatedly transmits impact and propulsive loads between the body and the ground, and the distribution of plantar loading provides useful information for characterizing foot–ground interaction during locomotion. Advances in plantar-pressure measurement and wearable sensing have expanded the assessment of loading patterns during dynamic activities (1, 2). Footwear structures, foot orthoses, and localized support elements are therefore widely studied as approaches for modifying regional loading during shod locomotion (3).

Insoles, foot orthoses, and localized pads provide defined support locations and allow structural features to be varied systematically. Previous studies have shown that forefoot pads, metatarsal pads, and related orthotic components can alter regional pressure patterns, with the observed responses depending on support location, configuration, and the locomotor task (3–6). Parametric investigations have further demonstrated dose-related differences in center-of-pressure and regional pressure patterns associated with forefoot and arch-support components (5, 7). Material properties also contribute to plantar-pressure responses to foot orthoses (8). Together, these findings indicate that arch-support configuration represents a multidimensional design problem and that its biomechanical characterization should extend beyond pressure reduction within a single region.

Foot-arch morphology is particularly relevant when evaluating arch-support configurations. Individuals with high or cavus arches generally exhibit less midfoot contact than those with normal arches and may show different distributions of plantar pressure across the forefoot, rearfoot, and lateral plantar regions (9, 10). Studies in runners with subtle cavus feet have also shown differences in plantar-pressure distribution when personalized orthotic insoles are used (11). However, previous work has largely examined specific foot types or orthotic parameters without directly determining whether condition-related loading patterns differ between runners stratified by normal and high arch morphology. Consequently, it remains unclear whether a candidate support-height range identified predominantly from normal-arch runners is associated with comparable loading patterns in runners with high arches.

A previous parameter-optimization study examined 24 combinations of six support heights and four infill densities and identified support heights of 9–15 mm and infill densities of 22%–26% as a candidate parameter range for further investigation (12). The data analyzed in the present study were drawn from the same original experimental campaign. The present study therefore constitutes an arch-type-stratified secondary analysis of that experiment. It focuses on the previously unreported high-arch subgroup and uses an outcome-blind random subset of the previously analyzed normal-arch cohort as a balanced comparator. For the present analysis, the Single, H9/D24, H12/D24, and H15/D24 trials were extracted from the original experimental dataset. This analysis extends the previous parameter-optimization work by examining the selected candidate conditions in runners stratified by normal and high arch morphology.

Characterization of the resulting loading patterns should not rely on peak-pressure measures alone. Prior work has emphasized that peak and time-integrated metrics quantify loading through different mathematical constructs, although substantial interdependence between them has also been reported (13–15). In the present analysis, regional force-impulse measures were therefore considered alongside peak-pressure measures. The five primary outcomes were maximum regional peak pressure (MaxPP), maximum metatarsal-region peak pressure (MetaPP), midfoot force impulse fraction (MIF), heel force impulse fraction (HIF), and mediolateral impulse index (MLI). Because the dynamic data were recorded using a floor-mounted Footscan® pressure plate, these outcomes are interpreted specifically as shoe–ground interface loading measures. Exploratory time-series analyses of instantaneous midfoot and heel force shares and dynamic MLI were additionally used to localize condition-related differences across the stance phase.

In summary, the present arch-type-stratified secondary analysis aimed to characterize shoe–ground interface loading patterns across the Single, H9/D24, H12/D24, and H15/D24 conditions in male marathon runners with normal and high arches. The analysis addressed three principal questions. First, did the normal- and high-arch groups differ in the five primary loading outcomes under the Single condition? Second, within each arch group, what condition-related differences were observed relative to Single, and were there direct differences among the three selected support conditions? Third, did the magnitude of participant-level changes or the overall pattern of condition-related differences vary between the normal- and high-arch groups? In addition, exploratory time-series analyses were used to localize differences in instantaneous midfoot and heel force shares and dynamic MLI across the stance phase.

## 2 Materials and methods

### 2.1 Subjects and ethical approval

This study was conducted in accordance with the Declaration of Helsinki, and all experimental procedures were approved by the Medical Ethics Committee of Wuhan Sports University (approval no. 2025124). Before testing, all participants received detailed information regarding the experimental procedures and potential risks and provided written informed consent.

The original experimental campaign recruited 69 male runners with experience completing a full marathon. Recruitment criteria included male sex; self-reported habitual shoe size 42, used to improve compatibility with the standardized study footwear; experience completing a full marathon within 2.5–4.5 h (12, 16); no lower-limb musculoskeletal injury within the preceding 6 months (17); and the ability to perform the running protocol using the required rearfoot strike pattern (18). Participant-level screening exclusions comprised flatfoot or inability to obtain a clear normal- or high-arch classification and habitual forefoot striking that was incompatible with the intended running protocol (18). Protocol non-completion after testing had begun was recorded separately from screening exclusion. Trial-level technical invalidity was handled during data processing as described in Section 2.5.

Fourteen runners were not retained for the complete-protocol sampling frame. One participant was excluded during arch screening because of flatfoot, and four were excluded because habitual forefoot striking did not meet the intended running-protocol requirement. Nine participants did not complete the full experimental protocol after testing had begun. Four discontinued after completing part of the protocol because personal scheduling constraints prevented completion of the full testing session. Five participants, despite having self-reported habitual shoe size 42 at recruitment, were found during testing to have inadequate fit in the standardized EUR size 42 study shoe and therefore did not complete the full protocol. No protocol discontinuation was documented as fatigue-related or as being caused by an adverse response attributable to a specific arch-support configuration.

The participants and experimental trials analyzed in the present study were drawn from the same original experimental campaign as our previous parameter-optimization study (12). That previous study focused on runners with normal arches, whereas the high-arch subgroup from the same experimental campaign was not included in the previous analysis. The present study therefore represents an arch-type-stratified secondary analysis that extends our previous work by incorporating the previously unreported high-arch subgroup and a balanced normal-arch comparator. No new participant testing or repeat experimental trials were conducted for the present analysis.

All 15 eligible high-arch runners were retained. For the balanced normal-arch comparator, a sampling file containing only the unique study identifier and arch classification was prepared before the primary loading outcomes were accessed. A computer-generated random sequence was used to order the 40 eligible normal-arch study identifiers, and the first 15 identifiers were selected without replacement. The selected list was fixed before primary-outcome analysis. The final balanced analytic sample therefore comprised 30 runners, with 15 participants in each arch group. The sampling procedure and baseline age, height, and body-mass characteristics are reported in Supplementary Methods S10 and Table S10.

### 2.2 Foot arch classification

During the original experimental campaign, foot-arch classification was completed before the running trials and before construction of the balanced analytic sample using a two-stage static assessment. In the first stage, bilateral weight-bearing two-dimensional footprints were obtained using the DigiPED plantar scanning system, with participants standing naturally and distributing body weight across both feet (10). The three-line method was used as an initial morphological screening procedure and was not treated as the sole criterion for final arch classification.

In the second stage, participants underwent a separate barefoot static standing assessment on the Footscan® pressure platform. The resulting static footprints were used to characterize midfoot-contact morphology and, when measurable, to derive quantitative supporting descriptors including arch angle (AA) and the Chippaux–Smirak index (CSI) (19, 20). AA and CSI were used to support the morphological assessment rather than as independent mandatory cutoff criteria. Final participant-level arch classification was based on concordant findings from the two-stage assessment. Each foot was classified separately: a high arch required a high-arch DigiPED pattern together with a concordant Footscan® footprint showing markedly reduced or absent midfoot contact, whereas a normal arch required a non-high-arch DigiPED pattern with continuous and moderate midfoot contact. Participants were classified as normal arch only when both feet were normal; if one foot was classified as high arch and the contralateral foot as normal, the participant was assigned to the high-arch group. Indeterminate cases were not assigned to either group. Because arch characteristics may differ between sides (21), bilateral information was retained in participant-level classification. This grouping reflects static foot morphology rather than dynamic arch deformation or stiffness.

No fixed numerical cutoff for AA or CSI was prespecified for participant classification. The available distributions of these quantitative supporting descriptors in the complete eligible cohort and the balanced analytic sample are summarized in Supplementary Table S10, Panel C. Arch classification was completed before the running trials; therefore, the primary dynamic shoe–ground loading outcomes analyzed in the present study had not yet been generated at the time of classification and could not have informed the classification decision. The archived documentation available for this secondary analysis did not allow the identity of the original individual assessor to be verified; therefore, the classification is not retrospectively attributed to a specific assessor. Formal intra- or inter-rater reliability of the composite classification procedure was not assessed during the original experimental campaign.

### 2.3 Experimental equipment and support conditions

Dynamic shoe–ground interface pressure and regional force data during shod running were recorded using a Footscan® floor-mounted pressure plate system (RSscan International; spatial resolution, 5.08 mm × 7.62 mm; sampling frequency, 200 Hz). The platform was calibrated before testing according to the manufacturer's recommended procedure. Because the system was floor mounted rather than positioned inside the shoe, the recorded signals represent loading at the shoe–ground interface and do not directly measure pressure at the plantar-skin–insole interface.

The standardized running shoe used during the original experimental campaign was the Li-Ning Yueying 4 (EUR size 42). The Single reference condition consisted of the standardized shoe with its original removable insole and without an additional arch-support insert. The H9/D24, H12/D24, and H15/D24 configurations analyzed in the present study were selected from the insert designs evaluated in our previous parameter-optimization study (12). That study evaluated six nominal support heights (3, 6, 9, 12, 15, and 18 mm) across four infill densities (20%, 22%, 24%, and 26%) and identified 9–15 mm and 22%–26% as a candidate parameter range for further investigation (12). The present secondary analysis extracted the existing H9/D24, H12/D24, and H15/D24 trials rather than introducing newly designed or newly tested conditions.

The three selected height models shared the same projected footprint dimensions, with a projected longitudinal length of approximately 129.85 mm and a maximum projected width of 58.22 mm. Their nominal support heights were 9, 12, and 15 mm for the H9/D24, H12/D24, and H15/D24 configurations, respectively. Thus, the three height configurations retained the same projected length and width while differing in nominal support height. The principal geometric dimensions are summarized in Supplementary Table S14. The corresponding three-dimensional surface geometries are provided as Supplementary CAD Files S1–S3 within the accompanying archive Supplementary_CAD_Files_S1–S3.zip; individual STL filenames and condition mappings are provided in Supplementary Table S14. For each support height, the left- and right-foot inserts used experimentally were mirror-image counterparts. With the tapered end oriented anteriorly, the more strongly curved side was oriented laterally and the relatively flatter side medially for the corresponding foot. The contralateral insert used experimentally had the corresponding mirror-image geometry.

All inserts were manufactured from thermoplastic polyurethane (TPU) using fused deposition modeling on a Raise3D Pro3 printer. During printing, the planar base was positioned on the build plate and the convex arch-support surface faced upward. The printing parameters were a 0.2-mm layer height, a 0.8-mm nozzle diameter, a 220 °C nozzle temperature, a printing speed of 40 mm/s, and a gyroid infill pattern. Infill density was fixed at 24% for all three configurations. The 24% infill configuration had a measured effective structural hardness of Shore C 38–41 in our previous study (12). Printed inserts were conditioned for 24 h at 23 ± 2 °C and 50 ± 5% relative humidity before testing.

During testing, each insert was positioned beneath the original removable insole of the standardized running shoe, aligned with the medial longitudinal arch, and secured using transparent acrylic double-sided adhesive tape (0.2 mm thickness, 20 mm width). The original insole was then replaced over the insert. For a given foot side, the three analyzed height conditions shared the same projected footprint dimensions, material, infill specification, manufacturing settings, placement, and fixation procedure, while differing in nominal support height.

### 2.4 Testing protocol

All trials analyzed in the present study were collected during the original multi-condition experimental campaign underlying our previous parameter-optimization study (12). The present secondary analysis did not involve additional participant testing, retesting, or a new randomization procedure; the existing Single, H9/D24, H12/D24, and H15/D24 trials were extracted from the original dataset. In the original experiment, the insert conditions were administered using a restricted, semi-randomized middle-out sequence rather than complete randomization. Testing prioritized the middle-gradient range, beginning with a randomly selected condition from the 9–12-mm support-height range and subsequently expanding in randomized order toward higher or lower support levels (12). The acquisition order of the extracted trials was therefore inherited unchanged from the original experimental campaign. The experimental procedure is shown in Figure 1.

**Figure 1:**
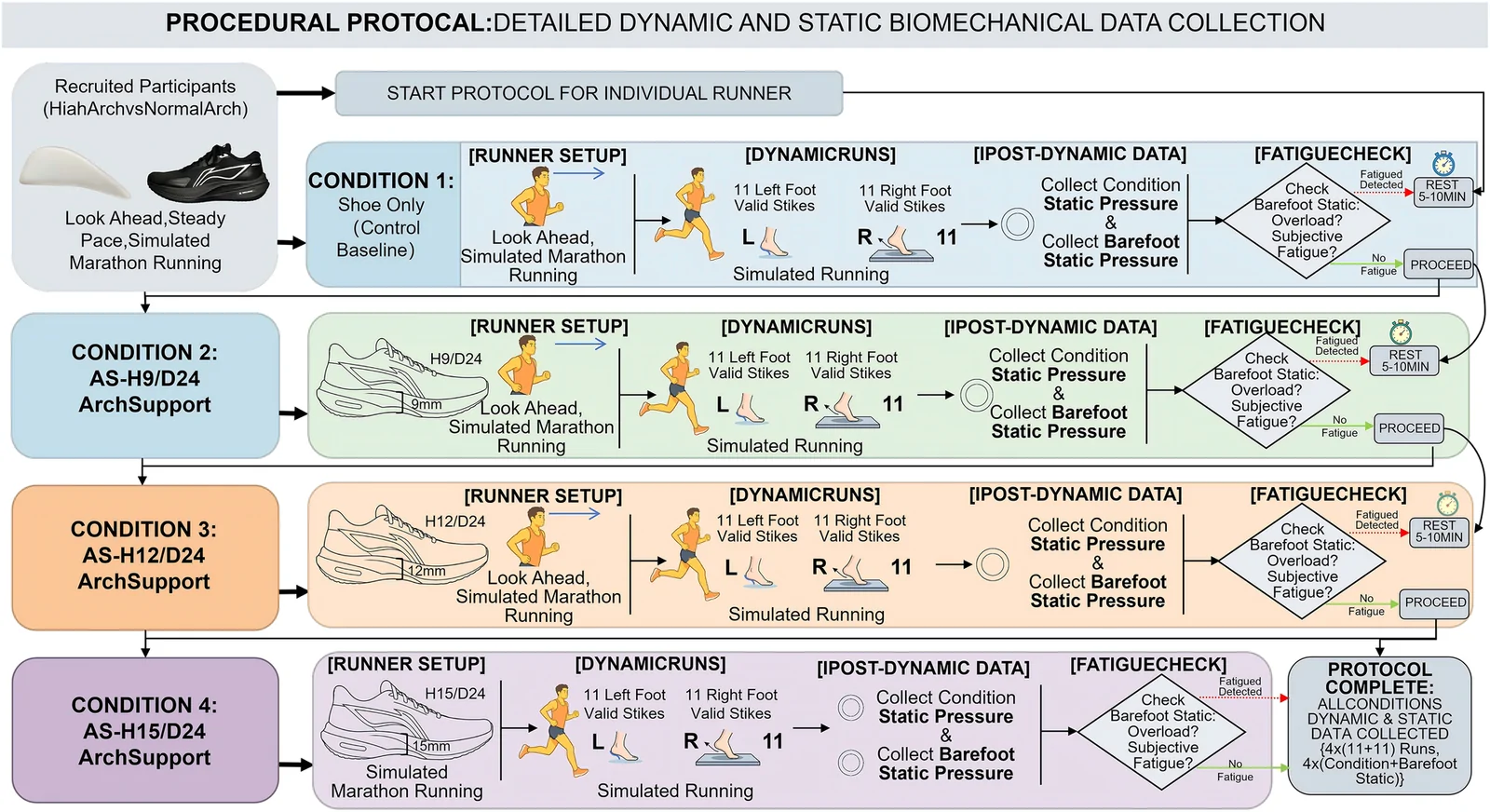
Overview of the original experimental protocol and the conditions extracted for the present secondary analysis.

The original experiment comprised a multi-condition testing campaign using a restricted, semi-randomized middle-out sequence. The present analysis extracted the existing Single, H9/D24, H12/D24, and H15/D24 trials. Dynamic shoe–ground interface recordings were accompanied by auxiliary shod and barefoot static assessments and procedural subjective monitoring.

Participant-specific target running pace was established from each runner's documented marathon performance and habitual running pace. Participants were instructed to run at their usual marathon-style pace, which was monitored during testing using a sports watch. Trials outside ±5% of the participant-specific target pace were not retained. No timing gates or other independent measurements of instantaneous approach speed at pressure-plate contact were available in the original experimental protocol (12).

Dynamic testing followed a mid-gait protocol. Participants were instructed to look straight ahead and run naturally across the pressure-plate area without deliberately targeting the plate (22). During the preliminary testing, each participant's starting position was adjusted to facilitate natural and complete shoe contact within the effective sensing area. A trial was considered technically invalid if the participant exhibited an obvious change in running direction or stride pattern, deliberately targeted the pressure plate, failed to achieve complete shoe contact within the sensing area, did not trigger the recording system, or produced an incomplete signal. Under each analyzed condition, participants completed 11 shuttle runs, from which at least three valid and stable dynamic recordings were obtained for each foot.

After completion of the dynamic trials under each analyzed condition, two static pressure assessments were obtained at different measurement interfaces. First, shod shoe–ground interface pressure was recorded while participants wore the standardized running shoes with the insert assigned to the corresponding condition. Second, barefoot skin–ground interface pressure was recorded after the shoes were removed (23, 24). These static measurements were retained as auxiliary descriptors of post-condition contact patterns and foot–footwear interaction rather than as part of the five-outcome primary inferential analysis (23). Importantly, neither static assessment represents the plantar-skin–insole interface during dynamic running.

At the end of each experimental condition, subjective comfort, perceived support, fatigue, and pain were recorded as procedural monitoring measures to characterize immediate tolerability and readiness to continue testing. Overall comfort was assessed using the original 5-point scale, perceived support intensity using the 15-point scale, and fatigue and pain using 100-mm visual analogue scales (VASs), with higher VAS values indicating greater fatigue or pain (12). Testing was terminated if the pain VAS reached 50 mm or higher, in accordance with the original protocol (12). These subjective measures were not primary biomechanical outcomes and were not used to rank the three selected support heights in the present analysis. Descriptive subjective data for the analyzed support conditions are reported in Supplementary Table S15.

### 2.5 Data processing, outcome derivation, and quality control

The validity of each dynamic trial was determined from the testing records and the completeness of the shoe–ground contact footprint. A trial was excluded if the recording system was not triggered, the participant made an obvious change in direction or stride pattern, deliberately targeted the pressure plate, failed to place the entire shoe sole within the sensing area, or produced an incomplete signal. Retained trials were entered into the consolidated pressure-force dataset. A custom Python pipeline standardized participant identity, arch type, condition, foot side, and trial number and verified data completeness and uniqueness of the expected observational units. Numerical quality-control checks were subsequently applied to the retained primary pressure–force trials. Potential statistical outliers were flagged for audit but were not automatically removed. Detailed procedures are reported in Supplementary Methods S2, S3 and Table S7.

The consolidated pressure-force dataset was the sole inferential source for the five primary discrete outcomes. Original Footscan® regional force-time exports were analyzed separately for exploratory temporal analyses and were not appended to the consolidated primary trial table or used to replace missing primary statistical units. Center-of-pressure, gait-timing, regional contact-area, and static-pressure data were treated as auxiliary data streams. Subjective ratings collected during the experimental protocol were treated as procedural or descriptive monitoring measures and did not enter the five-outcome primary inferential analysis. The complete data-source hierarchy and analytical roles are provided in Supplementary Table S1.

Regional shoe–ground peak pressure and regional force impulse were obtained for 10 Footscan-defined functional regions: hallux (T1), second-to-fifth toes (T2–5), first-to-fifth metatarsals (M1–M5), midfoot (MF), medial heel (HM), and lateral heel (HL). These region labels were treated as functional Footscan partitions of the recorded shoe–ground interface and were not interpreted as direct measurements of loading beneath the corresponding plantar tissues.

The five primary discrete outcomes were maximum regional peak pressure (MaxPP), maximum metatarsal-region peak pressure (MetaPP), midfoot force impulse fraction (MIF), heel force impulse fraction (HIF), and mediolateral impulse index (MLI). MaxPP was defined as the maximum regional peak pressure across the 10 functional regions, whereas MetaPP was defined as the maximum across M1–M5. MIF and HIF represented the proportions of total regional force impulse contributed by the midfoot and combined heel regions, respectively. MLI represented the signed relative contribution of the predefined medial and lateral impulse sets. Exact formulas, units, region definitions (7, 9, 13, 19), and interpretation boundaries are provided in Supplementary Tables S2, S3.

Primary discrete outcomes were first calculated at the individual-trial level. Within each participant, condition, and foot, the three retained trials were averaged. Left- and right-foot means were then averaged with equal weight to yield one participant-condition observation. This aggregation prevented repeated trials and the two feet from being treated as independent statistical units (25).

For each selected support condition, participant-level absolute change relative to Single was defined as shown in Equation 1:

$$\Delta_{i,c}=X_{i,c}-X_{i,Single}$$ (1)

Here, *i* denotes the participant and *c* denotes the AS-H9/D24, AS-H12/D24, or AS-H15/D24 condition. Group-level percentage differences displayed in the principal figures were calculated from complete-pair group means as shown in Equation 2:

$$100\times \frac{{\bar{X}}_{A}-{\bar{X}}_{B}}{{\bar{X}}_{B}}$$ (2)

Here, *A* denotes the target condition and *B* the corresponding reference condition. These quantities were calculated from group means rather than as the mean of participant-level percentage ratios.

Exploratory time-series analyses were conducted separately using the original regional force-time exports. The main exploratory frame used all successfully parsed valid trials available for each foot. Each trial curve was normalized to 0%–100% stance and resampled to 101 points, after which trials were averaged within foot and the left- and right-foot curves were averaged with equal weight to obtain participant-condition-time curves.

The temporal metrics were instantaneous midfoot force share [MFS(t)], instantaneous heel force share [HFS(t)], and dynamic MLI(t). These instantaneous force-composition variables are distinct from stance-integrated MIF and HIF. At each normalized time point, the force-share variables were calculated only when the total recorded force was positive and finite. No additional minimum total-force threshold was imposed. To reduce instability of ratio-based variables during low-force portions near initial contact and toe-off, pointwise inferential testing was restricted to 5%–95% stance.

A separate first-three-valid-trials-per-foot frame was analyzed as a sampling-frame sensitivity analysis. Full procedures and sensitivity results are provided in Supplementary Methods S6 and Tables S4, S11, S12.

An exploratory candidate score was retained only as a descriptive multimetric summary and was not treated as a primary outcome, subjected to formal hypothesis testing, or interpreted as statistical evidence that one support configuration was superior to another. Its calculation is described in Supplementary Methods S8.

### 2.6 Statistical analysis

Statistical analyses of the five primary discrete outcomes were conducted at the participant-condition level. Continuous data are reported as the mean ± standard deviation together with mean differences, 95% confidence intervals, and standardized effect sizes (26). Differences in MaxPP and MetaPP are expressed in N/cm^2^, differences in MIF, HIF, and MLI are expressed in percentage points. All hypothesis tests were two-sided.

Under Single, arch groups were compared using Welch's independent-samples *t* test, with the difference defined as High minus Normal and Hedges' *g* reported (27). Within each arch group, paired-samples t tests compared AS-H9/D24 (9–24), AS-H12/D24 (12–24), and AS-H15/D24 (15–24) separately with Single; differences were defined as support condition minus Single and Cohen's *d_z_* was reported. Because Single preceded the insert conditions, these contrasts were treated as condition-related differences from the initial reference rather than isolated causal effects.

The three selected support configurations were compared directly within each arch group using paired-samples *t* tests. Prespecified directions were 12–24 minus 9–24, 15–24 minus 9–24, and 15–24 minus 12–24, with Cohen's *d_z_* reported. For each support condition, participant-level change was defined as Δ = support condition minus Single. Δ was compared between arch groups using Welch's independent-samples *t* tests; differences were defined as High Δ minus Normal Δ and Hedges' *g* was reported. These 15 tests directly evaluated height-specific between-arch differences in condition-related change (28).

As a complementary omnibus analysis, a linear mixed-effects model was fitted separately for each primary outcome using 120 participant-condition observations (30 participants × 4 conditions): Outcome ∼ Arch Type + Condition + Arch Type × Condition + (1 | Participant). Arch Type, Condition, and their interaction were fixed effects, and participant was a random intercept. Models were fitted by maximum likelihood. The omnibus interaction was tested by likelihood-ratio comparison of the full model with a reduced model omitting Arch Type × Condition; the test had 3 degrees of freedom. A conditional Monte Carlo design-sensitivity analysis (200,000 replicates; 80% target power) quantified the minimum detectable omnibus interaction under three declared interaction-shape scenarios using the outcome-specific residual variances from the fitted models; full assumptions and results are reported in Supplementary Methods S7 and Table S20.

Benjamini–Hochberg FDR correction was applied separately to five prespecified families: five Single baseline comparisons (29), 30 support-vs.-Single comparisons, 30 direct configuration contrasts, 15 between-arch Δ comparisons, and five omnibus interaction tests. Multiplicity-controlled inference was based on q values, with exact unadjusted *p* values also reported. The families and permissible inferences are summarized in Supplementary Table S6; complete numerical results are provided in Supplementary Tables S13 and S17–S19.

To assess sensitivity to the balanced sampling of the normal-arch group, all 55 eligible runners (40 normal arch and 15 high arch; 220 participant-condition observations) were additionally analyzed. The same primary outcomes, statistical unit, discrete comparison framework, and mixed-effects model specification were retained. The balanced 15:15 sample remained the primary analytic sample, whereas the 40:15 analysis was treated as a full-cohort sensitivity analysis. Model-based interaction contrasts were reported with 95% Wald confidence intervals, and complete sensitivity results are provided in Supplementary Results S12 and Tables S21–S30.

Distributional assumptions were evaluated using Shapiro–Wilk tests of paired-difference distributions for paired analyses and group-specific distributions for independent analyses. Wilcoxon signed-rank and Mann–Whitney *U* tests were used as rank-based robustness analyses. Interquartile-range and modified-Z criteria flagged observations for influence auditing but did not determine trial inclusion.

The visually distinct S07 MIF response was evaluated by re-auditing the retained raw trials, conducting rank-based analyses with S07 retained, and repeating relevant analyses after leaving S07 out. The full high-arch sample (*n* = 15) remained the primary analysis; the audit and influence results are reported in Supplementary Table S16.

For exploratory time-series analyses, participant-level support-minus-Single differences were tested against zero at 91 points from 5% to 95% stance, separately for each arch group, support condition, and temporal metric (18 curves). One-sample t tests were used, and BH-FDR correction was applied separately within each 91-test curve. Contiguous *q* < 0.05 points were summarized as temporal sampling-point runs, not as SPM clusters, whole-phase effects, or whole-curve effects.

The complete pointwise procedure was repeated using the first three successfully parsed valid trials in each participant × condition × foot cell. The all-valid-trial frame was the primary temporal analysis, and the first-three frame evaluated sensitivity to trial sampling. Results are reported in Supplementary Tables S11 and S12 and Supplementary Figure S1.

## 3 Results

### 3.1 Baseline biomechanical characteristics of runners with normal and high arches

Complete participant-level data were available for 15 normal-arch and 15 high-arch runners. Under Single, none of the five between-arch comparisons met the BH-FDR threshold (all *q* ≥ 0.101; Figure 2; Table 1; Supplementary Table S17, Panel A).

**Figure 2:**
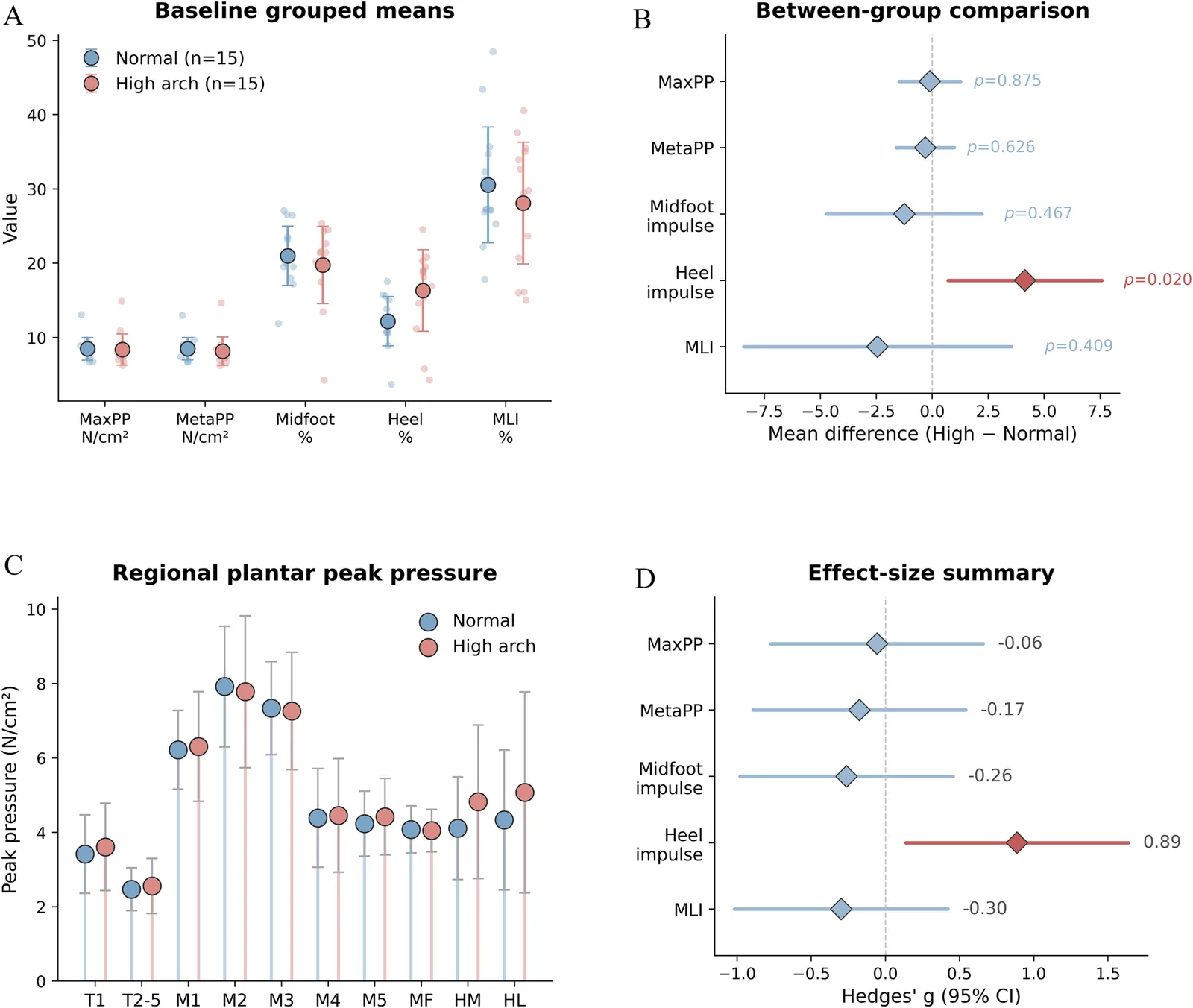
Baseline shoe–ground loading characteristics under Single. **(A)** Participant values and group means ± standard deviations for the five primary outcomes. **(B)** Between-arch mean differences (High minus Normal) with 95% confidence intervals. **(C)** Descriptive regional shoe–ground peak pressures for the ten Footscan® functional regions. **(D)** Hedges’ *g* for the five primary outcomes. T1, hallux; T2–5, second-to-fifth toes; M1–M5, metatarsal regions; MF, midfoot; HM, medial heel; HL, lateral heel; MIF, midfoot force impulse fraction; HIF, heel force impulse fraction; MLI, mediolateral impulse index.

**Table 1:** Primary outcome summary.

Panel A. Group-by-condition descriptive statistics						
Arch type	Condition	MaxPP (N/cm^2^)	MetaPP (N/cm^2^)	MIF (%)	HIF (%)	MLI (%)
Normal	Single	8.48 ± 1.53	8.48 ± 1.51	21.00 ± 3.98	12.19 ± 3.32	30.54 ± 7.79
Normal	9–24	8.20 ± 0.71	8.20 ± 0.71	23.04 ± 2.80	10.44 ± 3.10	26.28 ± 6.57
Normal	12–24	7.76 ± 0.83	7.76 ± 0.83	24.75 ± 3.17	10.37 ± 3.71	24.40 ± 8.21
Normal	15–24	7.74 ± 0.79	7.72 ± 0.81	25.10 ± 2.36	11.06 ± 2.78	23.19 ± 5.34
High	Single	8.38 ± 2.10	8.17 ± 1.93	19.75 ± 5.20	16.33 ± 5.50	28.09 ± 8.19
High	9–24	7.82 ± 0.81	7.81 ± 0.82	23.31 ± 2.16	13.71 ± 4.28	24.71 ± 8.80
High	12–24	7.80 ± 0.80	7.76 ± 0.84	23.96 ± 2.55	13.79 ± 4.51	22.68 ± 7.78
High	15–24	7.59 ± 0.90	7.55 ± 0.94	24.29 ± 2.93	14.15 ± 4.39	22.56 ± 7.47

Panel B. Multiplicity-controlled inferential summary		
Analysis family	Multiplicity-controlled result	Complete numerical results
Single baseline (5 tests)	0/5 FDR significant; HIF *p* = 0.020, *q* = 0.101; all other *q* ≥ 0.779.	Supplementary Table S17, Panel A
Support vs. Single (30 tests)	13/30 FDR significant: Normal—MIF (12–24, 15–24), MLI (all), HIF (9–24); High—MIF (all), MLI (all), HIF (9–24).	Supplementary Table S17, Panel B
Configuration contrasts (30 tests)	2/30 FDR significant, both Normal MIF: 12–24 − 9–24, +1.71 pp [0.70, 2.73], *p* = 0.003, *q* = 0.041, *d_z_* = 0.94; 15–24 − 9–24, +2.07 pp [1.24, 2.89], *p* < 0.001, *q* = 0.003, *d_z_* = 1.39.	Supplementary Table S18
Between-arch Δ (15 tests)	0/15 FDR significant; all *p* ≥ 0.298; all *q* = 0.934.	Supplementary Table S19
Arch Type × Condition LMM (5 tests)	0/5 omnibus interactions FDR significant; *χ^2^* (3) = 0.859–2.339; *p* = 0.505–0.835; all *q* = 0.835.	Supplementary Table S13

Panel A reports participant-level group means ± standard deviations for all four conditions. Panel B summarizes the five prespecified inferential families; full effect estimates, 95% confidence intervals, exact *p* values, BH-FDR *q* values, and standardized effect sizes are reported in the cited supplementary tables. Note. Δ = support condition − Single. Between-arch differences are High Δ − Normal Δ. pp, percentage points; *d_z_*, paired-sample Cohen's *d_z_*. Nonsignificant interaction and between-arch Δ tests do not establish equivalence.

MaxPP was 8.48 ± 1.53 N/cm^2^ in the normal-arch group and 8.38 ± 2.10 N/cm^2^ in the high-arch group (High minus Normal: −0.11 N/cm^2^, 95% CI −1.49 to 1.27; *p* = 0.875; *q* = 0.875; *Hedges' g* = −0.06). MetaPP was 8.48 ± 1.51 N/cm^2^ in the normal-arch group and 8.17 ± 1.93 N/cm^2^ in the high-arch group (−0.31 N/cm^2^, 95% CI −1.61 to 0.99; *p* = 0.626; *q* = 0.783; *g* = −0.17; Figures 2A,B). MIF was 21.00 ± 3.98% in the normal-arch group and 19.75 ± 5.20% in the high-arch group (−1.25 percentage points, 95% CI −4.72 to 2.23; *p* = 0.467; *q* = 0.779; *g* = −0.26). HIF was 12.19 ± 3.32% and 16.33 ± 5.50%, respectively (+4.14 percentage points, 95% CI 0.71–7.57; *p* = 0.020; *q* = 0.101; *g* = 0.89). MLI was 30.54 ± 7.79% and 28.09 ± 8.19%, respectively (−2.45 percentage points, 95% CI −8.43 to 3.53; *p* = 0.409; *q* = 0.779; *g* = −0.30; Figures 2A,B,D).

Descriptive regional shoe–ground peak pressures across the ten Footscan® functional regions are shown in Figure 2C; these regional descriptors were not included in the five-outcome baseline multiplicity family.

### 3.2 Within-group changes in shoe–ground loading across support conditions

Across the selected support conditions, group means for MaxPP and MetaPP were numerically lower, MIF was higher, and MLI was lower than under Single in both arch groups. Under 15–24, MaxPP/MetaPP were 7.74/7.72 N/cm^2^ in the normal-arch group and 7.59/7.55 N/cm^2^ in the high-arch group; MIF was 25.10% and 24.29%, MLI was 23.19% and 22.56%, and HIF was 11.06% and 14.15%, respectively (Figures 3A,B; Table 1A).

**Figure 3:**
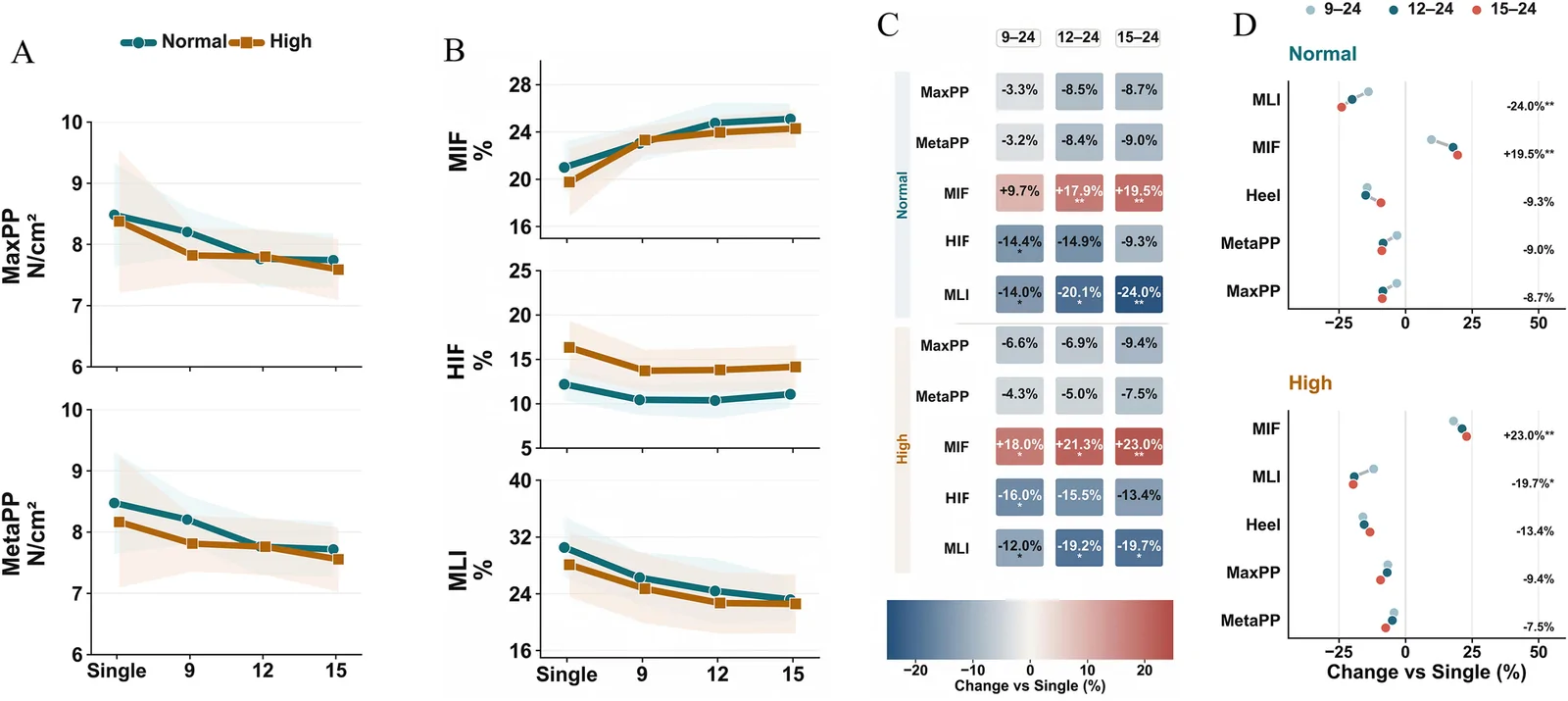
Within-group differences in shoe–ground loading relative to Single. **(A,B)** Group means and 95% confidence intervals across Single, 9–24, 12–24, and 15–24 for MaxPP/MetaPP and MIF/HIF/MLI, respectively. **(C)** Group-mean percentage differences, calculated as 100 × (support − Single)/Single; asterisks denote *q* < 0.05 within the 30-test support-vs.-Single family. **(D)** Direction-aligned percentage differences by condition; the annotated values are the 15–24 differences. Effect-size or percentage ordering is descriptive and does not constitute a formal comparison among outcomes.

In the normal-arch group, FDR-supported support-vs.-Single differences comprised higher MIF under 12–24 and 15–24 (+17.9%, *q* = 0.005; +19.5%, *q* = 0.005), lower MLI under 9–24, 12–24, and 15–24 (−14.0%, *q* = 0.015; −20.1%, *q* = 0.012; −24.0%, *q* = 0.005), and lower HIF under 9–24 (−14.4%, *q* = 0.020). In the high-arch group, MIF was higher and MLI lower under all three support conditions (MIF *q* = 0.020, 0.012, and 0.005; MLI *q* = 0.026, 0.015, and 0.015), and HIF was lower under 9–24 (*q* = 0.044). No MaxPP or MetaPP comparison met the FDR threshold (Figures 3C,D; Supplementary Table S17, Panel B).

### 3.3 Descriptive response pattern and robustness in high-arch runners

Within the high-arch group, support-vs.-Single comparisons showed the clearest FDR-supported condition-related differences for MIF and MLI. MIF was 18.0%, 21.3%, and 23.0% higher under the 9–24, 12–24, and 15–24 conditions, respectively (*q* = 0.020, 0.012, and 0.005), whereas MLI was 12.0%, 19.2%, and 19.7% lower, respectively (*q* = 0.026, 0.015, and 0.015; Figure 4A). HIF was numerically 16.0%, 15.5%, and 13.4% lower than under Single; however, only the 9–24 contrast met the FDR threshold (*q* = 0.044), whereas the 12–24 and 15–24 contrasts did not (*q* = 0.056 and 0.144, respectively). No MaxPP or MetaPP support-vs.-Single comparison met the FDR threshold (Figure 4A; Supplementary Table S17, Panel B). These support-vs.-Single comparisons describe condition-related differences and do not by themselves establish differences among the three support heights.

**Figure 4:**
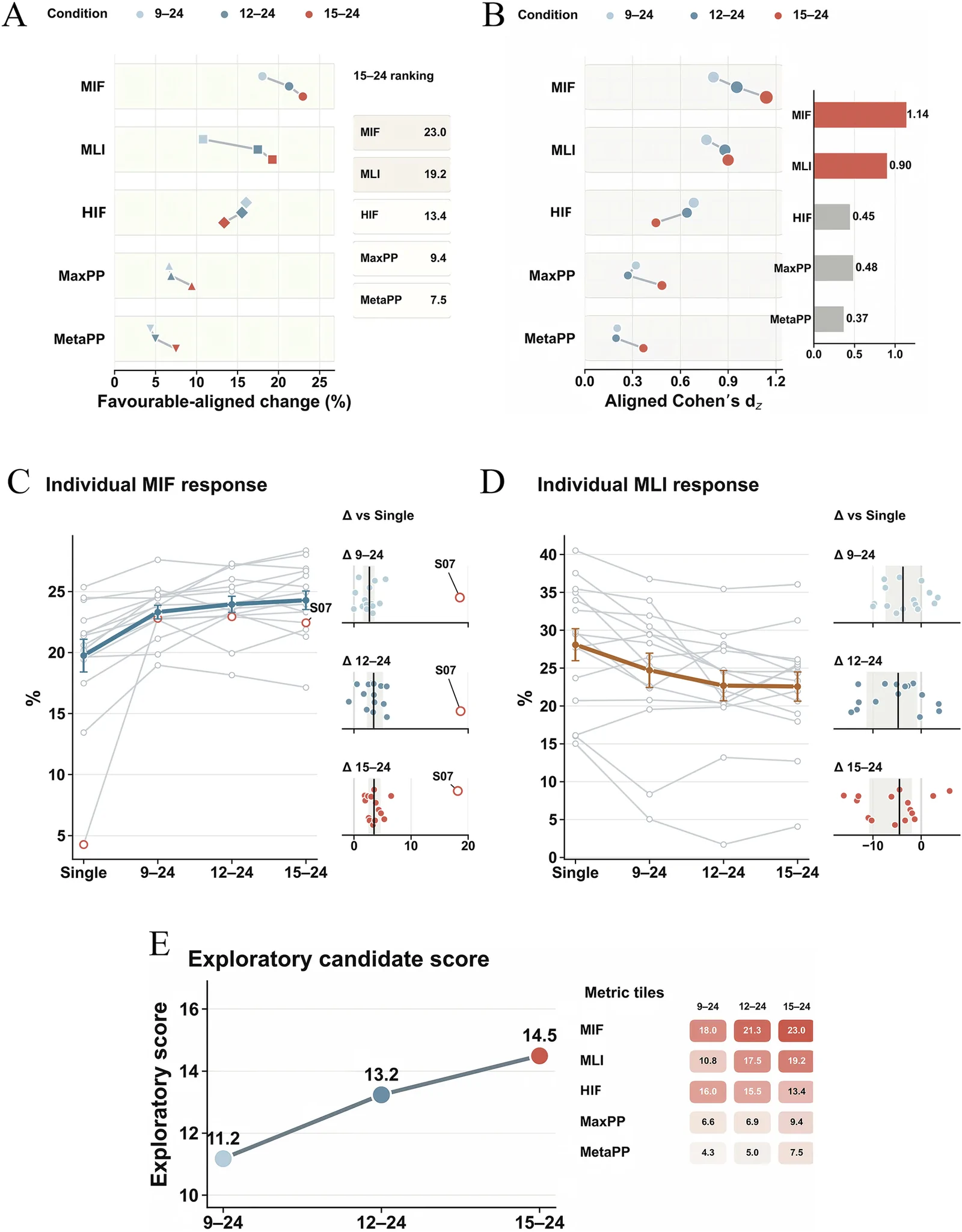
High-arch support-vs.-Single response summaries. **(A)** Direction-aligned group-mean percentage differences; asterisks indicate *q* < 0.05 in the 30-test support-vs.-Single family. **(B)** Direction-aligned paired Cohen's *d_z_* values; numerical ordering does not represent a significance ranking. **(C,D)** Participant trajectories and paired differences for MIF and MLI; S07 is identified in panel ***C***. **(E)** Exploratory descriptive candidate score and its five equally weighted components (MaxPP, MetaPP, MIF, HIF, and MLI). MIF was aligned in the increasing direction; the other components were aligned in the decreasing direction. The score was not hypothesis tested. Independent re-audit of S07's 24 selected raw trials reproduced all five primary values and the higher support-condition MIF across both feet and repeated trials, identified no export-level technical exclusion criterion, and supported retaining S07 (Supplementary Table S16).

Under the 15–24 condition, the absolute paired effect sizes were numerically larger for MIF (|*d_z_*| = 1.14) and MLI (|*d_z_*| = 0.90) than for MaxPP (|*d_z_*| = 0.48), HIF (|*d_z_*| = 0.45), and MetaPP (|*d_z_*| = 0.37; Figure 4B). This numerical ordering was descriptively consistent with the percentage-difference pattern; however, no formal statistical comparison of effect sizes across outcomes was performed.

Descriptively, the group mean of MIF increased from 19.75% under Single to 23.31%, 23.96%, and 24.29% under 9–24, 12–24, and 15–24, respectively, whereas the group mean of MLI decreased from 28.09% under Single to 24.71%, 22.68%, and 22.56% (Figures 4C,D). These numerical trends should not be interpreted as evidence of a monotonic height-dependent response because no direct support-condition contrast reached the FDR threshold in the high-arch group.

The exploratory descriptive candidate score increased numerically from 11.2 under 9–24 to 13.2 under 12–24 and 14.5 under 15–24 (Figure 4E). Inspection of its equally weighted components indicated that this numerical increase was driven mainly by the MIF and direction-aligned MLI components, whereas the MaxPP, MetaPP, and HIF components showed smaller variation across conditions. The candidate score was not subjected to hypothesis testing and should therefore not be interpreted as evidence that one support height was statistically superior to another.

The targeted audit of participant S07 reproduced the five primary outcome values from the retained raw trials. The principal MIF and MLI patterns were also retained in the rank-based and leave-S07-out robustness analyses, whereas the HIF inference was more sensitive to the analytical frame (Supplementary Table S16). Accordingly, S07 was retained in the primary high-arch analysis.

### 3.4 Comparisons among different support heights

Pairwise comparisons among the three support configurations identified FDR-corrected differences only for MIF in the normal-arch group. In contrast, no pairwise comparison among support heights reached FDR-corrected significance in the high-arch group (Figure 5).

**Figure 5:**
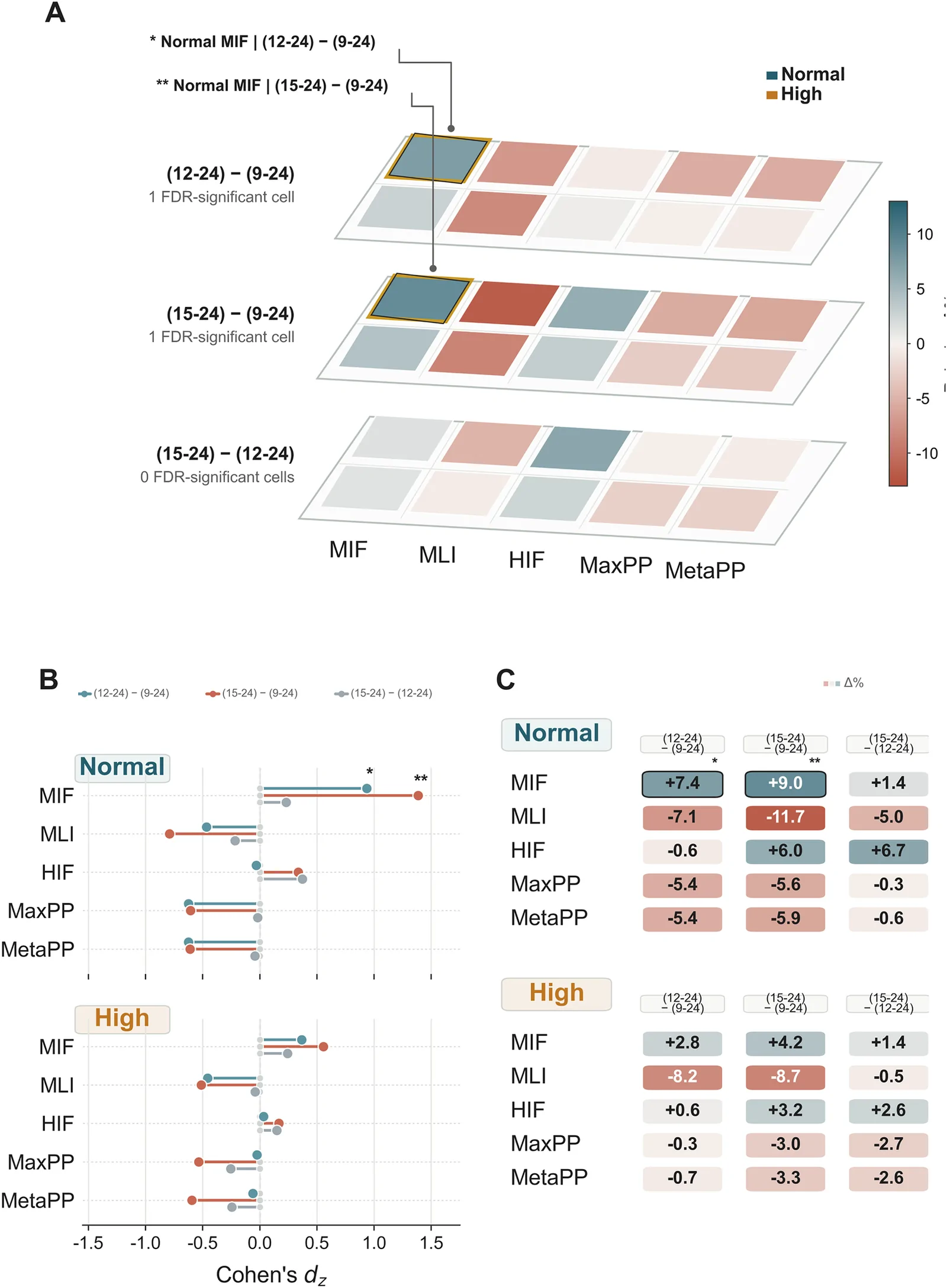
Prespecified within-arch comparisons among the selected support configurations. **(A)** Group-mean percentage differences for (12–24) − (9–24), (15–24) − (9–24), and (15–24) − (12–24), outlined cells indicate *q* < 0.05. **(B)** Paired Cohen's $d_{z}$ values in the same contrast directions; asterisks indicate FDR-supported contrasts and do not rank outcome importance. **(C)** Numerical group-mean percentage differences. Percentages were calculated directly from the corresponding group means using the first-listed condition as the numerator target and the second-listed condition as the denominator reference (*q < 0.05; **q < 0.01). MIF, midfoot impulse fraction; HIF, heel impulse fraction; MLI, mediolateral impulse index; MaxPP, maximum regional peak pressure; MetaPP, maximum metatarsal-region peak pressure.

Within the normal-arch group, MIF was 7.4% higher under the 12–24 condition than under the 9–24 condition (*q* = 0.041, Cohen's *d_z_* = 0.94) and 9.0% higher under the 15–24 condition than under the 9–24 condition (*q* = 0.003, *d_z_* = 1.39). MLI was 11.7% lower under the 15–24 condition than under the 9–24 condition (*q* = 0.085, *d_z_* = −0.79); however, this contrast did not reach statistical significance after FDR correction. No other pairwise comparison in the normal-arch group reached FDR-corrected significance. For the direct comparison between 15–24 and 12–24, none of the five primary outcomes reached FDR-corrected significance in the normal-arch group. Likewise, none of the pairwise support-height comparisons reached FDR-corrected significance for any primary outcome in the high-arch group. Thus, the statistically supported differences among support configurations were confined to the higher MIF observed under 12–24 and 15–24 relative to 9–24 in the normal-arch group (Figures 5A–C).

### 3.5 Support-response patterns and between-arch-group comparisons

Across the three support conditions, group-mean changes relative to Single followed the same directions in both arch groups: MIF increased, whereas MLI, HIF, MaxPP, and MetaPP decreased. MIF differences ranged from +9.7% to +19.5% in the normal-arch group and from +18.0% to +23.0% in the high-arch group; MLI differences ranged from −24.0% to −14.0% and from −19.7% to −12.0%, respectively. Across both groups and all three conditions, HIF, MaxPP, and MetaPP differences ranged from −16.0% to −9.3%, −9.4% to −3.3%, and −9.0% to −3.2%, respectively (Figure 6A).

**Figure 6:**
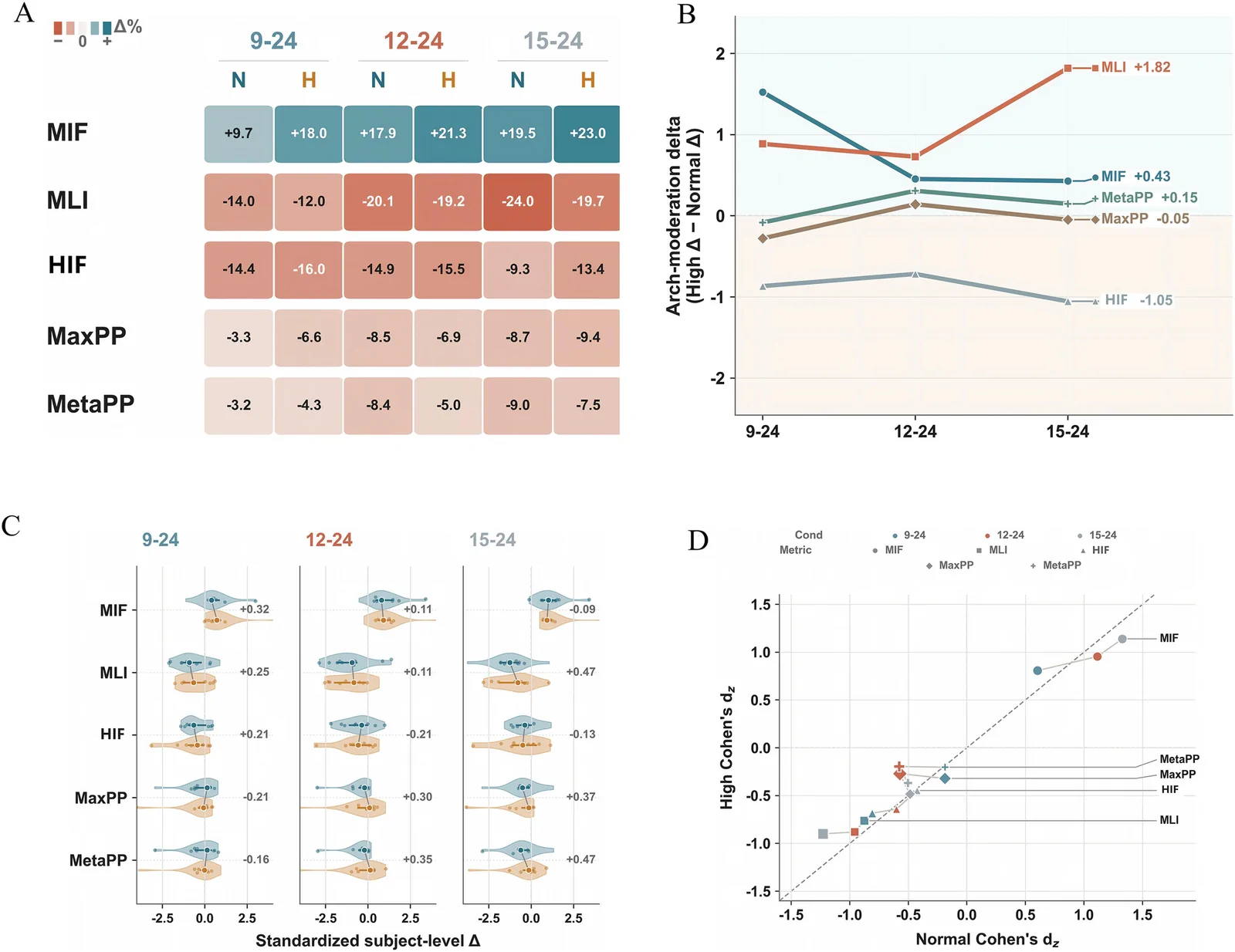
Support-condition response patterns and between-arch-group comparisons. **(A)** Group-mean percentage differences under each support condition relative to Single. **(B)** Between-arch-group differences in participant-level Δ (High − Normal), where Δ = support condition − Single; MIF, HIF, and MLI are in percentage points and MaxPP/MetaPP in N/cm^2^. **(C)** Descriptive distributions of pooled z-standardized participant-level Δ values; annotations are High minus Normal median differences. **(D)** Numerical correspondence of within-group Cohen's *d_z_* values; the dashed identity line denotes equal numerical values.

Participant-level changes were defined as Δ = support condition − Single and compared between arch groups as High Δ − Normal Δ. None of the 15 height-specific comparisons met the FDR threshold (all *p* ≥ 0.298; all *q* = 0.934), and every 95% confidence interval included zero. The largest absolute Hedges’ *g* was 0.38 for MIF under 9–24 (between-arch difference +1.52 percentage points, 95% CI −1.42 to 4.47; *p* = 0.298). Under 15–24, the between-arch differences were +1.82 percentage points for MLI (95% CI −2.71 to 6.35; *p* = 0.418; *g* = 0.29) and −1.05 percentage points for HIF (95% CI −4.04 to 1.93; *p* = 0.471; *g* = −0.26). Across MaxPP and MetaPP, absolute between-arch Δ differences did not exceed 0.31 N/cm^2^ (all *p* ≥ 0.626; Figure 6B; Supplementary Table S19).

The standardized participant-level Δ distributions showed visual overlap between arch groups, with the annotated High-minus-Normal median differences ranging from −0.21 to 0.47 across the 15 outcome-by-condition comparisons (Figure 6C). Under 15–24, the within-group paired Cohen's *d_z_* values in the normal- and high-arch groups were 1.33 and 1.14 for MIF, −1.23 and −0.90 for MLI, −0.42 and −0.45 for HIF, −0.49 and −0.48 for MaxPP, and −0.50 and −0.37 for MetaPP, respectively (Figure 6D).

As a complementary omnibus analysis, linear mixed-effects models were fitted for each of the five primary outcomes. No significant Arch Type × Condition interaction was detected for MaxPP [*χ*^2^(3) = 0.884, *p* = 0.829], MetaPP [*χ*^2^(3) = 0.859, *p* = 0.835], MIF [*χ*^2^(3) = 2.339, *p* = 0.505], HIF [*χ*^2^(3) = 1.162, *p* = 0.762], or MLI [*χ*^2^(3) = 1.060, *p* = 0.787]. After BH-FDR correction across the five interaction tests, all *q* values were 0.835 (Supplementary Table S13).

### 3.6 Sensitivity analysis of the arch type × condition interaction

To evaluate whether the omnibus interaction inference was sensitive to the balanced sampling of the normal-arch group, the mixed-effects models were repeated in the full cohort of 55 eligible runners (40 normal-arch and 15 high-arch runners; 220 participant-condition observations) using the same model specification as in the balanced 15:15 analysis. Re-fitting the models in the balanced cohort reproduced the previously reported Arch Type × Condition results for all five primary outcomes. None of the omnibus interactions reached the FDR threshold in the balanced analysis: MaxPP, *χ*^2^(3) = 0.884, *p* = 0.829, *q* = 0.835; MetaPP, *χ*^2^(3) = 0.859, *p* = 0.835, *q* = 0.835; MIF, *χ*^2^(3) = 2.339, *p* = 0.505, *q* = 0.835; HIF, *χ*^2^(3) = 1.162, *p* = 0.762, *q* = 0.835; and MLI, *χ*^2^(3) = 1.060, *p* = 0.787, *q* = 0.835.

After inclusion of all 40 eligible normal-arch runners, the Arch Type × Condition interactions remained non-significant for MaxPP [*χ*^2^(3) = 1.746, *p* = 0.627, *q* = 0.990], MetaPP [*χ*^2^(3) = 0.907, *p* = 0.824, *q* = 0.990], MIF [*χ*^2^(3) = 4.059, *p* = 0.255, *q* = 0.990], HIF [*χ*^2^(3) = 2.918, *p* = 0.405, *q* = 0.990], and MLI [*χ*^2^(3) = 0.116, *p* = 0.990, *q* = 0.990] (Table 2). Thus, none of the five primary outcomes showed an FDR-supported omnibus interaction in either the balanced or full-cohort analysis, indicating that the principal inference concerning Arch Type × Condition was not materially altered by inclusion of the additional normal-arch participants.

**Table 2:** Sensitivity analysis of the arch type × condition interaction in the balanced and full cohorts.

Outcome	Balanced 15:15 *χ*^2^ (3)	*p*	*q*	Full 40:15 *χ*^2^ (3)	*p*	*q*
MaxPP	0.884	0.829	0.835	1.746	0.627	0.990
MetaPP	0.859	0.835	0.835	0.907	0.824	0.990
MIF	2.339	0.505	0.835	4.059	0.255	0.990
HIF	1.162	0.762	0.835	2.918	0.405	0.990
MLI	1.060	0.787	0.835	0.116	0.990	0.990

Separate maximum-likelihood random-intercept mixed-effects models were fitted for each primary outcome. The omnibus Arch Type × Condition interaction was evaluated using a likelihood-ratio comparison between the full model and the corresponding reduced model without the interaction term (df = 3). Benjamini–Hochberg false-discovery-rate correction was applied across the five omnibus interaction tests separately within each analysis frame. The balanced analysis included 15 normal-arch and 15 high-arch runners, whereas the full-cohort sensitivity analysis included 40 normal-arch and 15 high-arch runners. No omnibus interaction met the FDR threshold in either analysis. *q*, Benjamini–Hochberg FDR-adjusted *p* value.

The full-cohort models generally provided more precise interaction estimates, although individual model-based contrasts should be interpreted cautiously. In particular, the MIF interaction contrast at 9–24 was +1.50 percentage points (95% Wald CI 0.01–3.00; unadjusted *p* = 0.049), whereas the corresponding three-degree-of-freedom omnibus MIF interaction remained non-significant (*p* = 0.255, *q* = 0.990), and this individual contrast did not remain significant after multiplicity adjustment. Accordingly, this isolated nominal result was not interpreted as evidence of a systematic arch-type-specific response. Overall, the full-cohort sensitivity analysis did not provide additional evidence that static arch type modified the overall pattern of immediate loading responses across the three support configurations. These nonsignificant interaction findings should not be interpreted as evidence of equivalence between the two arch groups.

### 3.7 Temporal distribution of shoe–ground loading differences during stance

The exploratory time-series analysis temporally localized pointwise differences between each support condition and Single. BH-FDR correction was applied separately to the 91 tests within each of the 18 arch-group × condition × temporal-metric curves; contiguous *q* < 0.05 points are reported as sampling-point runs (Figure 7; Supplementary Table S12).

**Figure 7:**
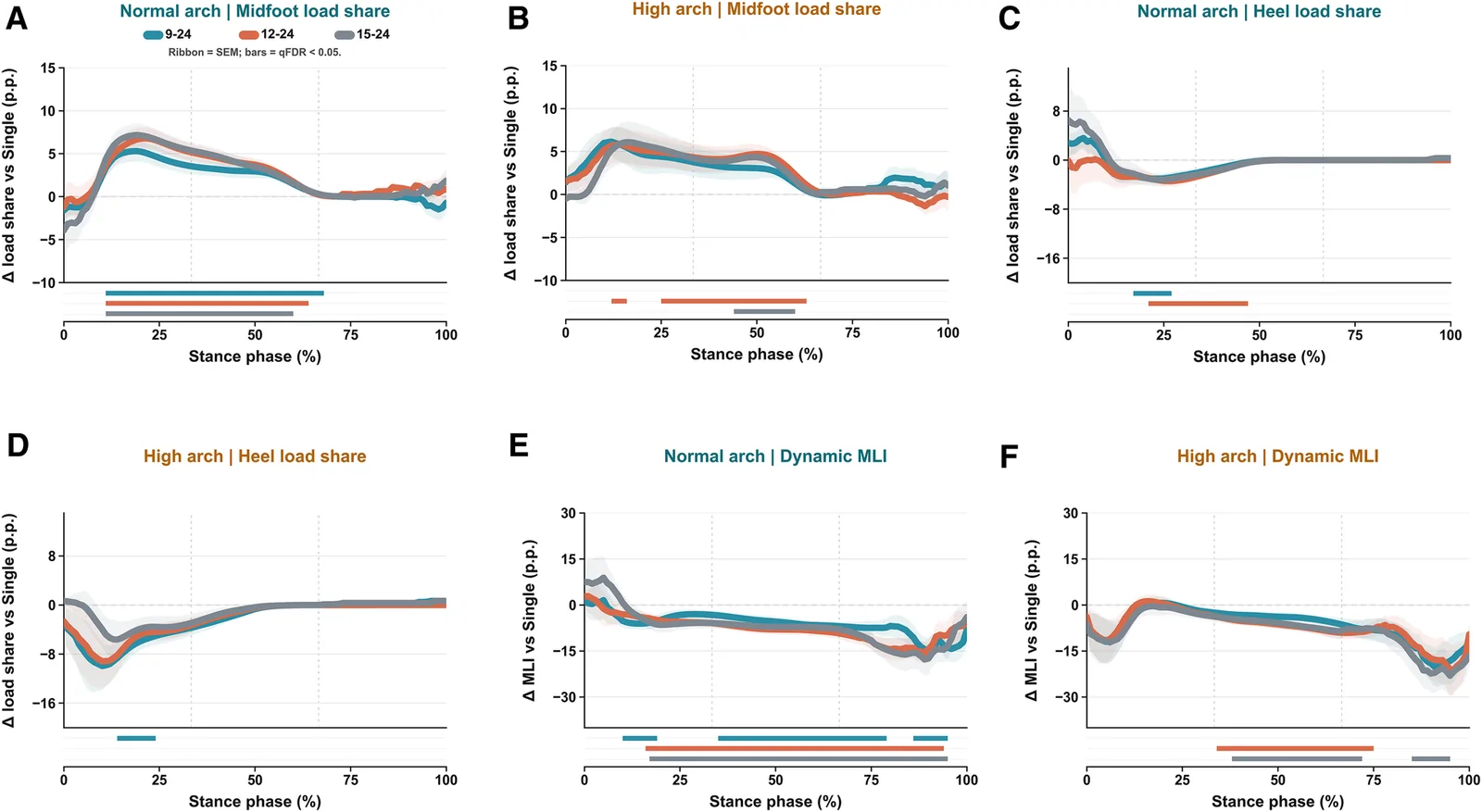
Exploratory pointwise time-series differences relative to Single across normalized stance. The curves represent mean support-minus-Single differences for the 9–24, 12–24, and 15–24 conditions, and the shaded regions indicate the standard error of the mean. Horizontal bars denote sampling-point runs with q<0.05 after pointwise BH-FDR correction. Panels show instantaneous midfoot force share [MFS(t)] **(A,B)**, instantaneous heel force share [HFS(t)] **(C,D)**, and dynamic mediolateral force index [dynamic MLI(t)] **(E,F)** for normal- and high-arch runners.

For MFS(t), the normal-arch group showed higher values than Single at 11%–68%, 11%–64%, and 11%–60% stance under 9–24, 12–24, and 15–24, respectively. In the high-arch group, no point met *q* < 0.05 under 9–24; runs occurred at 12%–64% under 12–24 and 11%–62% under 15–24 (Figures 7A,B). For HFS(t), the normal-arch group showed lower values at 17%–27% under 9–24 and 21%–47% under 12–24, with no *q* < 0.05 points under 15–24. In the high-arch group, lower values occurred at 15%–24% under 9–24; no point met *q* < 0.05 under 12–24 or 15–24 (Figures 7C,D). For dynamic MLI(t), the normal-arch *q* < 0.05 runs were 10%–19%, 35%–79%, and 86%–95% under 9–24; 16%–94% under 12–24; and 17%–95% under 15–24. In the high-arch group, no point met *q* < 0.05 under 9–24; runs occurred at 31%–76% under 12–24 and at 10%–11%, 33%–73%, and 84%–95% under 15–24 (Figures 7E,F).

The first-three-valid-trials-per-foot sensitivity analysis retained the principal MFS(t) and dynamic MLI(t) temporal patterns, with shifts in several run boundaries; the HFS(t) *q* < 0.05 runs were not reproduced (Supplementary Tables S11 and S12; Supplementary Figure S1).

## 4 Discussion

### 4.1 Baseline shoe–ground interface loading characteristics of normal- and high-arched runners

Under the Single condition, none of the five primary shoe–ground interface loading outcomes differed between the selected normal- and high-arch groups after BH-FDR correction. This finding should not be interpreted as evidence that the two groups had identical loading phenotypes. Previous studies based on static plantar contact or standing measurements have reported smaller midfoot contact areas and region-specific differences in forefoot or rearfoot pressure in individuals with high or cavus arches (30, 31). The present study differs from those investigations in both the locomotor task and the measurement context: participants were experienced marathon runners performing shod running, and the recorded signals represented the external shoe–ground interface rather than direct plantar-skin loading.

The arch-group stratification used in the present study was also based on static footprint morphology. Although this approach provides a structured static morphological classification, it does not directly characterize dynamic arch deformation, arch stiffness, or other functional properties that may emerge during running. Consequently, the absence of FDR-significant baseline differences in the five primary outcomes indicates that static footprint-based arch morphology was not strongly reflected in these selected dynamic shoe–ground loading measures under the present testing conditions; it does not establish broader biomechanical equivalence between the two arch groups. Whether their condition-related responses differed was therefore evaluated directly using participant-level change comparisons and complementary Arch Type × Condition models.

### 4.2 Shoe–ground interface loading differences observed under arch-support conditions and complementary metric responses

In the primary balanced analysis, the clearest and most consistent FDR-supported condition-related differences in both arch groups involved higher MIF and lower MLI relative to Single. HIF was also numerically lower across the support conditions, with FDR-supported differences under 9–24 in both groups. In this primary analysis, no MaxPP or MetaPP support-vs.-Single comparison met the FDR threshold. Accordingly, MIF and MLI showed more consistently FDR-supported condition-related differences than the peak-pressure measures in the primary balanced sample. The full-cohort sensitivity analysis yielded additional FDR-supported support-vs.-Single comparisons, driven principally by the expanded normal-arch sample and including selected MaxPP and MetaPP contrasts. Thus, the exact set of FDR-supported within-group comparisons was not invariant to the analysis frame and should not be interpreted as a fixed ranking of outcome responsiveness. Importantly, the additional within-group findings did not translate into FDR-supported between-arch differences in condition-related change or omnibus Arch Type × Condition interactions.

Peak-pressure and impulse-based metrics characterize complementary aspects of loading. MaxPP and MetaPP quantify the highest regional pressure reached during stance, whereas impulse incorporates both force magnitude and duration (14, 32). Accordingly, differences in the duration or relative contribution of regional loading may be reflected in MIF, HIF, or MLI without requiring a parallel difference in peak pressure. MIF represents the relative midfoot contribution to total stance-integrated impulse, whereas MLI describes the signed relative contribution of the predefined medial and lateral impulse sets. These measures therefore characterize regional load composition and should not be interpreted as direct measures of absolute regional unloading, tissue stress, or clinical benefit.

Comparable arch-support studies provide relevant context for this pattern. Cheng et al. reported that different arch-support heights during running were associated with lower peak pressure in the medial forefoot and rearfoot and higher pressure in the medial midfoot and lateral foot (7). Ma et al. similarly reported changes in medial-forefoot and heel loading with personalized orthotic configurations in runners with subtle cavus feet (11), whereas Peng et al. showed that regional plantar-pressure patterns during loaded uphill walking varied with orthotic configuration and material properties (33). The reviewer-recommended study by Channasanon et al. further showed in a 1-month randomized crossover study that both 3D-printed and prefabricated medial arch supports were associated with higher midfoot average pressure and lower forefoot and hindfoot pressure (34), whereas plantar-pressure distribution did not differ significantly among the tested supports despite differences in hardness. Collectively, these findings support the interpretation that arch support can reorganize regional loading, while also indicating that a single structural parameter does not necessarily determine the resulting pressure pattern. Complementing this pressure-based evidence, Chen et al. reported in adults with flexible flatfoot that increasing arch-support hardness was associated with improvements in several rearfoot biomechanical measures and lower tibialis posterior loading, but that these responses tended to plateau at higher hardness and were accompanied by increased knee and metatarsophalangeal loading (35).

The present findings extend this perspective by showing that the selected shoe–ground interface measures did not change uniformly across metric types. Specifically, the condition-related differences were expressed most consistently through MIF and MLI, whereas the peak-pressure measures provided less consistent inferential separation from Single. Thus, peak pressure and regional impulse composition should be regarded as complementary rather than interchangeable descriptors when characterizing immediate shoe–ground loading across arch-support conditions.

### 4.3 Foot-arch type, support-height responses, and configuration implications

The descriptive directions of the condition-related changes were broadly similar in the normal- and high-arch groups. Across the three selected support conditions, MIF was generally higher and MLI lower than under Single in both groups, while HIF, MaxPP, and MetaPP were generally numerically lower. However, similarity in descriptive direction does not establish statistical equivalence, and differences between within-group significance patterns cannot by themselves demonstrate an arch-type-specific response.

Direct between-arch comparisons provided the appropriate test of this question. In the balanced sample, none of the 15 height-specific comparisons of participant-level change from Single met the FDR threshold, and all corresponding confidence intervals included zero. Likewise, none of the five complementary Arch Type × Condition interactions reached FDR-adjusted significance. This principal arch-specific inference was retained in the full-cohort sensitivity analysis: after inclusion of all 40 eligible normal-arch runners, neither the 15 between-arch Δ comparisons nor the five omnibus Arch Type × Condition interactions yielded FDR-supported evidence of an arch-specific response. Although the larger normal-arch sample changed the significance of some within-normal-arch comparisons, the analyses directly addressing arch-related effect modification remained concordant across the two analysis frames. Taken together, the present analyses provide insufficient statistical evidence that static arch type systematically modified either the magnitude of the height-specific changes or the overall condition-related response pattern. These nonsignificant findings should not be interpreted as evidence that the two arch groups responded equivalently. One nominally significant full-cohort MIF interaction coefficient at 9–24 did not alter this interpretation because it was not supported by the corresponding omnibus interaction, multiplicity-adjusted model-based inference, or the prespecified participant-level change-score comparison.

Direct comparisons among the selected support conditions also provide a more constrained interpretation of configuration differences. In the primary balanced analysis, FDR-supported differences in the normal-arch group were detected only for MIF, which was higher under 12–24 than under 9–24 and under 15–24 than under 9–24. No primary outcome differed significantly between 12–24 and 15–24 after FDR correction, and no direct support-condition comparison reached the FDR threshold in the high-arch group. The full-cohort sensitivity analysis identified additional FDR-supported configuration contrasts within the expanded normal-arch sample, indicating that some configuration-specific within-group findings were sensitive to the greater precision of the larger sample. Nevertheless, no primary outcome differed significantly between 12–24 and 15–24, and no FDR-supported configuration contrast emerged in the high-arch group in either analysis frame. Accordingly, the present analyses do not support a simple monotonic dose–response interpretation or establish general superiority of either the 12–24 or 15–24 configuration. These two configurations may nevertheless be retained as candidate conditions for further investigation.

Related studies further indicate that biomechanical responses to arch support are influenced by multiple design characteristics rather than by a single structural parameter. Channasanon et al. reported that 3D-printed and prefabricated medial arch supports were associated with regional plantar-pressure redistribution (34), while differences in support hardness did not translate into clear differences in plantar-pressure distribution among the tested supports. Chen et al. further reported that increasing arch-support hardness in adults with flexible flatfoot was associated with changes in rearfoot biomechanics and tibialis posterior loading (35), with several responses tending to plateau at higher hardness and with concurrent changes in loading at other joints. These studies manipulated orthotic hardness rather than support height and involved populations, locomotor tasks, and orthosis designs that differ from the present running experiment. They therefore do not directly confirm the present 12–24 or 15–24 findings. Instead, together with the present findings, they illustrate that orthotic responses are multidimensional and may depend on interactions among geometry, structural properties, locomotor task, and individual characteristics rather than following a simple monotonic relationship with one design parameter (36). The present biomechanical findings alone are insufficient to identify an optimal support configuration. Comfort, preference, and longer-term adaptation should be evaluated in future studies specifically designed for configuration selection (37).

### 4.4 Exploratory temporal distribution of shoe–ground force-composition differences during stance

The exploratory time-series analysis was used to localize when condition-related differences relative to Single occurred during normalized stance. The temporal curves were normalized according to percentage of stance rather than aligned to discrete gait events (38). Therefore, identical normalized percentages should not be assumed to represent identical functional events across conditions, particularly because support configuration could alter the timing of heel loading, midfoot contact, heel-off, or propulsion. The reported temporal locations should therefore be interpreted as normalized-time localizations rather than event-registered phase effects. Pointwise BH-FDR correction was applied separately to the 91 tests within each Arch Type × Condition × temporal-metric curve. Consequently, contiguous sampling points with *q* < 0.05 represent within-curve exploratory temporal localization only; they do not constitute statistical clusters, phase-wide effects, or whole-curve significance. The duration of these runs is descriptive and was not used to compare response strength between support conditions or arch groups.

Within this framework, the temporal locations differed among the three force-composition measures. For MFS(t), *q* < 0.05 sampling-point runs occurred mainly from early to middle stance in the normal-arch group under all three selected support conditions; in the high-arch group, corresponding runs were present under 12–24 and 15–24 but not under 9–24. HFS(t) differences were confined to earlier portions of stance, occurring under 9–24 and 12–24 in the normal-arch group and under 9–24 in the high-arch group. Dynamic MLI(t) showed *q* < 0.05 sampling-point runs primarily across middle and later portions of stance, although their exact locations varied across conditions and arch groups. These observations indicate when the corresponding support-vs.-Single differences were detected within stance, rather than demonstrating stronger or more persistent responses for one support condition or arch group.

The first-three-valid-trials-per-foot sensitivity analysis broadly reproduced the principal temporal patterns for MFS(t) and dynamic MLI(t), although several sampling-run boundaries shifted. In contrast, the HFS(t) *q* < 0.05 runs observed in the main analysis were not reproduced in the first-three-trial frame. The temporal interpretation of the heel-force-share findings should therefore remain more tentative than that of the MFS(t) and dynamic MLI(t) patterns.

Naderi et al. likewise reported that orthosis-related changes in dynamic in-shoe pressure were temporally localized to particular portions of stance rather than occurring uniformly throughout the gait cycle (39). Their population, measurement interface, orthotic design, and analytical approach differed from those used here, so direct correspondence of specific temporal intervals should not be inferred. The present time-resolved analysis instead complements the stance-integrated outcomes by identifying where within stance the observed shoe–ground force-composition differences relative to Single were located. No direct time-series statistical comparison was performed between support heights or between arch groups.

### 4.5 Limitations and future directions

Several considerations define the scope of the present findings. First, this study was an arch-type-stratified secondary analysis of pre-existing trials from the original multi-condition experimental campaign. Because the Single condition preceded the extracted support conditions, differences relative to Single should be interpreted as condition-related differences rather than isolated causal effects of support height. The balanced analysis included all 15 eligible high-arch runners and an outcome-blind random sample of 15 of the 40 eligible normal-arch runners; this sampling strategy reduced the statistical precision available for between-arch and interaction analyses. The full-cohort sensitivity analysis reduced concern that the principal arch-specific inference depended on this normal-arch subsampling; however, the high-arch group remained limited to 15 runners, and smaller arch-dependent effects cannot be excluded. Although all mixed-effects models converged, residual diagnostics showed departures from residual normality for several outcomes; the LMM results were therefore interpreted as complementary to the prespecified participant-level change analyses. Second, arch classification was based on a two-stage static morphological assessment. Arch angle and the Chippaux-Smirak index were used as supporting descriptors without prespecified numerical cutoffs, and formal intra- or inter-rater reliability was not assessed in the original experimental campaign. Thus, the present grouping represents static arch morphology rather than directly measured dynamic arch deformation or stiffness. In addition, the floor-mounted Footscan® system quantified shoe–ground interface loading rather than plantar-skin or in-shoe pressure. Finally, running speed was controlled using participant-specific target pace and sports-watch monitoring, but instantaneous approach speed at pressure-plate contact was not independently measured. The time-series analysis was used to localize condition-related differences during stance and was not treated as a separate confirmatory analysis independent of the primary discrete-outcome analyses.

Within these boundaries, the findings provide several practical considerations for the biomechanical evaluation and subsequent development of 3D-printed arch-support inserts. Across the selected support conditions, the most consistent differences were observed in regional impulse composition, particularly higher MIF and lower MLI, whereas MaxPP and MetaPP did not show consistent FDR-supported differences. This indicates that evaluation based on peak pressure alone may overlook condition-related information captured by stance-integrated regional impulse measures. The present findings support retaining 12–24 and 15–24 as candidate configurations for further evaluation rather than ranking either as an optimal or generally superior height. Moreover, the absence of FDR-supported between-arch change differences and Arch Type × Condition interactions in both the balanced and full-cohort analyses indicates that static normal-vs.-high arch classification alone did not provide sufficient evidence for selecting different support heights in the present sample. Future configuration studies should employ direct measurements of running speed or instrumented treadmills capable of simultaneously recording ground-reaction forces, while also incorporating dynamic arch deformation or stiffness, subjective comfort and preference, and longer-term adaptation. These measures would provide a stronger basis for individualized configuration recommendations.

## 5 Conclusion

In this arch-type-stratified secondary analysis, no FDR-significant differences were detected between the selected normal- and high-arch groups for the five primary shoe–ground interface loading outcomes under the Single condition. Across the three selected support conditions, the main differences observed relative to Single were characterized by higher midfoot impulse fractions and lower mediolateral impulse indices, whereas no MaxPP or MetaPP support-vs.-Single comparison met the FDR threshold. Within the normal-arch group, FDR-significant direct differences among support conditions were detected for MIF for 12–24 vs. 9–24 and 15–24 vs. 9–24, whereas no direct support-condition comparison remained significant in the high-arch group. The current analyses did not establish general superiority of either the 12–24 or 15–24 configuration. Neither the height-specific between-arch Δ comparisons nor the complementary omnibus Arch Type × Condition analyses provided sufficient statistical evidence that the magnitude or overall pattern of condition-related changes differed between the two arch groups; these nonsignificant findings should not be interpreted as evidence of equivalence. Because the present study was a secondary analysis of pre-existing trials from the original multi-condition experiment and did not introduce new randomization, the observed between-condition differences should not be interpreted as isolated causal effects of support height. MIF and MLI may provide complementary information to peak-pressure measures when characterizing immediate shoe–ground interface loading. Future configuration studies should incorporate prospective experimental designs together with dynamic arch deformation, arch stiffness, comfort, preference, and longer-term adaptation.

## Data Availability

The raw data supporting the conclusions of this article are not publicly available due to privacy and ethical restrictions. The ethical approval and informed consent obtained for this study do not permit public sharing of participant-level data. De-identified data may be made available by the corresponding author upon reasonable request, subject to approval by the relevant ethics committee, applicable institutional and data-protection requirements, and, where applicable, execution of a data-use agreement. Approved data may not be redistributed or used beyond the approved purpose without additional written authorization. Requests should be directed to mayong@whsu.edu.cn.
