# Supplementary material for: Shoe–ground loading responses across three 3D-printed arch-support heights in normal- and high-arched marathon runners: an arch-type-stratified secondary analysis
Source: Front Sports Act Living. 2026 Sep 24;8:1942713. doi: 10.3389/fspor.2026.1942713 (PMC13648250; doi:10.3389/fspor.2026.1942713)
Supplement: Supplementary file 1 [file Datasheet1.pdf]

Supplementary Material

Supplementary Methods

Scope. This file documents the data-source governance, outcome derivation, statistical-unit construction, quality-control procedures, and auxiliary analyses implemented in the analysis pipeline. It is intended to be read with the main manuscript. Terms and abbreviations are kept identical to those used in the manuscript.

Supplementary CAD files

The three-dimensional surface geometries of the selected arch-support insert models are provided in the accompanying archive ***Supplementary_CAD_Files_S1-S3.zip***. The archive contains three separate STL files: ***Supplementary_CAD_File_S1_H9.stl*** for the H9/D24 configuration, ***Supplementary_CAD_File_S2_H12.stl*** for the H12/D24 configuration, and ***Supplementary_CAD_File_S3_H15.stl*** for the H15/D24 configuration. The STL files document the corresponding insert surface geometries; the 24% infill density was a manufacturing parameter and is therefore not encoded in the STL surface geometry. For each nominal support height, the contralateral insert used experimentally was the mirror-image counterpart of the supplied model. Principal geometric dimensions are summarized in **Supplementary Table S14.**

S1. Data-source governance and analysis roles

The analysis pipeline read six consolidated workbooks and one demographic workbook. The consolidated pressure-force workbook was the sole inferential source for the five primary discrete outcomes. Original Footscan exports were not appended to the consolidated trial table and were not used to replace missing primary statistical units under the default source policy. Original pressure-force exports were, however, parsed separately for the exploratory regional force-time analysis used to localize temporal response patterns. CoP, timing, and regional-area workbooks were processed as secondary data streams for audit or supplementary description and did not enter the five-outcome primary inferential analyses. Data-source roles are summarized in Supplementary Table S1. For readability, the supplementary tables use reader-facing data labels; exact machine filenames and code-level variable names are retained in the reproducibility records described in S9.

S2. Label standardization and trial-grid validation

Condition labels were standardized as Single, 9-24, 12-24, and 15-24. Subject identity, arch type, foot side, and trial number were standardized before merging. The consolidated primary pressure-force data were required to form a complete 30 participant × 4 condition × 2 foot × 3 trial grid. Duplicate primary statistical keys or incomplete primary trial cells triggered analysis termination and an audit file. Secondary CoP, timing, SPM-CoP, and regional-area tables were checked against the same expected trial grid; secondary-table problems generated warnings or source-specific repair audits rather than altering the primary pressure-force table.

S3. Primary pressure-force quality control

Behavioral invalidity (e.g., obvious turning, targeting the plate, incomplete foot contact, or device non-triggering) was determined before inclusion in the consolidated dataset from testing records and footprint completeness. The script subsequently applied numerical checks to each retained pressure-force trial. Total impulse was required to be greater than 0; MaxPP was required to be between 0 and 80 N/cm²; and, when available, stance time was required to be between 100 and 1,200 ms. A failed primary trial caused the pipeline to stop for manual review. Statistical outlier flags did not automatically remove observations.

S4. Outcome derivation and statistical-unit construction

Regional peak pressure and force impulse were available for 10 functional regions: T1, T2-5, M1-M5, MF, HM, and HL. Primary outcomes were calculated at the individual-trial level. Within each participant, condition, and foot, the three retained trial values were averaged. Left- and right-foot means were then averaged with equal weight to form one Participant × Condition observation. The exact formulas, region sets, units, and interpretation limits are provided in Supplementary Tables S2 and S3.

S5. Change metrics and visualization quantities

The participant-level absolute response was defined as $\Delta_{i,c} = X_{i,c} - X_{i,\text{Single}}$. This absolute change was used for participant-level response displays and for arch-group comparisons at each support height. Percentage changes displayed in the principal figures were calculated from complete-pair group means: 100 × (mean condition − mean reference)/mean reference. The mean of individual percentage ratios was retained only as an audit descriptor because ratios can be unstable when individual reference values are small.

S6. Exploratory force-time analysis

Regional force-time curves were parsed from original Footscan pressure-force exports. The default main frame used all successfully parsed trials available for each foot; a first-three-trials-per-foot frame was generated as a sampling-frame sensitivity analysis. Each trial curve was normalized to 0%-100% stance and resampled to 101 points. Curves were first averaged across trials within foot and then averaged equally across feet to produce Participant × Condition × Time curves. Pointwise testing was limited to 5%-95% stance because force-share ratios can be unstable near initial contact and toe-off. Dynamic variables are instantaneous force-composition metrics and are not interchangeable with stance-integrated MIF or HIF.

S7. Statistical diagnostics, robustness analyses, and omnibus mixed-effects model

Parametric tests constituted the primary discrete-outcome analyses. Distributional assumptions were evaluated using Shapiro–Wilk tests, with paired-difference distributions examined for paired contrasts and group-specific distributions examined for independent contrasts. All t tests, Wilcoxon signed-rank tests, and Mann–Whitney U tests were two-sided. Wilcoxon signed-rank and Mann–Whitney U tests were used as rank-based robustness analyses. Potentially influential observations were identified using the 1.5 × IQR rule and an absolute modified Z score greater than 3.5. Trial inclusion remained governed by the predefined technical-validity criteria rather than by statistical extremeness alone.

Because participant S07 in the high-arch group showed a visually distinct MIF response, a targeted participant-level robustness analysis was conducted. Four standalone raw exports were compared with their copies in the 30-participant archive by SHA-256 hash, parsed independently, and audited for finite force/pressure signals, positive total impulse, regional MaxPP within 0–80 N/cm², export-derived stance duration within 100–1,200 ms, first-three-trial reconstruction, repeated-trial consistency, and bilateral consistency. Behavioral criteria that require contemporaneous testing records or footprint images were not inferred from the numerical exports. Rank-based Wilcoxon signed-rank analyses retaining all 15 high-arch participants were then applied to the support-versus-Single contrasts, followed by a post hoc leave-S07-out influence analysis with n = 14. Both analyses retained the original 30-test support-versus-Single multiplicity structure, and the full n = 15 high-arch dataset remained the primary inferential sample. Detailed results are reported in Supplementary Table S16.

To evaluate whether the overall condition-related pattern differed by arch type, a linear mixed-effects model was fitted separately for each of the five primary outcomes using 120 participant-condition observations (30 participants × 4 conditions). The model was Outcome ~ Arch Type + Condition + Arch Type × Condition + (1 | Participant), with Arch Type, Condition, and their interaction as fixed effects and participant as a random intercept. Models were estimated by maximum likelihood. The omnibus interaction was evaluated by likelihood-ratio comparison with a reduced model omitting Arch Type × Condition. The five interaction p values formed a separate five-test BH-FDR family. Complete results for all five outcomes are reported in Supplementary Table S13.

A conditional Monte Carlo design-sensitivity analysis quantified the minimum detectable omnibus interaction for the analyzed balanced sample. The interaction vector comprised the High-minus-Normal differences in the three support-minus-Single changes. Using the full-model residual variance for each outcome, the exact contrast covariance implied by the random-intercept LMM, 200,000 replicates, and a target power of 80%, the smallest detectable maximum interaction coefficient was estimated for three declared shapes: a common support shift, linear divergence, and a difference localized to one support condition. Nominal $\alpha = 0.05$ and a conservative $p \leq 0.01$ benchmark were evaluated. The latter is sufficient for BH $q \leq 0.05$ in a five-test family regardless of the other four p values, but it is not an exact BH power calculation. This analysis quantified the detection limits of the n = 30 design; it was not used as observed power, an all-55 reanalysis, or an equivalence test (Supplementary Table S20).

Full-cohort sensitivity extension.

To assess whether the principal arch-specific conclusions depended on the balanced sampling of 15 of the 40 eligible normal-arch runners, a full-cohort sensitivity frame was constructed using all 55 eligible runners (40 normal arch and 15 high arch; 220 participant-condition observations). The balanced 15:15 sample remained the primary analytic frame. The sensitivity analysis changed the sample frame only: the five primary outcomes, participant-condition statistical unit, condition definitions, effect directions, and trial-to-foot-to-participant aggregation rules were retained.

The four prespecified discrete analysis families were repeated in the full 40:15 cohort using the same tests and BH-FDR families as in the balanced analysis. Single baseline and High Δ − Normal Δ comparisons used Welch independent-samples t tests with Hedges' g; support-versus-Single and direct support-configuration contrasts used paired-samples t tests with Cohen's dz. Thus, the full-cohort discrete analyses evaluated sample-selection sensitivity of the original inferential framework rather than defining a new primary analysis.

The complementary omnibus LMM was also repeated without changing model specification: Outcome ~ Arch Type + Condition + Arch Type × Condition + (1 | Participant), with Normal arch and Single as reference levels and maximum-likelihood estimation. For each outcome, the Arch Type × Condition interaction was tested using a 3-df likelihood-ratio comparison of the full and reduced models. BH-FDR correction was applied across the five omnibus interaction tests separately within the balanced and full-cohort frames. The mixed-effects models remained complementary omnibus analyses and did not replace the prespecified discrete-outcome comparisons.

For transparency, the three model-based Arch Type × Condition interaction coefficients for each outcome were reported as (High support − High Single) − (Normal support − Normal Single), i.e., model-based High Δ − Normal Δ contrasts at 9-24, 12-24, and 15-24, with nominal asymptotic 95% Wald confidence intervals and unadjusted Wald p values. A descriptive BH adjustment across the 15 individual interaction coefficients is reported only as a supplementary multiplicity check; multiplicity-controlled LMM inference remains based on the five prespecified omnibus interaction tests.

Model convergence, random-intercept and residual variances, ICC, conditional-residual normality, residual skewness and excess kurtosis, maximum absolute standardized residual, and residual-scale dependence were examined. Influence was evaluated by omitting the participant associated with the largest absolute standardized residual for each outcome and by a complete participant-level LOO

analysis of the full-cohort omnibus interaction tests. For the prespecified change-score framework, robustness was additionally examined using rank-based tests, LOO analyses of the 15 between-arch Δ comparisons, and 10,000 repeated draws of 15 normal-arch runners from the full pool of 40 while retaining all 15 high-arch runners.

The full-cohort LMMs were estimable and converged, but residual diagnostics indicated departures from Gaussian assumptions for several outcomes and selected evidence of scale dependence. Accordingly, model-based estimates were interpreted cautiously together with the direct participant-level change analyses and robustness checks. These diagnostic departures were not interpreted as making the 55-participant analysis impossible or invalid. Conversely, nonsignificant between-arch Δ or Arch Type × Condition tests were not interpreted as evidence of identical or statistically equivalent responses. No permutation or bootstrap analysis is reported because those procedures were not part of the verified analysis package used for this revision.

S8. Exploratory candidate score

The candidate score was an engineering-oriented descriptive composite, not a validated clinical outcome. It was calculated from MaxPP, MetaPP, MIF, HIF, and |MLI|. Percentage changes were sign-aligned so that lower MaxPP, lower MetaPP, higher MIF, lower HIF, and lower |MLI| contributed positively, and the five aligned values were averaged with equal weight. The score was not subjected to hypothesis testing and must not be used as evidence that one support height is statistically superior.

S9. Reproducibility records

The pipeline version was v12.21, with outputs written to the v12.22 results structure. Exact run-time versions of Python, pandas, NumPy, SciPy, the operating platform, and analysis date were automatically written to the README sheet of the full results workbook and to the analysis log. The output package also retained source-policy manifests, trial-grid audits, quality-control summaries, normality audits, outlier flags, percent-change definition checks, statistical result tables, and figure-specific source workbooks.

S10. Balanced sample construction and baseline comparability

After eligibility screening and arch classification, the sampling frame comprised 40 runners with normal arches and 15 runners with high arches. A sampling file containing only the unique study identifier and arch classification was prepared before the primary plantar-loading outcomes were accessed. All 15 high-arch runners were retained. For the normal-arch pool, a computer-generated random sequence was used to place the 40 study identifiers in random order; the first 15 identifiers in that sequence were selected without replacement. The selected list was then fixed and combined with the 15 high-arch runners to form the balanced 1:1 analytic sample. Selection was based solely on the generated random order and did not use demographic or plantar-loading results. The remaining 25 eligible normal-arch runners were not included in the balanced analytic sample.

The verified demographic workbook contained complete age, height, and body-mass data for all 30 selected participants. Variables were summarized as mean ± standard deviation and compared using two-sided Welch independent-samples t tests. Mean differences were defined as High arch minus Normal arch and reported with 95% confidence intervals and Hedges' g. These comparisons described baseline comparability and were not interpreted as evidence of statistical equivalence.

For descriptive reporting of the static arch-classification descriptors, archived AA and CSI numerical entries were summarized at the participant level. When more than one numerical entry was recorded for a participant, the arithmetic mean of the available entries was used; when a single numerical CSI entry was available, that value was retained as the participant-level descriptor. Group distributions were summarized using mean ± standard deviation, median, interquartile range, and range. These summaries were descriptive and were not used to derive post hoc numerical classification thresholds (Supplementary Table S10, Panel C).

Supplementary Table S1. Data-source governance and analysis-role map.

Data stream	Input source	Analysis object / output	Aggregation level	Role in manuscript	Inferential status
Demographic data	Demographic workbook	Demographic dataset and merge audit	Participant	Participant description and identity audit	Descriptive only
Consolidated pressure-force data	Consolidated pressure-force workbook	Trial-, foot-, and participant-condition pressure-force datasets	Trial → foot → participant × condition	MaxPP, MetaPP, MIF, HIF, and MLI; primary figures, four primary discrete comparison families, and complementary omnibus mixed-effects analysis	Sole primary inferential source
Original regional pressure-force exports	Original Footscan pressure-force files	Long-format force-time data, participant curves, and pointwise results	Trial → foot → participant × condition × time	Exploratory temporal localization of regional force composition	Exploratory pointwise inference; not appended to the primary discrete table
Consolidated CoP scalar data	Consolidated CoP metrics workbook	Participant-condition CoP dataset	Trial → foot → participant × condition	Auxiliary CoP descriptors	Auxiliary/secondary; not part of the five-outcome primary analysis
Consolidated CoP waveforms	Standardized CoP waveform workbooks	Mediolateral and anteroposterior CoP waveforms with repair audit	Trial-level 0%–100% curves	CoP waveform audit or optional supplementary analysis	Secondary; not the source of the regional force-share analysis
Consolidated timing data	Consolidated timing workbook	Participant-condition timing dataset and stance-time quality-control merge	Trial → foot → participant × condition	Stance-time quality control and auxiliary timing descriptors	Auxiliary; stance time used for primary-trial quality control
Original timing exports	Original Footscan timing files	Raw timing audit and conservative missing-cell repair	Raw trial → participant × condition	Provenance audit and exact-key repair of missing consolidated timing cells	Audit/supporting only; existing consolidated values are never overwritten
Consolidated regional-area data	Consolidated regional-area workbook	Participant-condition regional-area dataset and label-repair audit	Trial → foot → participant × condition	Auxiliary contact-area descriptors	Auxiliary only in the present study
Original regional-area exports	Original Footscan regional-area files	Raw regional-area provenance and discrepancy audit	Raw trial → participant × condition	Provenance and discrepancy audit	Audit only; not pooled with curated trials
Optional raw center-of-force waveforms	Original center-of-force waveform exports	Participant waveform and change-curve audit datasets	Trial → participant × condition × time	Optional supplementary waveform audit	Disabled by default; not required for the main results
Static pressure recordings	Static Footscan recordings	Shod and barefoot static-pressure summaries	Participant × condition	Auxiliary description of shod shoe–ground and barefoot skin–ground static interfaces	Descriptive/auxiliary only
Subjective monitoring data	Condition-specific monitoring records	Overall comfort, perceived support, fatigue, pain, and other recorded subjective ratings	Participant × condition	Procedural tolerability monitoring and supplementary descriptive reporting	Procedural/descriptive only; not part of the biomechanical inferential outcome set
Locked full-cohort sensitivity dataset	Verified 55-participant participant-condition master constructed from the balanced primary dataset and the remaining 25 eligible normal-arch runners using the same outcome-derivation rules	Full-cohort participant-condition dataset (55 participants × 4 conditions = 220 rows)	Participant × condition	Full-cohort sensitivity analyses of the four prespecified discrete families and the complementary Arch Type × Condition LMM	Supplementary sensitivity only; does not replace the balanced n = 30 primary inferential frame

Supplementary Table S2. Metric retrieval index linking manuscript terms, reader-facing analysis labels, source definitions, outputs, and interpretation roles.

Category	Manuscript term / abbreviation	Analysis label	Source definition	Analysis output	Manuscript role
Regional input	Regional peak pressure, PP_r	Regional peak pressure	Peak pressure in T1, T2–5, M1–M5, MF, HM, and HL	Trial-level and participant-condition datasets	Baseline regional-pressure profile and input to MaxPP and MetaPP
Regional input	Regional force impulse, I_r	Regional force impulse	Force-time integral for each functional region	Trial-level and participant-condition datasets	Input to total impulse, MIF, HIF, and MLI
Primary discrete	MaxPP	Maximum regional peak pressure	Maximum regional peak pressure across the 10 functional regions	Single baseline; support-versus-Single; between-height; arch-group change analyses	Primary discrete outcome
Primary discrete	MetaPP	Maximum metatarsal-region peak pressure	Maximum peak pressure across M1–M5	Same analysis families as MaxPP	Primary discrete outcome
Derived denominator	Total impulse	Total regional impulse	Sum of regional impulses across all 10 functional regions	Trial-level and participant-condition datasets	Denominator for MIF and HIF; quality-control variable
Primary discrete	MIF	Midfoot impulse fraction	Midfoot impulse divided by total impulse × 100	Single baseline; support-versus-Single; between-height; arch-group change analyses	Primary discrete outcome
Primary discrete	HIF	Heel impulse fraction	Combined medial- and lateral-heel impulse divided by total impulse × 100	Same analysis families as MIF	Primary discrete outcome
Primary discrete	MLI	mediolateral impulse index	Signed difference between prespecified medial and lateral impulse sets	Same analysis families as MIF	Primary discrete outcome; direction retained
Exploratory composite input	|MLI|	Absolute MLI	Absolute value of MLI	Exploratory application-response summary and candidate score	Used only in the exploratory candidate score
Response metric	Δ	Change from Single	Support-condition value minus Single value	Participant change dataset and arch-group response comparison	Participant response and height-specific arch-group comparison
Visualization descriptor	Group change (%)	Complete-pair group change	Difference between paired group means divided by the reference mean × 100	Heatmaps, trajectories, and candidate-score display	Descriptive figure quantity; not a separate hypothesis test
Dynamic	MFS(t)	Instantaneous midfoot force share	Midfoot force divided by total force at normalized time t × 100	Time-series source workbook and pointwise result tables	Instantaneous midfoot force share
Dynamic	HFS(t)	Instantaneous heel force share	Combined heel force divided by total force at normalized time t × 100	Time-series source workbook and pointwise result tables	Instantaneous heel force share
Dynamic	Dynamic MLI(t)	dynamic mediolateral force index	Signed difference between medial and lateral force sets at normalized time t	Time-series source workbook and pointwise result tables	Instantaneous mediolateral force composition
Secondary CoP	Mediolateral CoP excursion	Mediolateral CoP excursion	Imported from the consolidated CoP metrics workbook	Participant-condition CoP dataset	Auxiliary descriptor; exact upstream definition retained in processing records
Secondary CoP	Anteroposterior CoP excursion	Anteroposterior CoP excursion	Imported from the consolidated CoP metrics workbook	Participant-condition CoP dataset	Auxiliary descriptor
Secondary CoP	CoP path length	CoP path length	Imported from the consolidated CoP metrics workbook	Participant-condition CoP dataset	Auxiliary descriptor
Secondary CoP	CoP mean velocity	CoP mean velocity	Imported from the consolidated CoP metrics workbook	Participant-condition CoP dataset	Auxiliary descriptor
Timing	Stance time	Stance time	Imported timing field or exact-key repair from the first three raw timing trials	Participant-condition timing dataset and pressure-trial quality control	Quality-control and auxiliary timing descriptor
Timing	IMC, IFFC, HO	Initial metatarsal contact; initial forefoot-flat contact; heel-off	Imported timing events expressed as percentage of stance	Participant-condition timing dataset	Auxiliary event timing
Timing	ICP, FFP, POP duration	Initial-contact, forefoot-contact, and propulsive-phase duration	Derived or imported phase durations	Participant-condition timing dataset	Auxiliary phase-duration descriptors

Category	Manuscript term / abbreviation	Analysis label	Source definition	Analysis output	Manuscript role
Regional area	T1–HL regional contact area	Regional contact area	Imported functional-region contact-area fields	Participant-condition regional-area dataset	Auxiliary contact-area descriptors

Supplementary Table S3. Editable equations, units, biomechanical meanings, and interpretation boundaries for the primary discrete metrics.

Abbreviation	Definition and formula	Unit	Biomechanical meaning	Interpretation boundary
PP_r	$PP_r = \max_t P_r(t)$	N/cm²	Instantaneous maximum shoe – ground interface pressure within a predefined functional region.	Not a direct measure of plantar-skin, in-shoe, bone, or soft-tissue stress.
I_r	$I_r = \int_0^T F_r(t)\,dt$	N·s	Time-integrated regional force exposure over stance.	Depends on region mask and contact duration; not equivalent to pressure-time integral.
TotalImpulse	$I_{\text{total}} = \sum_{r\in\mathcal{R}} I_r$ $\mathcal{R} = \{\text{T1,T2-5,M1-M5,MF,HM,HL}\}$	N·s	Total impulse represented by the 10 regional partitions.	Used as a denominator; assumes regional impulses collectively represent the recorded interface load.
MaxPP	$\text{MaxPP} = \max_{r\in\mathcal{R}} PP_r$	N/cm²	Highest region-level pressure peak during a trial.	Region-level maximum, not the maximum individual sensor cell. A lower value is not by itself evidence of lower injury risk.
MetaPP	$\text{MetaPP} = \max_{r\in\{\text{M1},\dots,\text{M5}\}} PP_r$	N/cm²	Highest pressure peak among the five metatarsal functional regions.	Does not represent the mean metatarsal pressure or a single anatomical metatarsal-head stress.
MIF	$\text{MIF} = 100 \times \frac{I_{\text{MF}}}{I_{\text{total}}}$	%	Relative contribution of the midfoot region to total stance-integrated impulse.	An increase indicates greater relative midfoot participation, not necessarily clinical benefit or an increase in absolute midfoot force.
HIF	$\text{HIF} = 100 \times \frac{I_{\text{HM}} + I_{\text{HL}}}{I_{\text{total}}}$	%	Relative contribution of the heel regions to total stance-integrated impulse.	A decrease may reflect redistribution, altered timing, or changes elsewhere; it is not automatically beneficial.
MedialImpulse	$I_{\text{med}} = I_{\text{T1}} + I_{\text{M1}} + I_{\text{M2}} + I_{\text{HM}}$	N·s	Impulse represented by the prespecified medial functional-region set.	Functional, not anatomically exhaustive. M3 and MF are omitted by design.
LateralImpulse	$I_{\text{lat}} = I_{\text{T2-5}} + I_{\text{M4}} + I_{\text{M5}} + I_{\text{HL}}$	N·s	Impulse represented by the prespecified lateral functional-region set.	T2-5 is a combined toe region and is not a purely lateral anatomical compartment.
MLI	$\text{MLI} = 100 \times \frac{I_{\text{med}} - I_{\text{lat}}}{I_{\text{med}} + I_{\text{lat}}}$	%	Signed relative predominance of the prespecified medial versus lateral impulse sets.	Positive values indicate greater medial-set impulse; negative values indicate greater lateral-set impulse. A reduction is not synonymous with normalization or improved balance.
|MLI|	$\lvert\text{MLI}\rvert$	%	Distance of the signed index from zero.	Used only for the exploratory candidate score; zero has not been established as a clinical optimum.
$\Delta_{i,c}$	$\Delta_{i,c} = X_{i,c} - X_{i,\text{Single}}$	Original outcome unit	Participant-specific absolute response to a support condition relative to Single.	When compared between arch groups, tests a height-specific difference in mean response; it is not a global Arch × Condition interaction.
Group change	$\text{Group change} = 100 \times \frac{\bar{X}_c - \bar{X}_{\text{ref}}}{\bar{X}_{\text{ref}}}$	%	Descriptive relative magnitude and direction at group level.	Not the mean of individual ratios and not a separate inferential outcome.

Supplementary Table S4. Editable equations and interpretation boundaries for the exploratory dynamic force-time metrics.

Abbreviation / variable	Formula at normalized time t	Unit	Meaning	Boundary
TotalForce(t)	$F_{\mathrm{total}}(t) = \sum_{r \in \mathcal{R}} F_r(t)$	N	Total recorded regional force at a normalized stance point.	Used as denominator only when positive and finite.
MidfootForce(t)	$F_{\mathrm{MF}}(t)$	N	Instantaneous midfoot-region force.	Functional region at shoe – ground interface.
HeelForce(t)	$F_{\mathrm{heel}}(t) = F_{\mathrm{HM}}(t) + F_{\mathrm{HL}}(t)$	N	Instantaneous combined heel-region force.	Does not equal calcaneal tissue force.
MedialForce(t)	$F_{\mathrm{med}}(t) = F_{\mathrm{T1}}(t) + F_{\mathrm{M1}}(t) + F_{\mathrm{M2}}(t) + F_{\mathrm{HM}}(t)$	N	Instantaneous force in the prespecified medial set.	Functional set; M3 and MF omitted.
LateralForce(t)	$F_{\mathrm{lat}}(t) = F_{\mathrm{T2\text{-}5}}(t) + F_{\mathrm{M4}}(t) + F_{\mathrm{M5}}(t) + F_{\mathrm{HL}}(t)$	N	Instantaneous force in the prespecified lateral set.	T2-5 is combined.
MFS(t)	$\mathrm{MFS}(t) = 100 \times \frac{F_{\mathrm{MF}}(t)}{F_{\mathrm{total}}(t)}$	%	Instantaneous midfoot force share.	Not MIF; MIF is a ratio of integrated impulses.
HFS(t)	$\mathrm{HFS}(t) = 100 \times \frac{F_{\mathrm{heel}}(t)}{F_{\mathrm{total}}(t)}$	%	Instantaneous heel force share.	Not HIF; HIF is a ratio of integrated impulses.
Dynamic MLI(t)	$\mathrm{Dynamic\ MLI}(t) = 100 \times \frac{F_{\mathrm{med}}(t) - F_{\mathrm{lat}}(t)}{F_{\mathrm{med}}(t) + F_{\mathrm{lat}}(t)}$	%	Instantaneous signed mediolateral force composition.	Exploratory; pointwise findings are not SPM clusters or whole-curve effects.
Support-minus-Single curve	$\Delta Y_{i,c}(t) = Y_{i,c}(t) - Y_{i,\mathrm{Single}}(t)$	Metric unit	Temporal profile of within-subject response.	Complete paired subjects only; no imputation.

Supplementary Table S5. Auxiliary CoP, timing, and regional-area metrics and their evidential status.

Metric	Source / computation in current analysis	Unit	Biomechanical role	Use in current manuscript
Mediolateral CoP excursion	Imported from the consolidated CoP workbook; not recomputed by the current pipeline.	mm	Range of mediolateral CoP displacement.	Auxiliary only; verify the exact upstream definition before inferential reporting.
Anteroposterior CoP excursion	Imported from the consolidated CoP workbook.	mm	Range of anteroposterior CoP displacement.	Auxiliary only.
CoP path length	Imported from the consolidated CoP workbook.	mm	Total spatial distance of the CoP trajectory.	Auxiliary only.
CoP mean velocity	Imported from the consolidated CoP workbook.	mm/s	Average CoP path velocity.	Auxiliary only.
Stance time	Imported from the consolidated timing workbook; a missing cell may be filled only from an exact-key match among the first three raw timing trials.	ms	Duration of shoe–ground contact.	Primary trial quality control and auxiliary descriptor.
Initial metatarsal contact	Imported timing event expressed as percentage of stance.	% stance	Timing of initial metatarsal-region contact.	Auxiliary only.
Initial forefoot-flat contact	Imported timing event expressed as percentage of stance.	% stance	Timing of the forefoot-flat event defined by the Footscan export.	Auxiliary only.
Heel-off	Imported timing event expressed as percentage of stance.	% stance	Timing of heel-off.	Auxiliary only.
Initial-contact phase duration	Timing-derived phase duration.	% stance	Relative duration of the early contact phase.	Auxiliary only.
Forefoot-contact phase duration	Timing-derived phase duration.	% stance	Relative duration of the forefoot-flat/contact phase.	Auxiliary only.

Metric	Source / computation in current analysis	Unit	Biomechanical role	Use in current manuscript
Propulsive-phase duration	Timing-derived phase duration.	% stance	Relative duration of propulsion.	Auxiliary only.
Regional contact area	Imported from the consolidated regional-area workbook.	Source unit	Functional-region contact area.	Secondary; do not infer if the trial-grid audit fails.
Raw center-of-force X/Y waveforms	Optional parsing of original center-of-force waveform exports and resampling to 101 points.	mm	Optional center-of-force / CoP waveform audit.	Not required for primary figures; disabled by default.

Supplementary Table S6. Statistical analyses, effect directions, multiplicity families, and inferential roles.

Analysis	Question	Comparison and test	Effect estimate	BH-FDR family	Inferential role
**1. Single baseline**	Do arch groups differ under Single?	Welch independent-samples t test for each of the five primary outcomes	High − Normal mean difference; Hedges' g	5 tests	Direct between-arch comparison under the reference condition
**2. Support vs Single**	Does each selected support condition differ from Single within each arch group?	Paired t tests: 9-24, 12-24, and 15-24 vs Single, separately by arch group and outcome	Support − Single mean difference; Cohen's dz	30 tests	Within-participant condition-related difference relative to Single
**3. Configuration contrasts**	Do the selected support configurations differ within each arch group?	Paired t tests: 12-24 − 9-24; 15-24 − 9-24; 15-24 − 12-24	Paired mean difference; Cohen's dz	30 tests	Configuration-specific within-group contrast
**4. Arch-group response comparison**	At each selected support condition, do arch groups differ in change relative to Single?	Welch test comparing participant-level Δ between High and Normal	High Δ − Normal Δ; Hedges' g	15 tests	Direct condition-specific between-arch response comparison
**5. Arch Type × Condition omnibus LMM**	Does the overall condition-related pattern differ by arch type?	Separate LMM for each outcome: Outcome ~ Arch Type + Condition + Arch Type × Condition + (1 | Participant); ML likelihood-ratio test of interaction	$\chi^2(3)$ for omnibus interaction	5 interaction tests; separate BH-FDR family	Direct omnibus test of Arch Type × Condition
**6. Dynamic pointwise analysis**	At which normalized stance points does support-minus-Single differ from zero?	One-sample t test of participant-level support-minus-Single values at each point	Pointwise mean difference and CI	91 tests separately within each of 18 Arch Type × Condition × metric curves	Exploratory temporal localization
**7. Rank-based robustness analysis**	Are the primary discrete-outcome patterns robust to distributional assumptions?	Wilcoxon signed-rank or Mann–Whitney U test corresponding to the primary contrast	Rank-based sensitivity statistic and p value	General robustness outputs are supplementary; S07-focused support-vs-Single analysis used the corresponding 30-test BH-FDR structure	Robustness assessment alongside primary parametric inference
**8. S07 participant-influence analysis**	Are the principal high-arch findings disproportionately dependent on S07?	Raw-trial audit; rank-based support-vs-Single analysis retaining S07; post hoc leave-S07-out analysis	Reproducibility audit; robustness q; leave-out mean difference/effect estimate	Same 30-test support-vs-Single family for the S07-focused rank-based and leave-out analyses	Participant-level robustness and influence assessment
9. Full-cohort sensitivity analysis	Do the principal arch-specific conclusions depend on selection of 15 of the 40 eligible normal-arch runners?	Repeat the four prespecified discrete analysis families and the identical complementary LMM in all 55 eligible runners (40 Normal, 15 High)	Same discrete effect estimates as the primary framework; 3-df Arch Type × Condition LRT for each LMM outcome	Original BH-FDR families retained within each analysis frame; five omnibus LMM interactions remain a separate five-test family	Sensitivity analysis of sample selection; not a replacement primary analysis

Supplementary Table S7. Quality-control, completeness, and outlier-audit criteria.

Domain	Rule	Pipeline action	Interpretation
Behavioral/technical trial validity	Device non-triggering, obvious turning or step adjustment, targeted plate contact, incomplete foot placement, or clearly incomplete signal.	Excluded before consolidated-data inclusion based on testing records and footprint completeness.	Not automatically detectable from the consolidated numeric table.
Primary grid completeness	Expected 30 × 4 × 2 × 3 trial cells with unique keys.	Pipeline stops if primary cells are missing/duplicated/extra.	Protects against duplicate statistical units and silent missingness.
Total impulse	TotalImpulse > 0.	Primary pipeline stops if violated.	Rejects non-physical/empty force records.
Pressure range	0 ≤ MaxPP ≤ 80 N/cm².	Primary pipeline stops if violated.	Broad plausibility check, not an outcome-based exclusion.
Stance time	100 ≤ stance time ≤ 1,200 ms when available.	Primary pipeline stops if violated.	Broad plausibility check.
Statistical outlier: IQR	Value < Q1 − 1.5×IQR or > Q3 + 1.5×IQR.	Flag only; no automatic removal.	Audit of influence and distribution.
Statistical outlier: modified Z	|0.6745 × (x − median)/MAD| > 3.5.	Flag only; no automatic removal.	Robust audit criterion.
Normality audit	Shapiro-Wilk when 3 ≤ n ≤ 5,000.	Export W and p; primary parametric analysis retained	Diagnostic, not a data-driven test-switching rule.
Timing missingness	Missing consolidated timing value with exact matching raw first-three key and non-missing raw value.	Fill missing cell only; never overwrite existing consolidated value; audit each fill.	Controlled provenance-preserving repair.
Zones label repair	One-to-one missing/extra subject-key pattern with identical Condition × Foot × Trial structure and similar name.	Conservative relabel with audit.	Secondary-table repair only; does not modify primary pressure data.

Supplementary Table S8. Required interpretation boundaries for manuscript consistency.

Observed quantity	Acceptable wording	Wording to avoid
Ground-platform pressure recorded through footwear	Shod shoe – ground interface pressure/load; shod dynamic load.	Direct plantar-skin pressure, in-shoe pressure, tissue stress, or bone load.
MIF increase	Greater relative midfoot impulse contribution.	Improved arch function, beneficial transfer, or reduced injury risk without corroborating outcomes.
HIF decrease	Lower relative heel impulse contribution.	Heel unloading or tissue protection without absolute regional-force evidence.
MLI decrease	Change toward a lower signed mediolateral impulse index.	Improved balance, normalization, or more neutral loading unless a validated optimum is established.
Nonsignificant Δ group comparison	No clear height-specific arch-group response difference was detected in the present sample.	Arch groups respond identically; no moderation; statistical equivalence.
Support vs Single comparison	Observed condition-related difference relative to Single.	An isolated causal effect attributable solely to the support insert or support height.
Pointwise FDR segment	Contiguous sampling points with q < 0.05 in an exploratory pointwise analysis.	SPM cluster, phase-wide effect, or whole-curve significance.
Candidate score	Unvalidated descriptive composite used for visualization.	Validated optimization score or evidence that one height is superior.
Full-cohort sensitivity consistency	The principal arch-specific inference remained consistent when all 40 eligible normal-arch runners were included; report separately that some within-normal-arch contrasts changed with increased precision.	All results were unchanged; the arch groups were equivalent; the LMM proved the original analysis correct.

Supplementary Table S9. Abbreviations used in the manuscript and supplementary material.

Abbreviation	Full term
AS-H9/D24; AS-H12/D24; AS-H15/D24	Arch-support insert with support height of 9, 12, or 15 mm and infill density of 24%
T1	Hallux functional region
T2-5	Second-to-fifth toe functional region

Abbreviation	Full term
M1-M5	First-to-fifth metatarsal functional regions
MF	Midfoot functional region
HM	Medial heel functional region
HL	Lateral heel functional region
MaxPP	Maximum regional peak pressure
MetaPP	Maximum metatarsal-region peak pressure
MIF	Midfoot impulse fraction
HIF	Heel impulse fraction
MLI	mediolateral impulse index
MFS(t)	Instantaneous midfoot force share
HFS(t)	Instantaneous heel force share
Dynamic MLI(t)	dynamic mediolateral force index
CoP	Center of pressure
SPM1D	One-dimensional statistical parametric mapping
BH-FDR	Benjamini-Hochberg false discovery rate
LMM	Linear mixed-effects model
LRT	Likelihood-ratio test
ICC	Intraclass correlation coefficient
LOO	Leave-one-participant-out
AA	Arch angle
CSI	Chippaux–Smirak index

Supplementary Table S10. Operational random-sampling procedure, baseline characteristics, and available static arch-classification descriptors.

Panel A. Operational implementation of balanced random sampling

Step	Normal-arch pool	High-arch pool	Sampling operation	Result
1. Establish the eligible pools	40 eligible runners	15 eligible runners	Arch classification was completed before balanced sampling.	The sampling frame was fixed at 55 eligible runners.
2. Prepare the sampling file	40 unique study identifiers	All 15 retained	The sampling file contained only study identifier and arch classification; primary outcome data were not used.	The normal-arch sampling pool was separated from the outcome dataset.
3. Generate a random order	40 identifiers	—	A computer-generated random sequence placed all 40 normal-arch identifiers in random order.	Each eligible normal-arch runner had the same probability of selection.
4. Select without replacement	First 15 identifiers selected	All 15 included	The first 15 identifiers in the random sequence were retained; no identifier could be selected more than once.	A balanced analytic sample of 15 normal-arch and 15 high-arch runners was formed.
5. Finalize the analytic sample	25 not selected	0 not selected	The selected participant list was fixed before primary-outcome analysis.	Final analytic sample: n = 30; the other 25 normal-arch runners remained eligible but were not analyzed.

Note. The computer-generated sequence determined only the random ordering of the 40 eligible normal-arch study identifiers. The first 15 identifiers were selected without replacement. No demographic or plantar-loading variable was used to choose participants.

Panel B. Baseline comparability of the selected arch groups

Variable	Normal arch (n = 15)	High arch (n = 15)	Mean difference (High - Normal)	95% CI	*p*	Hedges' g
Age, years	33.93 ± 7.39	37.60 ± 7.87	3.67	[-2.05, 9.38]	0.199	0.47
Height, cm	173.27 ± 4.25	175.13 ± 4.03	1.87	[-1.23, 4.97]	0.228	0.44
Body mass, kg	65.37 ± 5.35	68.00 ± 4.57	2.63	[-1.09, 6.36]	0.158	0.52

Note. Values are mean ± standard deviation. Height was converted from meters to centimeters for presentation. Between-group comparisons used two-sided Welch independent-samples t tests. Mean differences and Hedges' g were calculated as High arch minus Normal arch. No values were missing for the reported variables. These analyses were descriptive; a nonsignificant p value was not interpreted as evidence of statistical equivalence.

Panel C. Available AA and CSI distributions in the eligible and balanced analytic cohorts

Analysis frame	Metric	Arch	n	Mean ± SD	Median	IQR	Range
Full 40:15	AA	Normal	40	50.40 ± 3.81°	49.92°	47.84–52.89°	43.45–57.95°
Full 40:15	AA	High	15	53.62 ± 3.71°	53.35°	51.70–55.12°	47.60–60.55°
Full 40:15	CSI	Normal	39	0.313 ± 0.046	0.305	0.285–0.343	0.210–0.420
Full 40:15	CSI	High	9	0.244 ± 0.051	0.225	0.220–0.260	0.190–0.360
Balanced 15:15	AA	Normal	15	50.41 ± 4.77°	49.45°	45.83–54.40°	43.45–57.95°
Balanced 15:15	AA	High	15	53.62 ± 3.71°	53.35°	51.70–55.12°	47.60–60.55°
Balanced 15:15	CSI	Normal	15	0.309 ± 0.045	0.310	0.287–0.338	0.210–0.400
Balanced 15:15	CSI	High	9	0.244 ± 0.051	0.225	0.220–0.260	0.190–0.360

Note. AA, arch angle; CSI, Chippaux–Smirak index. Values are descriptive summaries of the available participant-level descriptors and are reported as mean ± standard deviation, median, interquartile range (IQR), and range. AA and CSI were used as supporting descriptors within the two-stage static morphological assessment and were not applied as independent mandatory numerical cutoff criteria. The balanced 15:15 frame contains the primary analytic sample; the full 40:15 frame contains all 55 eligible runners.

Supplementary Results and Additional Reproducibility Tables

Supplementary Results S11. First-three-valid-trials-per-foot sensitivity analysis

The first-three-valid-trials-per-foot analysis retained complete paired data for 15 participants in each arch group. MFS(t) $q < 0.05$ runs were present for all three support conditions in the normal-arch group and for 12-24 and 15-24 in the high-arch group; high-arch 9-24 remained without $q < 0.05$ points. Dynamic MLI(t) runs were present for all three normal-arch conditions and for high-arch 12-24 and 15-24; high-arch 9-24 remained without $q < 0.05$ points. No HFS(t) curve retained $q < 0.05$ sampling points. Exact runs for all 18 curves and their comparison with the all-valid-trial frame are reported in Supplementary Tables S11 and S12 and Supplementary Figure S1.

Supplementary Table S11. First-three-valid-trials time-series sensitivity analysis.

Arch type	Condition	Dynamic metric	n	q < 0.05 sampling-point runs	No. significant points
Normal	9-24	Midfoot force share	15	16-25%	10
Normal	9-24	Heel force share	15	none	0
Normal	9-24	Dynamic MLI	15	37-73%	37
Normal	12-24	Midfoot force share	15	14-58%	45
Normal	12-24	Heel force share	15	none	0
Normal	12-24	Dynamic MLI	15	14-91%	78
Normal	15-24	Midfoot force share	15	13-50%	38
Normal	15-24	Heel force share	15	none	0
Normal	15-24	Dynamic MLI	15	16-84%	69
High	9-24	Midfoot force share	15	none	0
High	9-24	Heel force share	15	none	0
High	9-24	Dynamic MLI	15	none	0
High	12-24	Midfoot force share	15	27-58%	32
High	12-24	Heel force share	15	none	0
High	12-24	Dynamic MLI	15	38-70%	33
High	15-24	Midfoot force share	15	11-59%	49
High	15-24	Heel force share	15	none	0
High	15-24	Dynamic MLI	15	40-72%; 85-92%	41

Supplementary Figure S1. First-three-valid-trials-per-foot sensitivity analysis of the exploratory stance-phase force-composition results. Participant-level support-minus-Single curves were reconstructed after restricting each participant × condition × foot cell to the first three successfully parsed valid trials. Curves were normalized to 0%–100% stance, and pointwise inference was restricted to 5%–95% stance. Benjamini–Hochberg FDR correction was applied separately to the 91 sampling-point tests within each Arch Type × Condition × temporal-metric curve. The sensitivity analysis was used to evaluate the dependence of the temporal findings on trial sampling and did not replace the primary all-valid-trial analysis. Numerical comparisons between the primary and sensitivity frames are reported in Supplementary Tables S11 and S12.

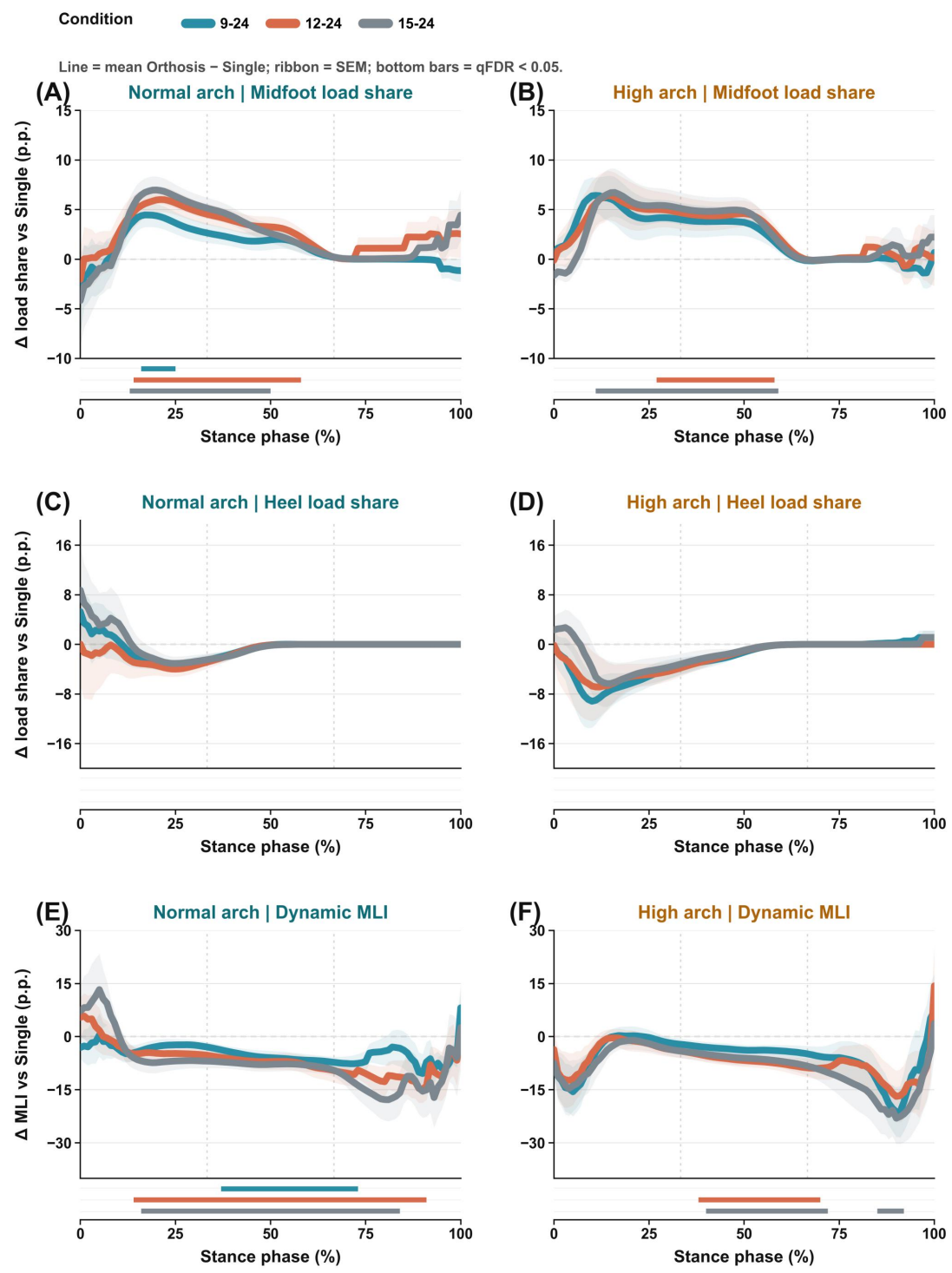

Supplementary Table S12. Comparison of the primary all-valid-trial and first-three-valid-trial time-series analyses.

Arch	Condition	Metric	Primary q < 0.05 runs	First-three q < 0.05 runs	Pearson r	Direction agreement	Mean absolute curve difference (pp)
Normal	9-24	Midfoot	11-68%	16-25%	0.991	0.945	0.553
Normal	9-24	Heel	17-27%	none	0.966	0.802	0.201
Normal	9-24	Dynamic MLI	10-19%; 35-79%; 86-95%	37-73%	0.747	1.0	1.512
Normal	12-24	Midfoot	11-64%	14-58%	0.976	0.989	0.573
Normal	12-24	Heel	21-47%	none	0.985	0.681	0.158
Normal	12-24	Dynamic MLI	16-94%	14-91%	0.959	0.978	0.978
Normal	15-24	Midfoot	11-60%	13-50%	0.979	0.967	0.439
Normal	15-24	Heel	none	none	0.925	0.912	0.257
Normal	15-24	Dynamic MLI	17-95%	16-84%	0.951	1.0	1.552
High	9-24	Midfoot	none	none	0.977	0.813	0.49
High	9-24	Heel	15-24%	none	0.987	0.923	0.322
High	9-24	Dynamic MLI	none	none	0.955	0.989	0.846
High	12-24	Midfoot	12-64%	27-58%	0.972	0.846	0.414
High	12-24	Heel	none	none	0.974	0.978	0.396
High	12-24	Dynamic MLI	31-76%	38-70%	0.97	1.0	0.461
High	15-24	Midfoot	11-62%	11-59%	0.973	0.802	0.543
High	15-24	Heel	none	none	0.981	0.945	0.441
High	15-24	Dynamic MLI	10-11%; 33-73%; 84-95%	40-72%; 85-92%	0.969	1.0	1.263

Note. Sampling-run duration is reported only for temporal location and completeness and is not interpreted as response strength. The full numerical source table is retained in the accompanying supplementary workbook.

Supplementary Table S13. Complementary Arch Type × Condition omnibus linear mixed-effects models for the five primary outcomes.

Outcome	χ^2	df	p	BH-FDR q	Interpretation
MaxPP	0.884	3	0.829	0.835	No statistically significant omnibus interaction after FDR correction.
MetaPP	0.859	3	0.835	0.835	No statistically significant omnibus interaction after FDR correction.
MIF	2.339	3	0.505	0.835	No statistically significant omnibus interaction after FDR correction.
HIF	1.162	3	0.762	0.835	No statistically significant omnibus interaction after FDR correction.
MLI	1.060	3	0.787	0.835	No statistically significant omnibus interaction after FDR correction.

Note. Each outcome was modeled separately using 120 participant-condition observations (30 participants × 4 conditions): Outcome ~ Arch Type + Condition + Arch Type × Condition + (1 | Participant). Models were estimated by maximum likelihood, and the interaction was tested by likelihood-ratio comparison with a reduced model omitting the interaction. The five p values formed a separate BH-FDR family; all five q values were 0.835. Nonsignificance indicates insufficient evidence of an arch-type-dependent overall condition pattern and does not establish equivalence.

Supplementary Table S14. Geometric specifications of the selected arch-support insert models.

Parameter	H9/D24	H12/D24	H15/D24
Nominal support height	9 mm	12 mm	15 mm
Projected longitudinal length	129.85 mm	129.85 mm	129.85 mm
Maximum projected width	58.22 mm	58.22 mm	58.22 mm
Supplementary CAD file	S1	S2	S3
STL filename	Supplementary_CAD_File_S1_H9.stl	Supplementary_CAD_File_S2_H12.stl	Supplementary_CAD_File_S3_H15.stl

Note. Nominal support height refers to the design parameter used to define the three experimental conditions and is not equivalent to the total vertical extent of the STL mesh, which also includes the insert base geometry. For each nominal support height, the left- and right-foot inserts used experimentally were mirror-image counterparts. The three STL models are provided within the accompanying archive Supplementary_CAD_Files_S1-S3.zip as Supplementary CAD Files S1–S3, corresponding to the H9/D24, H12/D24, and H15/D24 configurations, respectively.

Supplementary Figure S2. Three-dimensional surface geometries of the selected arch-support insert models provided as Supplementary CAD Files S1–S3.

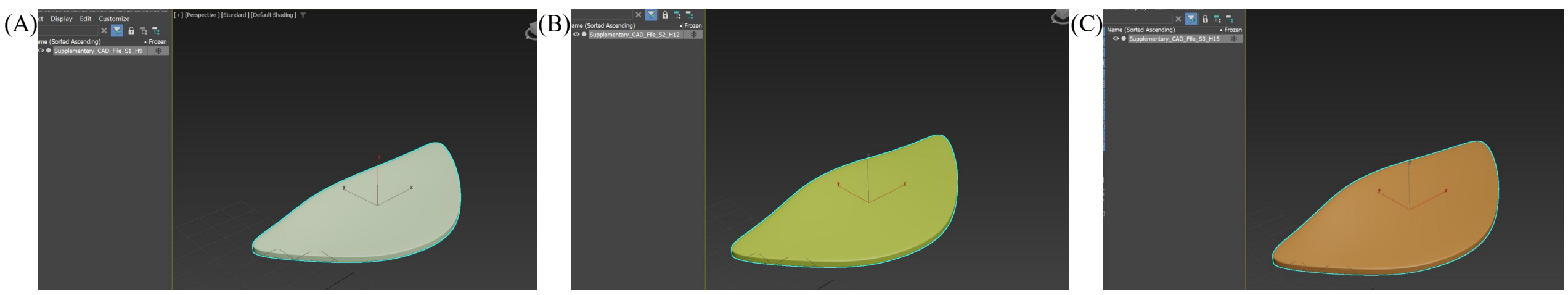

(A) H9/D24, corresponding to Supplementary CAD File S1 (Supplementary_CAD_File_S1_H9.stl); (B) H12/D24, corresponding to Supplementary CAD File S2 (Supplementary_CAD_File_S2_H12.stl); and (C) H15/D24, corresponding to Supplementary CAD File S3 (Supplementary_CAD_File_S3_H15.stl). The three STL files are contained in the accompanying archive Supplementary_CAD_Files_S1-S3.zip. The models share the same projected footprint dimensions and differ in nominal support height. For each nominal support height, the experimentally used contralateral insert was the mirror-image counterpart of the supplied model. Principal geometric specifications are reported in Supplementary Table S14.

Supplementary Table S15. Participant disposition and descriptive comfort/tolerability monitoring.

Panel A. Participants not retained in the complete-protocol sampling frame (n = 14)

Reason	n	Testing stage	Description
Flatfoot classification	1	Screening	Did not meet the target normal/high-arch classification framework.
Habitual forefoot strike	4	Early dynamic testing/protocol verification	Did not meet the intended strike-pattern requirement for consistent acquisition.
Personal scheduling constraints	4	After completing part of the protocol	Personal scheduling prevented completion of the full testing protocol.
Study-shoe fit incompatibility	5	During testing	Inadequate fit in the standardized EUR size 42 study shoe was identified during testing.

Panel B. Descriptive tolerability monitoring among the final 30 completers

Condition	N	Fatigue VAS, median [Q1–Q3], mm	Overall comfort, median [Q1–Q3]	Perceived support, median [Q1–Q3]	Fatigue VAS ≥40 mm, n (%)	Any fatigue >0 mm, n (%)	Condition
9-24	30	0 [0–0]	4 [3–4]	10 [8–11]	0 (0.0%)	3 (10.0%)	9-24
12-24	30	0 [0–0]	3 [2.25–4]	11 [11–13]	0 (0.0%)	4 (13.3%)	12-24
15-24	30	0 [0–10]	3 [2–4]	12 [11–13]	0 (0.0%)	9 (30.0%)	15-24

Panel C. Descriptive tolerability monitoring by arch group

Arch type	Condition	N	Overall comfort, median [Q1–Q3]	Perceived support, median [Q1–Q3]	Recommendation ≥4 stars, n (%)	Arch type
High	9-24	15	3 [3–4]	10 [8–11]	5 (33.3%)	High
High	12-24	15	4 [3–4]	11 [8.5–11.5]	9 (60.0%)	High
High	15-24	15	4 [3–4]	12 [11–13]	6 (40.0%)	High
Normal	9-24	15	4 [3–4]	9 [8–10.5]	6 (40.0%)	Normal
Normal	12-24	15	3 [2–3]	13 [11–13.5]	1 (6.7%)	Normal
Normal	15-24	15	3 [2–4]	12 [12–13.5]	4 (26.7%)	Normal

Note. The exact experimental condition at discontinuation was not systematically retained for participants who did not complete the full protocol and was therefore not retrospectively assigned. No protocol discontinuation was documented as fatigue-related or as being caused by an adverse response attributable to a specific 9-, 12-, or 15-mm support condition. Study-shoe fit incompatibility should not be interpreted as arch-support-related discomfort. Panels B and C refer only to the final 30 participants who completed the analyzed conditions and are descriptive rather than inferential. Overall comfort was assessed using the original 5-point scale, perceived support using the original 15-point scale, and fatigue using a 100-mm visual analogue scale (VAS).^15 Values previously expressed on the equivalent 0–10 cm VAS are reported here in millimetres for consistency with the original protocol. Recorded discomfort or fatigue among completers should not be interpreted as a reason for protocol discontinuation. Conversely, the absence of a documented support-height-specific discontinuation does not imply that no participant reported discomfort. No subjective–objective correlation or between-height superiority test was performed.

Supplementary Table S16. Independent raw-export audit and influence analysis for participant S07.

Panel A. File-integrity, numerical-quality, and first-three-trial reconstruction audit

Condition	Primary retained trials	Export-verifiable numerical QC	Raw reconstruction and MIF reproducibility	Primary-analysis disposition
Single	6 (3 left + 3 right)	6/6 passed	MIF = 4.265%; reconstructed across both feet and repeated trials	Retained
9-24	6 (3 left + 3 right)	6/6 passed	MIF = 22.802%; all 6 order-matched trial differences were higher than Single	Retained
12-24	6 (3 left + 3 right)	6/6 passed	MIF = 22.939%; all 6 order-matched trial differences were higher than Single	Retained
15-24	6 (3 left + 3 right)	6/6 passed	MIF = 22.441%; all 6 order-matched trial differences were higher than Single	Retained
Total	24	24/24 passed	All five primary S07 values reproduced to numerical precision; 18/18 support-versus-Single MIF trial differences were positive	S07 retained in the primary n = 15 analysis

Panel B. Rank-based robustness analysis retaining S07 in the full high-arch sample (n = 15)

Outcome	9-24 vs Single, BH-FDR q	12-24 vs Single, BH-FDR q	15-24 vs Single, BH-FDR q	Pattern
**MIF**	**0.0012**	**0.0014**	**0.0012**	FDR-significant under all three conditions
**MLI**	**0.0339**	**0.0183**	**0.0183**	FDR-significant under all three conditions
HIF	0.0193	0.0451	0.1766	Significant for 9-24 and 12-24; not significant for 15-24

Panel C. Post hoc leave-S07-out influence analysis of the high-arch support-versus-Single results

Outcome	Contrast	Leave-S07-out mean difference	BH-FDR q	Influence result	Outcome
MIF	9-24 − Single	+2.49 pp	0.00049	Principal MIF pattern retained	MIF
MIF	12-24 − Single	+3.17 pp	0.00047	Principal MIF pattern retained	MIF
MIF	15-24 − Single	+3.56 pp	<0.00001	Principal MIF	MIF

Outcome	Contrast	Leave-S07-out mean difference	BH-FDR q	Influence result	Outcome
				pattern retained	
MLI	9-24 − Single	−3.81 pp	0.0138	Principal MLI pattern retained	MLI
MLI	12-24 − Single	−6.05 pp	0.0077	Principal MLI pattern retained	MLI
MLI	15-24 − Single	−6.10 pp	0.0077	Principal MLI pattern retained	MLI

Note. The 9-24 HIF comparison was more influence-sensitive: its BH-FDR q value changed from 0.044 in the primary n = 15 analysis to 0.070 in the leave-S07-out analysis. MaxPP and MetaPP did not become FDR significant after S07 exclusion.

Panel D. Broader leave-S07-out influence checks

Analysis family	Result after exclusion of S07	Interpretation
High-arch direct configuration contrasts	All BH-FDR q ≥ 0.114	The configuration-contrast inference was retained
Between-arch comparisons of participant-level Δ	All BH-FDR q ≥ 0.809	The between-arch Δ inference was retained
MaxPP and MetaPP support-versus-Single comparisons	No FDR-significant result emerged after S07 exclusion	Their inferential status was unchanged

Note. The four standalone S07 raw exports were byte-identical to their corresponding copies in the 30-participant archive. The files contained 22, 23, 22, and 22 parseable footfalls under Single, 9-24, 12-24, and 15-24, respectively. Following the primary statistical-unit rule, the first three trials from each foot were selected (24 trials total). All 24 had finite force and pressure signals, positive total impulse, MaxPP within 0–80 N/cm², and export-derived stance durations of 265–435 ms. Reconstruction from these raw curves matched the consolidated MaxPP, MetaPP, MIF, HIF, and MLI values to numerical precision (maximum absolute discrepancy 1.1 × 10^{-14}). All 18 order-matched support-versus-Single MIF differences were positive across three conditions, two feet, and three repeated trials. The numerical exports do not independently encode behavioral criteria such as deliberate plate targeting or stride alteration; those criteria remained governed by the contemporaneous testing records used for original trial retention. No export-level technical exclusion criterion was identified. Panels B–D report the rank-based and leave-S07-out analyses; the full n = 15 high-arch sample remained primary.

Supplementary Table S17. Complete Single baseline and support-versus-Single results for the five primary outcomes.

Panel A. Between-arch comparisons under Single (High − Normal)

Outcome	Unit	Mean difference [95% CI]	p	BH-FDR q	Hedges' g
MaxPP	N/cm²	-0.11 [-1.49, +1.27]	0.875	0.875	-0.06
MetaPP	N/cm²	-0.31 [-1.61, +0.99]	0.626	0.783	-0.17
MIF	percentage points	-1.25 [-4.72, +2.23]	0.467	0.779	-0.26
HIF	percentage points	+4.14 [+0.71, +7.57]	0.020	0.101	+0.89
MLI	percentage points	-2.45 [-8.43, +3.53]	0.409	0.779	-0.30

Panel B. Within-arch support-versus-Single paired comparisons (support − Single)

Arch	Contrast	Outcome	Mean difference [95% CI]	Group-mean Δ%	p	BH-FDR q	Cohen's dz
Normal	9-24 − Single	MaxPP	-0.28 [-1.10, +0.54]	-3.3	0.478	0.482	-0.19
Normal	12-24 − Single	MaxPP	-0.72 [-1.42, -0.02]	-8.5	0.044	0.073	-0.57
Normal	15-24 − Single	MaxPP	-0.74 [-1.58, +0.10]	-8.7	0.081	0.117	-0.49
Normal	9-24 − Single	MetaPP	-0.27 [-1.08, +0.54]	-3.2	0.482	0.482	-0.19
Normal	12-24 − Single	MetaPP	-0.71 [-1.40, -0.03]	-8.4	0.042	0.073	-0.58
Normal	15-24 − Single	MetaPP	-0.76 [-1.59, +0.08]	-9.0	0.072	0.113	-0.50
Normal	9-24 − Single	MIF	+2.04 [+0.17, +3.91]	+9.7	0.035	0.066	+0.60
Normal	12-24 − Single	MIF	+3.75 [+1.89, +5.61]	+17.9	<0.001	0.005	+1.11
Normal	15-24 − Single	MIF	+4.10 [+2.39, +5.82]	+19.5	<0.001	0.005	+1.33
Normal	9-24 − Single	HIF	-1.75 [-2.95, -0.55]	-14.4	0.007	0.020	-0.81
Normal	12-24 − Single	HIF	-1.82 [-3.49, -0.15]	-14.9	0.035	0.066	-0.60
Normal	15-24 − Single	HIF	-1.13 [-2.60, +0.34]	-9.3	0.123	0.160	-0.42
Normal	9-24 − Single	MLI	-4.26 [-6.96, -1.56]	-14.0	0.004	0.015	-0.88
Normal	12-24 − Single	MLI	-6.13 [-9.68, -2.58]	-20.1	0.002	0.012	-0.96
Normal	15-24 − Single	MLI	-7.34 [-10.65, -4.03]	-24.0	<0.001	0.005	-1.23
High	9-24 − Single	MaxPP	-0.56 [-1.52, +0.41]	-6.6	0.235	0.282	-0.32
High	12-24 − Single	MaxPP	-0.58 [-1.76, +0.61]	-6.9	0.313	0.361	-0.27
High	15-24 − Single	MaxPP	-0.79 [-1.69, +0.11]	-9.4	0.082	0.117	-0.48
High	9-24 − Single	MetaPP	-0.36 [-1.32, +0.61]	-4.3	0.444	0.482	-0.20
High	12-24 − Single	MetaPP	-0.41 [-1.56, +0.74]	-5.0	0.461	0.482	-0.20
High	15-24 − Single	MetaPP	-0.61 [-1.53, +0.31]	-7.5	0.176	0.220	-0.37
High	9-24 − Single	MIF	+3.56 [+1.12, +6.00]	+18.0	0.007	0.020	+0.81
High	12-24 − Single	MIF	+4.20 [+1.76, +6.64]	+21.3	0.002	0.012	+0.95
High	15-24 − Single	MIF	+4.53 [+2.33, +6.74]	+23.0	<0.001	0.005	+1.14
High	9-24 − Single	HIF	-2.62 [-4.74, -0.50]	-16.0	0.019	0.044	-0.68
High	12-24 − Single	HIF	-2.54 [-4.73, -0.35]	-15.5	0.026	0.056	-0.64
High	15-24 − Single	HIF	-2.18 [-4.89, +0.53]	-13.4	0.106	0.144	-0.45
High	9-24 − Single	MLI	-3.37 [-5.82, -0.93]	-12.0	0.010	0.026	-0.76
High	12-24 − Single	MLI	-5.41 [-8.81, -2.00]	-19.2	0.004	0.015	-0.88
High	15-24 − Single	MLI	-5.52 [-8.92, -2.12]	-19.7	0.004	0.015	-0.90

Note. MIF, HIF, and MLI differences are in percentage points; MaxPP and MetaPP differences are in N/cm². Group-mean Δ% was calculated as 100 × (support − Single)/Single. BH-FDR correction was applied across the 30 support-versus-Single tests in Panel B.

Supplementary Table S18. Complete within-arch comparisons among selected support configurations.

Arch	Contrast	Outcome	Mean difference [95% CI]	Group-mean Δ%	p	BH-FDR q	Cohen's dz
Normal	12-24 − 9-24	MaxPP	-0.44 [-0.83, -0.05]	-5.4	0.030	0.139	-0.63
Normal	15-24 − 9-24	MaxPP	-0.46 [-0.88, -0.04]	-5.6	0.034	0.139	-0.61
Normal	15-24 − 12-24	MaxPP	-0.02 [-0.57, +0.53]	-0.3	0.940	0.940	-0.02
Normal	12-24 − 9-24	MetaPP	-0.44 [-0.83, -0.05]	-5.4	0.030	0.139	-0.63
Normal	15-24 − 9-24	MetaPP	-0.49 [-0.93, -0.05]	-5.9	0.033	0.139	-0.61
Normal	15-24 − 12-24	MetaPP	-0.04 [-0.61, +0.52]	-0.6	0.868	0.940	-0.04
Normal	12-24 − 9-24	MIF	+1.71 [+0.70, +2.73]	+7.4	0.003	0.041	+0.94
Normal	15-24 − 9-24	MIF	+2.07 [+1.24, +2.89]	+9.0	<0.001	0.003	+1.39
Normal	15-24 − 12-24	MIF	+0.35 [-0.50, +1.21]	+1.4	0.388	0.582	+0.23
Normal	12-24 − 9-24	HIF	-0.07 [-1.23, +1.09]	-0.6	0.903	0.940	-0.03
Normal	15-24 − 9-24	HIF	+0.63 [-0.40, +1.65]	+6.0	0.212	0.398	+0.34
Normal	15-24 − 12-24	HIF	+0.69 [-0.33, +1.72]	+6.7	0.170	0.356	+0.37
Normal	12-24 − 9-24	MLI	-1.87 [-4.10, +0.35]	-7.1	0.092	0.226	-0.47
Normal	15-24 − 9-24	MLI	-3.08 [-5.24, -0.92]	-11.7	0.008	0.085	-0.79
Normal	15-24 − 12-24	MLI	-1.21 [-4.30, +1.88]	-5.0	0.416	0.594	-0.22
High	12-24 − 9-24	MaxPP	-0.02 [-0.48, +0.44]	-0.3	0.923	0.940	-0.03
High	15-24 − 9-24	MaxPP	-0.23 [-0.47, +0.01]	-3.0	0.057	0.171	-0.54
High	15-24 − 12-24	MaxPP	-0.21 [-0.67, +0.25]	-2.7	0.339	0.573	-0.26
High	12-24 − 9-24	MetaPP	-0.05 [-0.51, +0.41]	-0.7	0.815	0.940	-0.06
High	15-24 − 9-24	MetaPP	-0.26 [-0.50, -0.02]	-3.3	0.037	0.139	-0.59
High	15-24 − 12-24	MetaPP	-0.21 [-0.67, +0.26]	-2.6	0.358	0.573	-0.25
High	12-24 − 9-24	MIF	+0.64 [-0.33, +1.61]	+2.8	0.178	0.356	+0.37
High	15-24 − 9-24	MIF	+0.97 [+0.00, +1.94]	+4.2	0.049	0.165	+0.56
High	15-24 − 12-24	MIF	+0.33 [-0.42, +1.08]	+1.4	0.363	0.573	+0.24
High	12-24 − 9-24	HIF	+0.08 [-1.27, +1.44]	+0.6	0.898	0.940	+0.03
High	15-24 − 9-24	HIF	+0.44 [-1.02, +1.90]	+3.2	0.531	0.725	+0.17
High	15-24 − 12-24	HIF	+0.35 [-0.96, +1.67]	+2.6	0.572	0.747	+0.15
High	12-24 − 9-24	MLI	-2.03 [-4.49, +0.43]	-8.2	0.098	0.226	-0.46
High	15-24 − 9-24	MLI	-2.15 [-4.47, +0.17]	-8.7	0.067	0.182	-0.51
High	15-24 − 12-24	MLI	-0.12 [-1.69, +1.46]	-0.5	0.875	0.940	-0.04

Note. Contrast directions are 12-24 − 9-24, 15-24 − 9-24, and 15-24 − 12-24. MIF, HIF, and MLI differences are in percentage points; MaxPP and MetaPP differences are in N/cm². Group-mean Δ% uses the second-listed condition as the denominator reference. BH-FDR correction was applied across all 30 configuration contrasts.

Supplementary Table S19. Complete between-arch comparisons of participant-level condition-related changes.

Condition	Outcome	High Δ − Normal Δ [95% CI]	p	BH-FDR q	Hedges' g
9-24	MaxPP	-0.28 [-1.49, +0.93]	0.640	0.934	-0.17
9-24	MetaPP	-0.08 [-1.29, +1.12]	0.889	0.934	-0.05
9-24	MIF	+1.52 [-1.42, +4.47]	0.298	0.934	+0.38
9-24	HIF	-0.87 [-3.22, +1.49]	0.454	0.934	-0.27
9-24	MLI	+0.89 [-2.59, +4.37]	0.606	0.934	+0.19
12-24	MaxPP	+0.14 [-1.18, +1.47]	0.826	0.934	+0.08
12-24	MetaPP	+0.31 [-0.98, +1.60]	0.626	0.934	+0.18
12-24	MIF	+0.45 [-2.49, +3.39]	0.754	0.934	+0.11
12-24	HIF	-0.72 [-3.36, +1.93]	0.582	0.934	-0.20
12-24	MLI	+0.73 [-3.97, +5.42]	0.753	0.934	+0.11
15-24	MaxPP	-0.05 [-1.23, +1.13]	0.934	0.934	-0.03
15-24	MetaPP	+0.15 [-1.04, +1.34]	0.801	0.934	+0.09
15-24	MIF	+0.43 [-2.24, +3.10]	0.744	0.934	+0.12
15-24	HIF	-1.05 [-4.04, +1.93]	0.471	0.934	-0.26
15-24	MLI	+1.82 [-2.71, +6.35]	0.418	0.934	+0.29

Note. Participant-level Δ = support condition − Single; between-arch differences are High Δ − Normal Δ. MIF, HIF, and MLI differences are in percentage points; MaxPP and MetaPP differences are in N/cm². BH-FDR correction was applied across the 15 tests. Nonsignificant comparisons do not establish equivalence.

Supplementary Table S20. Conditional Monte Carlo design sensitivity for the five Arch Type × Condition omnibus tests.

Panel A. Eighty-percent-power minimum detectable interaction at nominal $\alpha = 0.05$

Outcome	Unit	Residual SD	Common shift	Linear divergence	Localized difference
MaxPP	N/cm²	0.880	1.23	1.43	1.23
MetaPP	N/cm²	0.878	1.22	1.42	1.22
MIF	percentage points	2.000	2.78	3.24	2.78
HIF	percentage points	2.020	2.81	3.27	2.81
MLI	percentage points	3.435	4.78	5.56	4.78

Panel B. Conservative five-test benchmark ($p \leq 0.01$, sufficient for BH $q \leq 0.05$ regardless of the other four p values)

Outcome	Unit	Common shift	Linear divergence	Localized difference
MaxPP	N/cm²	1.46	1.70	1.46
MetaPP	N/cm²	1.45	1.69	1.45
MIF	percentage points	3.31	3.86	3.31
HIF	percentage points	3.35	3.89	3.35
MLI	percentage points	5.69	6.62	5.69

Note. The interaction vector was defined as the between-arch difference (High − Normal) in each support-minus-Single change for 9-24, 12-24, and 15-24. The three declared shapes were (δ, δ, δ), (δ/3, 2δ/3, δ), and (0, 0, δ), respectively; δ is therefore the largest absolute interaction coefficient. Simulations used the outcome-specific full-model residual variance, 15 participants per arch group, the complete four-condition design, 200,000 Monte Carlo replicates, seed 20260824, and the 3-df χ^2 reference used for the omnibus test. At the reported thresholds, simulated power was 0.800 (Monte Carlo 95% CI 0.798–0.802). Because the BH rejection threshold depends on all five p values, Panel B is a conservative sufficient benchmark rather than an exact BH power calculation. These design-sensitivity estimates do not constitute observed power, an all-55 reanalysis, or evidence of equivalence.

Supplementary Results S12. Full-cohort sensitivity analysis

Full-cohort sensitivity of the prespecified discrete analyses

The Single baseline family remained 0/5 FDR-supported in the full cohort. The number of FDR-supported support-versus-Single comparisons increased from 13/30 in the balanced analysis to 19/30 in the full-cohort analysis, and FDR-supported direct configuration contrasts increased from 2/30 to 4/30. These changes were driven principally by the expanded normal-arch sample and the associated gain in precision. Because BH-FDR q values were recalculated across the complete multiplicity family, changes in FDR status should not be attributed exclusively to the normal-arch observations. Most importantly for the arch-specific response question, the between-arch High Δ − Normal Δ family remained 0/15 FDR-supported, and all 15 corresponding full-cohort 95% confidence intervals included zero. Thus, the full cohort did not generate FDR-supported height-specific between-arch differences in support-related change.

The between-arch change-score conclusion was also stable under participant-level influence and sampling diagnostics: no leave-one-participant-out recomputation produced an FDR-supported between-arch Δ comparison, and none of 10,000 repeated Normal15 draws produced an FDR-supported between-arch Δ comparison. Rank-based checks agreed with the parametric significance status for all 15 between-arch Δ comparisons.

Sensitivity of the Arch Type × Condition interaction

The balanced 15:15 LMM analysis yielded 0/5 FDR-supported Arch Type × Condition omnibus interactions. Repeating the identical model specification in the full 40:15 cohort likewise yielded 0/5 FDR-supported interactions: MaxPP, $\chi^2(3) = 1.746$, $p = 0.627$, $q = 0.990$; MetaPP, $\chi^2(3) = 0.907$, $p = 0.824$, $q = 0.990$; MIF, $\chi^2(3) = 4.059$, $p = 0.255$, $q = 0.990$; HIF, $\chi^2(3) = 2.918$, $p = 0.405$, $q = 0.990$; and MLI, $\chi^2(3) = 0.116$, $p = 0.990$, $q = 0.990$. The omnibus Arch Type × Condition inference therefore remained consistent when all eligible normal-arch runners were included.

The interaction estimates were not numerically identical across sample frames, and several became more precise after inclusion of the additional normal-arch participants. One isolated full-cohort model-based MIF contrast at 9-24 was +1.502 percentage points (95% Wald CI 0.007 to 2.998; unadjusted $p = 0.049$). This coefficient did not remain significant after the descriptive BH adjustment across the 15 interaction coefficients ($q = 0.660$), while the corresponding 3-df MIF omnibus interaction remained nonsignificant ($p = 0.255$; $q = 0.990$). The prespecified Welch change-score comparison had the same point estimate (+1.502 percentage points) but a wider 95% CI (-1.059 to 4.064), $p = 0.234$, and $q = 0.902$. This isolated nominal Wald coefficient was therefore not interpreted as evidence of a systematic arch-type-specific response.

Model diagnostics and robustness

All balanced and full-cohort full/reduced LMMs converged, and all estimated random-intercept variances were positive. Full-cohort ICCs ranged from 0.597 to 0.820, consistent with substantial within-participant correlation across the four repeated conditions. Conditional residual diagnostics nevertheless showed departures from Gaussian normality for several outcomes, particularly MaxPP, MetaPP, and MIF. Targeted omission of the participant associated with the largest standardized residual did not produce a significant omnibus interaction for any outcome. Across the complete full-cohort LMM leave-one-participant-out analysis (55 omissions per outcome), no omnibus interaction p value fell below 0.05.

Taken together, the full-cohort discrete sensitivity analyses and the complementary omnibus LMM provide concordant evidence at the level of the principal arch-specific inference: inclusion of all 40 eligible normal-arch runners did not create FDR-supported evidence that static arch type systematically modified the height-specific changes or the overall condition-related loading-response pattern. This statement does not imply statistical equivalence of the two arch groups.

Complete numerical results for the full-cohort discrete sensitivity analyses, complementary LMMs, model-based contrasts, diagnostics, and influence analyses are reported in Supplementary Tables S21–S30.

Supplementary Table S21. Participant-level descriptive statistics in the balanced and full-cohort analysis frames.

Frame	Arch	Condition	MaxPP	MetaPP	MIF	HIF	MLI
Balanced 15:15	Normal	Single	8.48 ± 1.53	8.48 ± 1.51	21.00 ± 3.98	12.19 ± 3.32	30.54 ± 7.79
Balanced 15:15	Normal	9-24	8.20 ± 0.71	8.20 ± 0.71	23.04 ± 2.80	10.44 ± 3.10	26.28 ± 6.57
Balanced 15:15	Normal	12-24	7.76 ± 0.83	7.76 ± 0.83	24.75 ± 3.17	10.37 ± 3.71	24.40 ± 8.21
Balanced 15:15	Normal	15-24	7.74 ± 0.79	7.72 ± 0.81	25.10 ± 2.36	11.06 ± 2.78	23.19 ± 5.34
Balanced 15:15	High	Single	8.38 ± 2.10	8.17 ± 1.93	19.75 ± 5.20	16.33 ± 5.50	28.09 ± 8.19
Balanced 15:15	High	9-24	7.82 ± 0.81	7.81 ± 0.82	23.31 ± 2.16	13.71 ± 4.28	24.71 ± 8.80
Balanced 15:15	High	12-24	7.80 ± 0.80	7.76 ± 0.84	23.96 ± 2.55	13.79 ± 4.51	22.68 ± 7.78
Balanced 15:15	High	15-24	7.59 ± 0.90	7.55 ± 0.94	24.29 ± 2.93	14.15 ± 4.39	22.56 ± 7.47
Full 40:15	Normal	Single	8.56 ± 1.50	8.54 ± 1.51	21.33 ± 4.33	12.08 ± 4.41	28.47 ± 9.60
Full 40:15	Normal	9-24	8.29 ± 1.38	8.28 ± 1.39	23.39 ± 4.16	10.72 ± 3.97	24.76 ± 9.05
Full 40:15	Normal	12-24	8.10 ± 1.46	8.09 ± 1.47	24.60 ± 3.77	10.35 ± 3.85	22.69 ± 9.32
Full 40:15	Normal	15-24	8.23 ± 1.44	8.21 ± 1.46	24.81 ± 3.77	10.46 ± 3.74	22.43 ± 7.94
Full 40:15	High	Single	8.38 ± 2.10	8.17 ± 1.93	19.75 ± 5.20	16.33 ± 5.50	28.09 ± 8.19
Full 40:15	High	9-24	7.82 ± 0.81	7.81 ± 0.82	23.31 ± 2.16	13.71 ± 4.28	24.71 ± 8.80
Full 40:15	High	12-24	7.80 ± 0.80	7.76 ± 0.84	23.96 ± 2.55	13.79 ± 4.51	22.68 ± 7.78
Full 40:15	High	15-24	7.59 ± 0.90	7.55 ± 0.94	24.29 ± 2.93	14.15 ± 4.39	22.56 ± 7.47

Note. Values are participant-level mean ± SD. Balanced = 15 normal-arch + 15 high-arch runners; Full = 40 normal-arch + 15 high-arch runners. High-arch values are unchanged between frames because all 15 eligible high-arch runners were included in both analyses.

Supplementary Table S22. Complete sensitivity analysis of the prespecified discrete-outcome families: balanced 15:15 versus full 40:15.

Panel A. Single baseline comparisons (High − Normal).

Outcome	Balanced estimate (95% CI)	Balanced p	Balanced q	Balanced g	Full estimate (95% CI)	Full p	Full q	Full g
MaxPP	-0.107 (-1.487 to +1.274)	0.875	0.875	-0.056	-0.180 (-1.418 to +1.057)	0.764	0.883	-0.106
MetaPP	-0.311 (-1.609 to +0.987)	0.626	0.783	-0.175	-0.371 (-1.520 to +0.777)	0.509	0.848	-0.224
MIF	-1.246 (-4.718 to +2.225)	0.467	0.779	-0.262	-1.579 (-4.705 to +1.547)	0.306	0.765	-0.340
HIF	+4.140 (+0.708 to +7.572)	0.020	0.101	+0.887	+4.248 (+0.959 to +7.538)	0.014	0.069	+0.887
MLI	-2.449 (-8.430 to +3.532)	0.409	0.779	-0.298	-0.387 (-5.710 to +4.936)	0.883	0.883	-0.041

Panel B. Support-versus-Single comparisons within each arch group (support − Single).

Arch	Condition	Outcome	Balanced estimate (95% CI)	Balanced p	Balanced q	Balanced dz	Full estimate (95% CI)	Full p	Full q	Full dz
Normal	9-24	MaxPP	-0.278 (-1.097 to +0.541)	0.478	0.482	-0.188	-0.263 (-0.704 to +0.179)	0.236	0.272	-0.190
Normal	9-24	MetaPP	-0.272 (-1.081 to +0.536)	0.482	0.482	-0.187	-0.254 (-0.695 to +0.187)	0.251	0.279	-0.184
Normal	9-24	MIF	+2.037 (+0.168 to +3.906)	0.035	0.066	+0.604	+2.059 (+1.168 to +2.949)	<0.001	<0.001	+0.739
Normal	9-24	HIF	-1.753 (-2.954 to -0.553)	0.007	0.020	-0.809	-1.363 (-2.036 to -0.690)	<0.001	<0.001	-0.648
Normal	9-24	MLI	-4.260 (-6.956 to -1.565)	0.004	0.015	-0.875	-3.718 (-5.858 to -1.577)	0.001	0.003	-0.555

Arch	Condition	Outcome	Balanced estimate (95% CI)	Balanced p	Balanced q	Balanced dz	Full estimate (95% CI)	Full p	Full q	Full dz
Normal	12-24	MaxPP	-0.720 (-1.416 to -0.024)	0.044	0.073	-0.573	-0.453 (-0.842 to -0.064)	0.024	0.042	-0.372
Normal	12-24	MetaPP	-0.714 (-1.399 to -0.029)	0.042	0.073	-0.578	-0.445 (-0.835 to -0.055)	0.026	0.042	-0.365
Normal	12-24	MIF	+3.750 (+1.887 to +5.613)	<0.001	0.005	+1.115	+3.268 (+2.378 to +4.158)	<0.001	<0.001	+1.174
Normal	12-24	HIF	-1.820 (-3.495 to -0.145)	0.035	0.066	-0.602	-1.726 (-2.546 to -0.907)	<0.001	<0.001	-0.674
Normal	12-24	MLI	-6.134 (-9.685 to -2.584)	0.002	0.012	-0.957	-5.783 (-7.776 to -3.789)	<0.001	<0.001	-0.928
Normal	15-24	MaxPP	-0.740 (-1.582 to +0.103)	0.081	0.117	-0.486	-0.325 (-0.793 to +0.142)	0.167	0.219	-0.223
Normal	15-24	MetaPP	-0.759 (-1.595 to +0.077)	0.072	0.113	-0.503	-0.325 (-0.792 to +0.142)	0.168	0.219	-0.222
Normal	15-24	MIF	+4.105 (+2.392 to +5.817)	<0.001	0.005	+1.327	+3.476 (+2.714 to +4.239)	<0.001	<0.001	+1.458
Normal	15-24	HIF	-1.128 (-2.600 to +0.344)	0.123	0.160	-0.424	-1.616 (-2.377 to -0.855)	<0.001	<0.001	-0.679
Normal	15-24	MLI	-7.343 (-10.655 to -4.031)	<0.001	0.005	-1.228	-6.046 (-8.054 to -4.039)	<0.001	<0.001	-0.963
High	9-24	MaxPP	-0.557 (-1.519 to +0.405)	0.235	0.282	-0.321	-0.557 (-1.519 to +0.405)	0.235	0.272	-0.321
High	9-24	MetaPP	-0.355 (-1.323 to +0.612)	0.444	0.482	-0.203	-0.355 (-1.323 to +0.612)	0.444	0.460	-0.203
High	9-24	MIF	+3.561 (+1.119 to +6.003)	0.007	0.020	+0.807	+3.561 (+1.119 to +6.003)	0.007	0.016	+0.807
High	9-24	HIF	-2.619 (-4.739 to -0.499)	0.019	0.044	-0.684	-2.619 (-4.739 to -0.499)	0.019	0.036	-0.684
High	9-24	MLI	-3.375 (-5.824 to -0.926)	0.010	0.026	-0.763	-3.375 (-5.824 to -0.926)	0.010	0.021	-0.763
High	12-24	MaxPP	-0.578 (-1.762 to +0.607)	0.313	0.361	-0.270	-0.578 (-1.762 to +0.607)	0.313	0.336	-0.270
High	12-24	MetaPP	-0.406 (-1.557 to +0.744)	0.461	0.482	-0.196	-0.406 (-1.557 to +0.744)	0.461	0.461	-0.196
High	12-24	MIF	+4.203 (+1.764 to +6.642)	0.002	0.012	+0.954	+4.203 (+1.764 to +6.642)	0.002	0.007	+0.954
High	12-24	HIF	-2.537 (-4.728 to -0.345)	0.026	0.056	-0.641	-2.537 (-4.728 to -0.345)	0.026	0.042	-0.641
High	12-24	MLI	-5.406 (-8.808 to -2.005)	0.004	0.015	-0.880	-5.406 (-8.808 to -2.005)	0.004	0.010	-0.880
High	15-24	MaxPP	-0.788 (-1.690 to +0.114)	0.082	0.117	-0.484	-0.788 (-1.690 to +0.114)	0.082	0.123	-0.484
High	15-24	MetaPP	-0.612 (-1.532 to +0.309)	0.176	0.220	-0.368	-0.612 (-1.532 to +0.309)	0.176	0.220	-0.368
High	15-24	MIF	+4.533 (+2.330 to +6.737)	<0.001	0.005	+1.139	+4.533 (+2.330 to +6.737)	<0.001	0.002	+1.139
High	15-24	HIF	-2.182 (-4.889 to +0.525)	0.106	0.144	-0.446	-2.182 (-4.889 to +0.525)	0.106	0.151	-0.446
High	15-24	MLI	-5.524 (-8.924 to -2.124)	0.004	0.015	-0.900	-5.524 (-8.924 to -2.124)	0.004	0.009	-0.900

Panel C. Direct support-configuration contrasts within each arch group.

Arch	Contrast	Outcome	Balanced estimate (95% CI)	Balanced p	Balanced q	Balanced dz	Full estimate (95% CI)	Full p	Full q	Full dz
Normal	12-24 - 9-24	MaxPP	-0.442 (-0.834 to -0.051)	0.030	0.139	-0.625	-0.190 (-0.500 to +0.120)	0.222	0.519	-0.196
Normal	12-24 - 9-24	MetaPP	-0.442 (-0.834 to -0.051)	0.030	0.139	-0.625	-0.191 (-0.504 to +0.122)	0.225	0.519	-0.195
Normal	12-24 - 9-24	MIF	+1.713 (+0.700 to +2.725)	0.003	0.041	+0.937	+1.209 (+0.577 to +1.841)	<0.001	0.006	+0.612
Normal	12-24 - 9-24	HIF	-0.067 (-1.226 to +1.092)	0.903	0.940	-0.032	-0.364 (-0.885 to +0.158)	0.167	0.485	-0.223

Arch	Contrast	Outcome	Balanced estimate (95% CI)	Balanced p	Balanced q	Balanced dz	Full estimate (95% CI)	Full p	Full q	Full dz
Normal	12-24 - 9-24	MLI	-1.874 (-4.096 to +0.348)	0.092	0.226	-0.467	-2.065 (-3.476 to -0.654)	0.005	0.039	-0.468
Normal	15-24 - 9-24	MaxPP	-0.462 (-0.884 to -0.040)	0.034	0.139	-0.606	-0.063 (-0.364 to +0.238)	0.676	0.825	-0.067
Normal	15-24 - 9-24	MetaPP	-0.487 (-0.928 to -0.045)	0.033	0.139	-0.610	-0.071 (-0.379 to +0.237)	0.643	0.825	-0.074
Normal	15-24 - 9-24	MIF	+2.067 (+1.241 to +2.893)	<0.001	0.003	+1.386	+1.418 (+0.797 to +2.038)	<0.001	0.001	+0.731
Normal	15-24 - 9-24	HIF	+0.626 (-0.401 to +1.652)	0.212	0.398	+0.337	-0.253 (-0.822 to +0.316)	0.374	0.660	-0.142
Normal	15-24 - 9-24	MLI	-3.083 (-5.243 to -0.922)	0.008	0.085	-0.790	-2.329 (-3.767 to -0.891)	0.002	0.022	-0.518
Normal	15-24 - 12-24	MaxPP	-0.020 (-0.567 to +0.528)	0.940	0.940	-0.020	+0.127 (-0.228 to +0.482)	0.473	0.746	+0.115
Normal	15-24 - 12-24	MetaPP	-0.045 (-0.607 to +0.518)	0.868	0.940	-0.044	+0.120 (-0.239 to +0.479)	0.503	0.755	+0.107
Normal	15-24 - 12-24	MIF	+0.355 (-0.499 to +1.208)	0.388	0.582	+0.230	+0.208 (-0.286 to +0.703)	0.399	0.665	+0.135
Normal	15-24 - 12-24	HIF	+0.692 (-0.335 to +1.720)	0.170	0.356	+0.373	+0.110 (-0.441 to +0.662)	0.688	0.825	+0.064
Normal	15-24 - 12-24	MLI	-1.209 (-4.298 to +1.881)	0.416	0.594	-0.217	-0.264 (-1.718 to +1.191)	0.716	0.826	-0.058
High	12-24 - 9-24	MaxPP	-0.021 (-0.478 to +0.437)	0.923	0.940	-0.025	-0.021 (-0.478 to +0.437)	0.923	0.923	-0.025
High	12-24 - 9-24	MetaPP	-0.051 (-0.514 to +0.411)	0.815	0.940	-0.061	-0.051 (-0.514 to +0.411)	0.815	0.906	-0.061
High	12-24 - 9-24	MIF	+0.642 (-0.328 to +1.613)	0.178	0.356	+0.366	+0.642 (-0.328 to +1.613)	0.178	0.485	+0.366
High	12-24 - 9-24	HIF	+0.082 (-1.271 to +1.436)	0.898	0.940	+0.034	+0.082 (-1.271 to +1.436)	0.898	0.923	+0.034
High	12-24 - 9-24	MLI	-2.032 (-4.491 to +0.427)	0.098	0.226	-0.458	-2.032 (-4.491 to +0.427)	0.098	0.327	-0.458
High	15-24 - 9-24	MaxPP	-0.231 (-0.471 to +0.008)	0.057	0.171	-0.536	-0.231 (-0.471 to +0.008)	0.057	0.244	-0.536
High	15-24 - 9-24	MetaPP	-0.257 (-0.496 to -0.018)	0.037	0.139	-0.594	-0.257 (-0.496 to -0.018)	0.037	0.223	-0.594
High	15-24 - 9-24	MIF	+0.972 (+0.003 to +1.942)	0.049	0.165	+0.556	+0.972 (+0.003 to +1.942)	0.049	0.244	+0.556
High	15-24 - 9-24	HIF	+0.437 (-1.024 to +1.898)	0.531	0.725	+0.166	+0.437 (-1.024 to +1.898)	0.531	0.759	+0.166
High	15-24 - 9-24	MLI	-2.149 (-4.468 to +0.169)	0.067	0.182	-0.513	-2.149 (-4.468 to +0.169)	0.067	0.250	-0.513
High	15-24 - 12-24	MaxPP	-0.210 (-0.667 to +0.246)	0.339	0.573	-0.255	-0.210 (-0.667 to +0.246)	0.339	0.660	-0.255
High	15-24 - 12-24	MetaPP	-0.205 (-0.669 to +0.258)	0.358	0.573	-0.245	-0.205 (-0.669 to +0.258)	0.358	0.660	-0.245
High	15-24 - 12-24	MIF	+0.330 (-0.422 to +1.083)	0.363	0.573	+0.243	+0.330 (-0.422 to +1.083)	0.363	0.660	+0.243
High	15-24 - 12-24	HIF	+0.355 (-0.961 to +1.671)	0.572	0.747	+0.149	+0.355 (-0.961 to +1.671)	0.572	0.781	+0.149
High	15-24 - 12-24	MLI	-0.118 (-1.692 to +1.456)	0.875	0.940	-0.041	-0.118 (-1.692 to +1.456)	0.875	0.923	-0.041

Panel D. Height-specific between-arch participant-level change comparisons (High Δ − Normal Δ).

Condition	Outcome	Balanced estimate (95% CI)	Balanced p	Balanced q	Balanced g	Full estimate (95% CI)	Full p	Full q	Full g
9-24	MaxPP	-0.279 (-1.486 to +0.929)	0.640	0.934	-0.168	-0.294 (-1.331 to +0.743)	0.562	0.902	-0.196
9-24	MetaPP	-0.083 (-1.289 to +1.123)	0.889	0.934	-0.050	-0.101 (-1.143 to +0.941)	0.842	0.902	-0.067
9-24	MIF	+1.524 (-1.422 to +4.470)	0.298	0.934	+0.378	+1.502 (-1.059 to +4.064)	0.234	0.902	+0.450

Condition	Outcome	Balanced estimate (95% CI)	Balanced p	Balanced q	Balanced g	Full estimate (95% CI)	Full p	Full q	Full g
9-24	HIF	-0.866 (-3.221 to +1.489)	0.454	0.934	-0.271	-1.256 (-3.454 to +0.942)	0.245	0.902	-0.464
9-24	MLI	+0.886 (-2.594 to +4.366)	0.606	0.934	+0.185	+0.343 (-2.808 to +3.494)	0.827	0.902	+0.055
12-24	MaxPP	+0.143 (-1.184 to +1.469)	0.826	0.934	+0.079	-0.125 (-1.356 to +1.106)	0.833	0.902	-0.081
12-24	MetaPP	+0.308 (-0.984 to +1.600)	0.626	0.934	+0.175	+0.038 (-1.160 to +1.237)	0.947	0.947	+0.025
12-24	MIF	+0.453 (-2.487 to +3.394)	0.754	0.934	+0.113	+0.935 (-1.623 to +3.494)	0.453	0.902	+0.280
12-24	HIF	-0.717 (-3.359 to +1.926)	0.582	0.934	-0.198	-0.810 (-3.114 to +1.494)	0.470	0.902	-0.267
12-24	MLI	+0.728 (-3.968 to +5.424)	0.753	0.934	+0.113	+0.376 (-3.465 to +4.218)	0.842	0.902	+0.060
15-24	MaxPP	-0.048 (-1.227 to +1.131)	0.934	0.934	-0.030	-0.463 (-1.456 to +0.530)	0.345	0.902	-0.302
15-24	MetaPP	+0.147 (-1.041 to +1.335)	0.801	0.934	+0.090	-0.287 (-1.296 to +0.722)	0.562	0.902	-0.186
15-24	MIF	+0.429 (-2.244 to +3.102)	0.744	0.934	+0.117	+1.057 (-1.243 to +3.358)	0.347	0.902	+0.360
15-24	HIF	-1.054 (-4.037 to +1.928)	0.471	0.934	-0.261	-0.566 (-3.350 to +2.218)	0.673	0.902	-0.172
15-24	MLI	+1.819 (-2.714 to +6.352)	0.418	0.934	+0.292	+0.522 (-3.324 to +4.369)	0.782	0.902	+0.083

Panel E. Family-level consistency summary.

Analysis family	Number of comparisons	Same effect direction	Same FDR conclusion	Maximum absolute estimate change
Single baseline	5	5/5	5/5	2.062
Support vs Single	30	30/30	24/30	1.297
Configuration	30	27/30	28/30	0.945
Between-arch Δ	15	13/15	15/15	1.297

Note. All p values are two-sided. q values are BH-FDR-adjusted within the same prespecified family separately for each sample frame. Hedges' g is reported for independent between-arch comparisons; Cohen's dz is reported for paired within-participant comparisons. The increase in FDR-supported within-normal-arch comparisons after expansion to n=40 should not be interpreted as an Arch Type × Condition effect. The directly relevant between-arch Δ family remained 0/15 FDR-supported.

Supplementary Table S23. Robustness checks for the full-cohort prespecified discrete sensitivity analyses.

Panel A. Agreement of unadjusted parametric and rank-based significance status.

Analysis family	Number of comparisons	Same unadjusted significance status	Different status
baseline	5	5	0
configs	30	29	1
delta	15	15	0
support	30	29	1

Panel B. Leave-one-participant-out sensitivity of the 15 between-arch Δ comparisons.

Condition	Outcome	Recomputations	Minimum p	Minimum q	FDR-significant recomputations	High Δ − Normal Δ range
9-24	MaxPP	55	0.410	0.778	0	-0.410～+0.131
9-24	MetaPP	55	0.251	0.782	0	-0.235～+0.321
9-24	MIF	55	0.165	0.778	0	+0.433～+1.760
9-24	HIF	55	0.178	0.778	0	-1.517～-0.661
9-24	MLI	55	0.599	0.782	0	-0.293～+0.816

Condition	Outcome	Recomputations	Minimum p	Minimum q	FDR-significant recomputations	High Δ − Normal Δ range
12-24	MaxPP	55	0.266	0.782	0	-0.285～+0.370
12-24	MetaPP	55	0.090	0.854	0	-0.110～+0.529
12-24	MIF	55	0.308	0.778	0	-0.098～+1.301
12-24	HIF	55	0.313	0.778	0	-1.154～-0.203
12-24	MLI	55	0.582	0.782	0	-0.276～+1.026
15-24	MaxPP	55	0.234	0.778	0	-0.575～-0.078
15-24	MetaPP	55	0.390	0.778	0	-0.432～+0.094
15-24	MIF	55	0.241	0.778	0	+0.083～+1.300
15-24	HIF	55	0.461	0.782	0	-1.004～+0.143
15-24	MLI	55	0.486	0.782	0	-0.291～+1.272

Panel C. Repeated Normal15 sampling from the full Normal40 cohort (10,000 draws; High15 fixed).

Condition	Outcome	Replicates	FDR-significant frequency	Mean estimate	2.5%–97.5% estimate range	Minimum q	Median q
9-24	MaxPP	10000	0.00%	-0.294	-0.824～+0.259	0.239	0.910
9-24	MetaPP	10000	0.00%	-0.101	-0.632～+0.447	0.251	0.923
9-24	MIF	10000	0.00%	+1.496	+0.370～+2.577	0.163	0.869
9-24	HIF	10000	0.00%	-1.259	-2.093～-0.445	0.175	0.878
9-24	MLI	10000	0.00%	+0.324	-2.254～+3.052	0.274	0.919
12-24	MaxPP	10000	0.00%	-0.126	-0.585～+0.375	0.366	0.927
12-24	MetaPP	10000	0.00%	+0.037	-0.421～+0.539	0.371	0.935
12-24	MIF	10000	0.00%	+0.932	-0.207～+2.010	0.204	0.886
12-24	HIF	10000	0.00%	-0.810	-1.816～+0.205	0.175	0.901
12-24	MLI	10000	0.00%	+0.366	-2.147～+2.815	0.327	0.923
15-24	MaxPP	10000	0.00%	-0.464	-1.022～+0.107	0.152	0.899
15-24	MetaPP	10000	0.00%	-0.288	-0.850～+0.282	0.157	0.906
15-24	MIF	10000	0.00%	+1.058	+0.101～+1.947	0.204	0.873
15-24	HIF	10000	0.00%	-0.563	-1.492～+0.356	0.422	0.920
15-24	MLI	10000	0.00%	+0.506	-1.916～+3.047	0.344	0.921

Note. Rank-based tests were robustness checks and did not replace the prespecified parametric analyses. In the between-arch Δ leave-one-participant-out analysis, no recomputation generated an FDR-supported comparison. In the Normal15 resampling analysis, the FDR-significant frequency was 0.00% for all 15 outcome-by-height comparisons.

Supplementary Table S24. Complete omnibus mixed-effects model comparisons and balanced-analysis replication check.

Frame	Outcome	N	Observations	LL full	LL reduced	χ^2 LRT	df	p	q (BH-5)	AIC full	BIC full	AIC reduced	BIC reduced
Balanced 15:15	MaxPP	30	120	-173.446	-173.888	0.884	3	0.829	0.835	366.893	394.768	361.777	381.289
Balanced 15:15	MetaPP	30	120	-171.677	-172.107	0.859	3	0.835	0.835	363.354	391.229	358.213	377.726
Balanced 15:15	MIF	30	120	-282.719	-283.889	2.339	3	0.505	0.835	585.438	613.313	581.777	601.290
Balanced 15:15	HIF	30	120	-291.815	-292.396	1.162	3	0.762	0.835	603.631	631.506	598.793	618.305
Balanced 15:15	MLI	30	120	-359.188	-359.718	1.060	3	0.787	0.835	738.376	766.251	733.436	752.948
Full 40:15	MaxPP	55	220	-335.089	-335.962	1.746	3	0.627	0.990	690.177	724.114	685.924	709.679
Full 40:15	MetaPP	55	220	-335.255	-335.708	0.907	3	0.824	0.990	690.510	724.446	685.417	709.172
Full 40:15	MIF	55	220	-513.976	-516.005	4.059	3	0.255	0.990	1047.952	1081.888	1046.011	1069.766

Frame	Outcome	N	Observations	LL full	LL reduced	χ² LRT	df	p	q (BH-5)	AIC full	BIC full	AIC reduced	BIC reduced
Full 40:15	HIF	55	220	-516.086	-517.544	2.918	3	0.405	0.990	1052.171	1086.108	1049.089	1072.844
Full 40:15	MLI	55	220	-681.159	-681.217	0.116	3	0.990	0.990	1382.319	1416.255	1376.435	1400.190

Panel B. Reproduction of the five balanced 15:15 manuscript omnibus tests.

Outcome	Manuscript χ²	Recomputed χ²	Absolute χ² difference	Manuscript p	Recomputed p	Absolute p difference	Matches 3 decimals
MaxPP	0.884	0.883968	0.000032	0.829	0.829295	0.000295	True
MetaPP	0.859	0.859080	0.000080	0.835	0.835290	0.000290	True
MIF	2.339	2.339037	0.000037	0.505	0.505084	0.000084	True
HIF	1.162	1.162259	0.000259	0.762	0.762070	0.000070	True
MLI	1.060	1.060160	0.000160	0.787	0.786699	0.000301	True

Note. Omnibus Arch Type × Condition interactions were evaluated by 3-df likelihood-ratio comparison of the full and reduced models. BH-FDR was applied across the five outcomes separately within each sample frame. All five balanced results reproduced the manuscript to the reported three-decimal precision.

Supplementary Table S25. Complete fixed-effect coefficients from the balanced and full-cohort mixed-effects models.

Frame	Outcome	Term	Estimate (95% Wald CI)	SE	Wald z	Unadjusted p
Balanced 15:15	MaxPP	Intercept	+8.483 (+7.918 to +9.047) N/cm²	0.288	29.448	<0.001
Balanced 15:15	MaxPP	ArchHigh	-0.107 (-0.905 to +0.692) N/cm²	0.407	-0.262	0.794
Balanced 15:15	MaxPP	Condition[9-24]	-0.278 (-0.908 to +0.352) N/cm²	0.321	-0.865	0.387
Balanced 15:15	MaxPP	Condition[12-24]	-0.720 (-1.350 to -0.090) N/cm²	0.321	-2.240	0.025
Balanced 15:15	MaxPP	Condition[15-24]	-0.740 (-1.370 to -0.110) N/cm²	0.321	-2.301	0.021
Balanced 15:15	MaxPP	ArchHigh×Condition[9-24]	-0.279 (-1.170 to +0.612) N/cm²	0.455	-0.613	0.540
Balanced 15:15	MaxPP	ArchHigh×Condition[12-24]	+0.143 (-0.749 to +1.034) N/cm²	0.455	0.314	0.754
Balanced 15:15	MaxPP	ArchHigh×Condition[15-24]	-0.048 (-0.939 to +0.843) N/cm²	0.455	-0.106	0.915
Balanced 15:15	MetaPP	Intercept	+8.477 (+7.928 to +9.026) N/cm²	0.280	30.254	<0.001
Balanced 15:15	MetaPP	ArchHigh	-0.311 (-1.088 to +0.465) N/cm²	0.396	-0.786	0.432
Balanced 15:15	MetaPP	Condition[9-24]	-0.272 (-0.901 to +0.356) N/cm²	0.321	-0.850	0.396
Balanced 15:15	MetaPP	Condition[12-24]	-0.714 (-1.343 to -0.086) N/cm²	0.321	-2.229	0.026
Balanced 15:15	MetaPP	Condition[15-24]	-0.759 (-1.387 to -0.131) N/cm²	0.321	-2.368	0.018
Balanced 15:15	MetaPP	ArchHigh×Condition[9-24]	-0.083 (-0.971 to +0.806) N/cm²	0.453	-0.183	0.855
Balanced 15:15	MetaPP	ArchHigh×Condition[12-24]	+0.308 (-0.581 to +1.197) N/cm²	0.453	0.679	0.497
Balanced 15:15	MetaPP	ArchHigh×Condition[15-24]	+0.147 (-0.741 to +1.036) N/cm²	0.453	0.325	0.745
Balanced 15:15	MIF	Intercept	+20.998 (+19.396 to +22.601) pp	0.818	25.676	<0.001
Balanced 15:15	MIF	ArchHigh	-1.246 (-3.513 to +1.021) pp	1.157	-1.078	0.281
Balanced 15:15	MIF	Condition[9-24]	+2.037 (+0.606 to +3.469) pp	0.730	2.789	0.005
Balanced 15:15	MIF	Condition[12-24]	+3.750 (+2.318 to +5.182) pp	0.730	5.134	<0.001

Frame	Outcome	Term	Estimate (95% Wald CI)	SE	Wald z	Unadjusted p
Balanced 15:15	MIF	Condition[15-24]	+4.105 (+2.673 to +5.536) pp	0.730	5.620	<0.001
Balanced 15:15	MIF	ArchHigh×Condition[9-24]	+1.524 (-0.501 to +3.548) pp	1.033	1.475	0.140
Balanced 15:15	MIF	ArchHigh×Condition[12-24]	+0.453 (-1.571 to +2.478) pp	1.033	0.439	0.661
Balanced 15:15	MIF	ArchHigh×Condition[15-24]	+0.429 (-1.596 to +2.453) pp	1.033	0.415	0.678
Balanced 15:15	HIF	Intercept	+12.188 (+10.215 to +14.162) pp	1.007	12.105	<0.001
Balanced 15:15	HIF	ArchHigh	+4.140 (+1.349 to +6.931) pp	1.424	2.908	0.004
Balanced 15:15	HIF	Condition[9-24]	-1.753 (-3.199 to -0.307) pp	0.738	-2.376	0.017
Balanced 15:15	HIF	Condition[12-24]	-1.820 (-3.266 to -0.374) pp	0.738	-2.467	0.014
Balanced 15:15	HIF	Condition[15-24]	-1.128 (-2.574 to +0.318) pp	0.738	-1.528	0.126
Balanced 15:15	HIF	ArchHigh×Condition[9-24]	-0.866 (-2.911 to +1.179) pp	1.043	-0.830	0.407
Balanced 15:15	HIF	ArchHigh×Condition[12-24]	-0.717 (-2.761 to +1.328) pp	1.043	-0.687	0.492
Balanced 15:15	HIF	ArchHigh×Condition[15-24]	-1.054 (-3.099 to +0.991) pp	1.043	-1.011	0.312
Balanced 15:15	MLI	Intercept	+30.536 (+26.825 to +34.247) pp	1.893	16.129	<0.001
Balanced 15:15	MLI	ArchHigh	-2.449 (-7.696 to +2.799) pp	2.677	-0.915	0.360
Balanced 15:15	MLI	Condition[9-24]	-4.260 (-6.718 to -1.802) pp	1.254	-3.397	<0.001
Balanced 15:15	MLI	Condition[12-24]	-6.134 (-8.592 to -3.676) pp	1.254	-4.891	<0.001
Balanced 15:15	MLI	Condition[15-24]	-7.343 (-9.801 to -4.885) pp	1.254	-5.855	<0.001
Balanced 15:15	MLI	ArchHigh×Condition[9-24]	+0.886 (-2.590 to +4.362) pp	1.774	0.499	0.617
Balanced 15:15	MLI	ArchHigh×Condition[12-24]	+0.728 (-2.748 to +4.204) pp	1.774	0.410	0.681
Balanced 15:15	MLI	ArchHigh×Condition[15-24]	+1.819 (-1.657 to +5.295) pp	1.774	1.026	0.305
Full 40:15	MaxPP	Intercept	+8.556 (+8.130 to +8.983) N/cm²	0.218	39.321	<0.001
Full 40:15	MaxPP	ArchHigh	-0.180 (-0.997 to +0.636) N/cm²	0.417	-0.433	0.665
Full 40:15	MaxPP	Condition[9-24]	-0.263 (-0.644 to +0.119) N/cm²	0.195	-1.350	0.177
Full 40:15	MaxPP	Condition[12-24]	-0.453 (-0.834 to -0.071) N/cm²	0.195	-2.327	0.020
Full 40:15	MaxPP	Condition[15-24]	-0.325 (-0.707 to +0.056) N/cm²	0.195	-1.673	0.094
Full 40:15	MaxPP	ArchHigh×Condition[9-24]	-0.294 (-1.024 to +0.436) N/cm²	0.373	-0.789	0.430
Full 40:15	MaxPP	ArchHigh×Condition[12-24]	-0.125 (-0.855 to +0.605) N/cm²	0.373	-0.336	0.737
Full 40:15	MaxPP	ArchHigh×Condition[15-24]	-0.463 (-1.193 to +0.267) N/cm²	0.373	-1.242	0.214
Full 40:15	MetaPP	Intercept	+8.537 (+8.111 to +8.962) N/cm²	0.217	39.304	<0.001
Full 40:15	MetaPP	ArchHigh	-0.371 (-1.186 to +0.444) N/cm²	0.416	-0.892	0.372

Frame	Outcome	Term	Estimate (95% Wald CI)	SE	Wald z	Unadjusted p
Full 40:15	MetaPP	Condition[9-24]	-0.254 (-0.636 to +0.128) N/cm²	0.195	-1.302	0.193
Full 40:15	MetaPP	Condition[12-24]	-0.445 (-0.827 to -0.063) N/cm²	0.195	-2.281	0.023
Full 40:15	MetaPP	Condition[15-24]	-0.325 (-0.707 to +0.057) N/cm²	0.195	-1.666	0.096
Full 40:15	MetaPP	ArchHigh×Condition[9-24]	-0.101 (-0.833 to +0.631) N/cm²	0.373	-0.271	0.786
Full 40:15	MetaPP	ArchHigh×Condition[12-24]	+0.038 (-0.694 to +0.770) N/cm²	0.373	0.102	0.918
Full 40:15	MetaPP	ArchHigh×Condition[15-24]	-0.287 (-1.019 to +0.445) N/cm²	0.373	-0.768	0.442
Full 40:15	MIF	Intercept	+21.331 (+20.156 to +22.507) pp	0.600	35.556	<0.001
Full 40:15	MIF	ArchHigh	-1.579 (-3.831 to +0.672) pp	1.149	-1.375	0.169
Full 40:15	MIF	Condition[9-24]	+2.059 (+1.278 to +2.840) pp	0.398	5.167	<0.001
Full 40:15	MIF	Condition[12-24]	+3.268 (+2.487 to +4.049) pp	0.398	8.201	<0.001
Full 40:15	MIF	Condition[15-24]	+3.476 (+2.695 to +4.257) pp	0.398	8.725	<0.001
Full 40:15	MIF	ArchHigh×Condition[9-24]	+1.502 (+0.007 to +2.998) pp	0.763	1.969	0.049
Full 40:15	MIF	ArchHigh×Condition[12-24]	+0.935 (-0.560 to +2.431) pp	0.763	1.226	0.220
Full 40:15	MIF	ArchHigh×Condition[15-24]	+1.057 (-0.438 to +2.553) pp	0.763	1.386	0.166
Full 40:15	HIF	Intercept	+12.080 (+10.804 to +13.356) pp	0.651	18.555	<0.001
Full 40:15	HIF	ArchHigh	+4.248 (+1.805 to +6.692) pp	1.247	3.408	<0.001
Full 40:15	HIF	Condition[9-24]	-1.363 (-2.128 to -0.598) pp	0.390	-3.491	<0.001
Full 40:15	HIF	Condition[12-24]	-1.726 (-2.491 to -0.961) pp	0.390	-4.422	<0.001
Full 40:15	HIF	Condition[15-24]	-1.616 (-2.381 to -0.851) pp	0.390	-4.139	<0.001
Full 40:15	HIF	ArchHigh×Condition[9-24]	-1.256 (-2.721 to +0.209) pp	0.748	-1.680	0.093
Full 40:15	HIF	ArchHigh×Condition[12-24]	-0.810 (-2.275 to +0.655) pp	0.748	-1.084	0.278
Full 40:15	HIF	ArchHigh×Condition[15-24]	-0.566 (-2.031 to +0.899) pp	0.748	-0.757	0.449
Full 40:15	MLI	Intercept	+28.474 (+25.807 to +31.141) pp	1.361	20.927	<0.001
Full 40:15	MLI	ArchHigh	-0.387 (-5.493 to +4.720) pp	2.605	-0.148	0.882
Full 40:15	MLI	Condition[9-24]	-3.718 (-5.347 to -2.089) pp	0.831	-4.473	<0.001
Full 40:15	MLI	Condition[12-24]	-5.783 (-7.412 to -4.154) pp	0.831	-6.957	<0.001
Full 40:15	MLI	Condition[15-24]	-6.046 (-7.676 to -4.417) pp	0.831	-7.275	<0.001
Full 40:15	MLI	ArchHigh×Condition[9-24]	+0.343 (-2.776 to +3.463) pp	1.592	0.216	0.829
Full 40:15	MLI	ArchHigh×Condition[12-24]	+0.376 (-2.743 to +3.496) pp	1.592	0.236	0.813
Full 40:15	MLI	ArchHigh×Condition[15-24]	+0.522 (-2.597 to +3.642) pp	1.592	0.328	0.743

Note. Normal arch and Single are the reference levels. Confidence intervals are nominal asymptotic Wald intervals. These coefficients are reported as complete model output; multiplicity-controlled inference concerning effect modification is based on the five omnibus interaction tests rather than individual fixed-effect p values.

Supplementary Table S26. Complete model-based contrasts from the balanced and full-cohort mixed-effects models.

Panel A. Model-based baseline contrasts.

Frame	Outcome	Contrast	Estimate (95% Wald CI)	SE	Wald z	Unadjusted p	Descriptive q†
Balanced 15:15	MaxPP	High - Normal at Single	-0.107 (-0.905 to +0.692) N/cm²	0.407	-0.262	0.794	
Balanced 15:15	MetaPP	High - Normal at Single	-0.311 (-1.088 to +0.465) N/cm²	0.396	-0.786	0.432	
Balanced 15:15	MIF	High - Normal at Single	-1.246 (-3.513 to +1.021) pp	1.157	-1.078	0.281	
Balanced 15:15	HIF	High - Normal at Single	+4.140 (+1.349 to +6.931) pp	1.424	2.908	0.004	
Balanced 15:15	MLI	High - Normal at Single	-2.449 (-7.696 to +2.799) pp	2.677	-0.915	0.360	
Full 40:15	MaxPP	High - Normal at Single	-0.180 (-0.997 to +0.636) N/cm²	0.417	-0.433	0.665	
Full 40:15	MetaPP	High - Normal at Single	-0.371 (-1.186 to +0.444) N/cm²	0.416	-0.892	0.372	
Full 40:15	MIF	High - Normal at Single	-1.579 (-3.831 to +0.672) pp	1.149	-1.375	0.169	
Full 40:15	HIF	High - Normal at Single	+4.248 (+1.805 to +6.692) pp	1.247	3.408	<0.001	
Full 40:15	MLI	High - Normal at Single	-0.387 (-5.493 to +4.720) pp	2.605	-0.148	0.882	

Panel B. Model-based support-versus-Single contrasts.

Frame	Outcome	Contrast	Estimate (95% Wald CI)	SE	Wald z	Unadjusted p	Descriptive q†
Balanced 15:15	MaxPP	Normal: 9-24 - Single	-0.278 (-0.908 to +0.352) N/cm²	0.321	-0.865	0.387	
Balanced 15:15	MaxPP	High: 9-24 - Single	-0.557 (-1.187 to +0.073) N/cm²	0.321	-1.732	0.083	
Balanced 15:15	MaxPP	Normal: 12-24 - Single	-0.720 (-1.350 to -0.090) N/cm²	0.321	-2.240	0.025	
Balanced 15:15	MaxPP	High: 12-24 - Single	-0.578 (-1.208 to +0.052) N/cm²	0.321	-1.797	0.072	
Balanced 15:15	MaxPP	Normal: 15-24 - Single	-0.740 (-1.370 to -0.110) N/cm²	0.321	-2.301	0.021	
Balanced 15:15	MaxPP	High: 15-24 - Single	-0.788 (-1.418 to -0.158) N/cm²	0.321	-2.451	0.014	
Balanced 15:15	MetaPP	Normal: 9-24 - Single	-0.272 (-0.901 to +0.356) N/cm²	0.321	-0.850	0.396	
Balanced 15:15	MetaPP	High: 9-24 - Single	-0.355 (-0.983 to +0.273) N/cm²	0.321	-1.108	0.268	
Balanced 15:15	MetaPP	Normal: 12-24 - Single	-0.714 (-1.343 to -0.086) N/cm²	0.321	-2.229	0.026	
Balanced 15:15	MetaPP	High: 12-24 - Single	-0.406 (-1.035 to +0.222) N/cm²	0.321	-1.268	0.205	
Balanced 15:15	MetaPP	Normal: 15-24 - Single	-0.759 (-1.387 to -0.131) N/cm²	0.321	-2.368	0.018	
Balanced 15:15	MetaPP	High: 15-24 - Single	-0.612 (-1.240 to +0.017) N/cm²	0.321	-1.908	0.056	
Balanced 15:15	MIF	Normal: 9-24 - Single	+2.037 (+0.606 to +3.469) pp	0.730	2.789	0.005	
Balanced 15:15	MIF	High: 9-24 - Single	+3.561 (+2.129 to +4.993) pp	0.730	4.875	<0.001	
Balanced 15:15	MIF	Normal: 12-24 - Single	+3.750 (+2.318 to +5.182) pp	0.730	5.134	<0.001	
Balanced 15:15	MIF	High: 12-24 - Single	+4.203 (+2.772 to +5.635) pp	0.730	5.755	<0.001	

Frame	Outcome	Contrast	Estimate (95% Wald CI)	SE	Wald z	Unadjusted p	Descriptive q†
Balanced 15:15	MIF	Normal: 15-24 - Single	+4.105 (+2.673 to +5.536) pp	0.730	5.620	<0.001	
Balanced 15:15	MIF	High: 15-24 - Single	+4.533 (+3.102 to +5.965) pp	0.730	6.207	<0.001	
Balanced 15:15	HIF	Normal: 9-24 - Single	-1.753 (-3.199 to -0.307) pp	0.738	-2.376	0.017	
Balanced 15:15	HIF	High: 9-24 - Single	-2.619 (-4.065 to -1.173) pp	0.738	-3.550	<0.001	
Balanced 15:15	HIF	Normal: 12-24 - Single	-1.820 (-3.266 to -0.374) pp	0.738	-2.467	0.014	
Balanced 15:15	HIF	High: 12-24 - Single	-2.537 (-3.983 to -1.091) pp	0.738	-3.438	<0.001	
Balanced 15:15	HIF	Normal: 15-24 - Single	-1.128 (-2.574 to +0.318) pp	0.738	-1.528	0.126	
Balanced 15:15	HIF	High: 15-24 - Single	-2.182 (-3.628 to -0.736) pp	0.738	-2.958	0.003	
Balanced 15:15	MLI	Normal: 9-24 - Single	-4.260 (-6.718 to -1.802) pp	1.254	-3.397	<0.001	
Balanced 15:15	MLI	High: 9-24 - Single	-3.375 (-5.833 to -0.917) pp	1.254	-2.691	0.007	
Balanced 15:15	MLI	Normal: 12-24 - Single	-6.134 (-8.592 to -3.676) pp	1.254	-4.891	<0.001	
Balanced 15:15	MLI	High: 12-24 - Single	-5.406 (-7.864 to -2.948) pp	1.254	-4.311	<0.001	
Balanced 15:15	MLI	Normal: 15-24 - Single	-7.343 (-9.801 to -4.885) pp	1.254	-5.855	<0.001	
Balanced 15:15	MLI	High: 15-24 - Single	-5.524 (-7.982 to -3.066) pp	1.254	-4.405	<0.001	
Full 40:15	MaxPP	Normal: 9-24 - Single	-0.263 (-0.644 to +0.119) N/cm²	0.195	-1.350	0.177	
Full 40:15	MaxPP	High: 9-24 - Single	-0.557 (-1.179 to +0.066) N/cm²	0.318	-1.753	0.080	
Full 40:15	MaxPP	Normal: 12-24 - Single	-0.453 (-0.834 to -0.071) N/cm²	0.195	-2.327	0.020	
Full 40:15	MaxPP	High: 12-24 - Single	-0.578 (-1.200 to +0.045) N/cm²	0.318	-1.818	0.069	
Full 40:15	MaxPP	Normal: 15-24 - Single	-0.325 (-0.707 to +0.056) N/cm²	0.195	-1.673	0.094	
Full 40:15	MaxPP	High: 15-24 - Single	-0.788 (-1.411 to -0.165) N/cm²	0.318	-2.481	0.013	
Full 40:15	MetaPP	Normal: 9-24 - Single	-0.254 (-0.636 to +0.128) N/cm²	0.195	-1.302	0.193	
Full 40:15	MetaPP	High: 9-24 - Single	-0.355 (-0.979 to +0.269) N/cm²	0.318	-1.115	0.265	
Full 40:15	MetaPP	Normal: 12-24 - Single	-0.445 (-0.827 to -0.063) N/cm²	0.195	-2.281	0.023	
Full 40:15	MetaPP	High: 12-24 - Single	-0.406 (-1.031 to +0.218) N/cm²	0.318	-1.276	0.202	
Full 40:15	MetaPP	Normal: 15-24 - Single	-0.325 (-0.707 to +0.057) N/cm²	0.195	-1.666	0.096	
Full 40:15	MetaPP	High: 15-24 - Single	-0.612 (-1.236 to +0.012) N/cm²	0.318	-1.921	0.055	
Full 40:15	MIF	Normal: 9-24 - Single	+2.059 (+1.278 to +2.840) pp	0.398	5.167	<0.001	
Full 40:15	MIF	High: 9-24 - Single	+3.561 (+2.286 to +4.836) pp	0.651	5.473	<0.001	
Full 40:15	MIF	Normal: 12-24 - Single	+3.268 (+2.487 to +4.049) pp	0.398	8.201	<0.001	
Full 40:15	MIF	High: 12-24 - Single	+4.203 (+2.928 to +5.479) pp	0.651	6.460	<0.001	
Full 40:15	MIF	Normal: 15-24 - Single	+3.476 (+2.695 to +4.257) pp	0.398	8.725	<0.001	
Full 40:15	MIF	High: 15-24 - Single	+4.533 (+3.258 to +5.809) pp	0.651	6.968	<0.001	

Frame	Outcome	Contrast	Estimate (95% Wald CI)	SE	Wald z	Unadjusted p	Descriptive q†
Full 40:15	HIF	Normal: 9-24 - Single	-1.363 (-2.128 to -0.598) pp	0.390	-3.491	<0.001	
Full 40:15	HIF	High: 9-24 - Single	-2.619 (-3.868 to -1.370) pp	0.637	-4.108	<0.001	
Full 40:15	HIF	Normal: 12-24 - Single	-1.726 (-2.491 to -0.961) pp	0.390	-4.422	<0.001	
Full 40:15	HIF	High: 12-24 - Single	-2.537 (-3.786 to -1.287) pp	0.637	-3.979	<0.001	
Full 40:15	HIF	Normal: 15-24 - Single	-1.616 (-2.381 to -0.851) pp	0.390	-4.139	<0.001	
Full 40:15	HIF	High: 15-24 - Single	-2.182 (-3.431 to -0.932) pp	0.637	-3.423	<0.001	
Full 40:15	MLI	Normal: 9-24 - Single	-3.718 (-5.347 to -2.089) pp	0.831	-4.473	<0.001	
Full 40:15	MLI	High: 9-24 - Single	-3.375 (-6.035 to -0.714) pp	1.357	-2.486	0.013	
Full 40:15	MLI	Normal: 12-24 - Single	-5.783 (-7.412 to -4.154) pp	0.831	-6.957	<0.001	
Full 40:15	MLI	High: 12-24 - Single	-5.406 (-8.067 to -2.746) pp	1.357	-3.983	<0.001	
Full 40:15	MLI	Normal: 15-24 - Single	-6.046 (-7.676 to -4.417) pp	0.831	-7.275	<0.001	
Full 40:15	MLI	High: 15-24 - Single	-5.524 (-8.184 to -2.864) pp	1.357	-4.070	<0.001	

Panel C. Model-based Arch Type × Condition interaction contrasts (High Δ − Normal Δ).

Frame	Outcome	Contrast	Estimate (95% Wald CI)	SE	Wald z	Unadjusted p	Descriptive q†
Balanced 15:15	MaxPP	High Δ - Normal Δ at 9-24	-0.279 (-1.170 to +0.612) N/cm²	0.455	-0.613	0.540	0.870
Balanced 15:15	MaxPP	High Δ - Normal Δ at 12-24	+0.143 (-0.749 to +1.034) N/cm²	0.455	0.314	0.754	0.870
Balanced 15:15	MaxPP	High Δ - Normal Δ at 15-24	-0.048 (-0.939 to +0.843) N/cm²	0.455	-0.106	0.915	0.915
Balanced 15:15	MetaPP	High Δ - Normal Δ at 9-24	-0.083 (-0.971 to +0.806) N/cm²	0.453	-0.183	0.855	0.915
Balanced 15:15	MetaPP	High Δ - Normal Δ at 12-24	+0.308 (-0.581 to +1.197) N/cm²	0.453	0.679	0.497	0.870
Balanced 15:15	MetaPP	High Δ - Normal Δ at 15-24	+0.147 (-0.741 to +1.036) N/cm²	0.453	0.325	0.745	0.870
Balanced 15:15	MIF	High Δ - Normal Δ at 9-24	+1.524 (-0.501 to +3.548) pp	1.033	1.475	0.140	0.870
Balanced 15:15	MIF	High Δ - Normal Δ at 12-24	+0.453 (-1.571 to +2.478) pp	1.033	0.439	0.661	0.870
Balanced 15:15	MIF	High Δ - Normal Δ at 15-24	+0.429 (-1.596 to +2.453) pp	1.033	0.415	0.678	0.870
Balanced 15:15	HIF	High Δ - Normal Δ at 9-24	-0.866 (-2.911 to +1.179) pp	1.043	-0.830	0.407	0.870
Balanced 15:15	HIF	High Δ - Normal Δ at 12-24	-0.717 (-2.761 to +1.328) pp	1.043	-0.687	0.492	0.870
Balanced 15:15	HIF	High Δ - Normal Δ at 15-24	-1.054 (-3.099 to +0.991) pp	1.043	-1.011	0.312	0.870
Balanced 15:15	MLI	High Δ - Normal Δ at 9-24	+0.886 (-2.590 to +4.362) pp	1.774	0.499	0.617	0.870
Balanced 15:15	MLI	High Δ - Normal Δ at 12-24	+0.728 (-2.748 to +4.204) pp	1.774	0.410	0.681	0.870
Balanced 15:15	MLI	High Δ - Normal Δ at 15-24	+1.819 (-1.657 to +5.295) pp	1.774	1.026	0.305	0.870
Full 40:15	MaxPP	High Δ - Normal Δ at 9-24	-0.294 (-1.024 to +0.436) N/cm²	0.373	-0.789	0.430	0.748
Full 40:15	MaxPP	High Δ - Normal Δ at 12-24	-0.125 (-0.855 to +0.605) N/cm²	0.373	-0.336	0.737	0.889
Full 40:15	MaxPP	High Δ - Normal Δ at 15-24	-0.463 (-1.193 to +0.267) N/cm²	0.373	-1.242	0.214	0.660

Frame	Outcome	Contrast	Estimate (95% Wald CI)	SE	Wald z	Unadjusted p	Descriptive q†
Full 40:15	MetaPP	High Δ - Normal Δ at 9-24	-0.101 (-0.833 to +0.631) N/cm²	0.373	-0.271	0.786	0.889
Full 40:15	MetaPP	High Δ - Normal Δ at 12-24	+0.038 (-0.694 to +0.770) N/cm²	0.373	0.102	0.918	0.918
Full 40:15	MetaPP	High Δ - Normal Δ at 15-24	-0.287 (-1.019 to +0.445) N/cm²	0.373	-0.768	0.442	0.748
Full 40:15	MIF	High Δ - Normal Δ at 9-24	+1.502 (+0.007 to +2.998) pp	0.763	1.969	0.049	0.660
Full 40:15	MIF	High Δ - Normal Δ at 12-24	+0.935 (-0.560 to +2.431) pp	0.763	1.226	0.220	0.660
Full 40:15	MIF	High Δ - Normal Δ at 15-24	+1.057 (-0.438 to +2.553) pp	0.763	1.386	0.166	0.660
Full 40:15	HIF	High Δ - Normal Δ at 9-24	-1.256 (-2.721 to +0.209) pp	0.748	-1.680	0.093	0.660
Full 40:15	HIF	High Δ - Normal Δ at 12-24	-0.810 (-2.275 to +0.655) pp	0.748	-1.084	0.278	0.696
Full 40:15	HIF	High Δ - Normal Δ at 15-24	-0.566 (-2.031 to +0.899) pp	0.748	-0.757	0.449	0.748
Full 40:15	MLI	High Δ - Normal Δ at 9-24	+0.343 (-2.776 to +3.463) pp	1.592	0.216	0.829	0.889
Full 40:15	MLI	High Δ - Normal Δ at 12-24	+0.376 (-2.743 to +3.496) pp	1.592	0.236	0.813	0.889
Full 40:15	MLI	High Δ - Normal Δ at 15-24	+0.522 (-2.597 to +3.642) pp	1.592	0.328	0.743	0.889

Panel D. Model-based direct configuration contrasts.

Frame	Outcome	Contrast	Estimate (95% Wald CI)	SE	Wald z	Unadjusted p	Descriptive q†
Balanced 15:15	MaxPP	Normal: 12-24 - 9-24	-0.442 (-1.072 to +0.188) N/cm²	0.321	-1.375	0.169	
Balanced 15:15	MaxPP	High: 12-24 - 9-24	-0.021 (-0.651 to +0.609) N/cm²	0.321	-0.065	0.948	
Balanced 15:15	MaxPP	Normal: 15-24 - 9-24	-0.462 (-1.092 to +0.168) N/cm²	0.321	-1.436	0.151	
Balanced 15:15	MaxPP	High: 15-24 - 9-24	-0.231 (-0.861 to +0.399) N/cm²	0.321	-0.720	0.472	
Balanced 15:15	MaxPP	Normal: 15-24 - 12-24	-0.020 (-0.650 to +0.611) N/cm²	0.321	-0.061	0.951	
Balanced 15:15	MaxPP	High: 15-24 - 12-24	-0.210 (-0.841 to +0.420) N/cm²	0.321	-0.655	0.513	
Balanced 15:15	MetaPP	Normal: 12-24 - 9-24	-0.442 (-1.070 to +0.186) N/cm²	0.321	-1.379	0.168	
Balanced 15:15	MetaPP	High: 12-24 - 9-24	-0.051 (-0.680 to +0.577) N/cm²	0.321	-0.160	0.873	
Balanced 15:15	MetaPP	Normal: 15-24 - 9-24	-0.487 (-1.115 to +0.142) N/cm²	0.321	-1.518	0.129	
Balanced 15:15	MetaPP	High: 15-24 - 9-24	-0.257 (-0.885 to +0.372) N/cm²	0.321	-0.801	0.423	
Balanced 15:15	MetaPP	Normal: 15-24 - 12-24	-0.045 (-0.673 to +0.584) N/cm²	0.321	-0.139	0.889	
Balanced 15:15	MetaPP	High: 15-24 - 12-24	-0.205 (-0.834 to +0.423) N/cm²	0.321	-0.641	0.522	
Balanced 15:15	MIF	Normal: 12-24 - 9-24	+1.713 (+0.281 to +3.144) pp	0.730	2.345	0.019	
Balanced 15:15	MIF	High: 12-24 - 9-24	+0.642 (-0.789 to +2.074) pp	0.730	0.879	0.379	
Balanced 15:15	MIF	Normal: 15-24 - 9-24	+2.067 (+0.636 to +3.499) pp	0.730	2.830	0.005	
Balanced 15:15	MIF	High: 15-24 - 9-24	+0.972 (-0.459 to +2.404) pp	0.730	1.331	0.183	
Balanced 15:15	MIF	Normal: 15-24 - 12-24	+0.355 (-1.077 to +1.786) pp	0.730	0.486	0.627	
Balanced 15:15	MIF	High: 15-24 - 12-24	+0.330 (-1.101 to +1.762) pp	0.730	0.452	0.651	

Frame	Outcome	Contrast	Estimate (95% Wald CI)	SE	Wald z	Unadjusted p	Descriptive q†
Balanced 15:15	HIF	Normal: 12-24 - 9-24	-0.067 (-1.513 to +1.379) pp	0.738	-0.091	0.928	
Balanced 15:15	HIF	High: 12-24 - 9-24	+0.082 (-1.364 to +1.528) pp	0.738	0.112	0.911	
Balanced 15:15	HIF	Normal: 15-24 - 9-24	+0.626 (-0.820 to +2.071) pp	0.738	0.848	0.397	
Balanced 15:15	HIF	High: 15-24 - 9-24	+0.437 (-1.009 to +1.883) pp	0.738	0.592	0.554	
Balanced 15:15	HIF	Normal: 15-24 - 12-24	+0.692 (-0.753 to +2.138) pp	0.738	0.939	0.348	
Balanced 15:15	HIF	High: 15-24 - 12-24	+0.355 (-1.091 to +1.801) pp	0.738	0.481	0.631	
Balanced 15:15	MLI	Normal: 12-24 - 9-24	-1.874 (-4.332 to +0.584) pp	1.254	-1.494	0.135	
Balanced 15:15	MLI	High: 12-24 - 9-24	-2.032 (-4.490 to +0.426) pp	1.254	-1.620	0.105	
Balanced 15:15	MLI	Normal: 15-24 - 9-24	-3.083 (-5.541 to -0.625) pp	1.254	-2.458	0.014	
Balanced 15:15	MLI	High: 15-24 - 9-24	-2.149 (-4.607 to +0.309) pp	1.254	-1.714	0.087	
Balanced 15:15	MLI	Normal: 15-24 - 12-24	-1.209 (-3.667 to +1.249) pp	1.254	-0.964	0.335	
Balanced 15:15	MLI	High: 15-24 - 12-24	-0.118 (-2.576 to +2.340) pp	1.254	-0.094	0.925	
Full 40:15	MaxPP	Normal: 12-24 - 9-24	-0.190 (-0.571 to +0.191) N/cm²	0.195	-0.977	0.329	
Full 40:15	MaxPP	High: 12-24 - 9-24	-0.021 (-0.644 to +0.602) N/cm²	0.318	-0.066	0.948	
Full 40:15	MaxPP	Normal: 15-24 - 9-24	-0.063 (-0.444 to +0.319) N/cm²	0.195	-0.323	0.747	
Full 40:15	MaxPP	High: 15-24 - 9-24	-0.231 (-0.854 to +0.391) N/cm²	0.318	-0.728	0.467	
Full 40:15	MaxPP	Normal: 15-24 - 12-24	+0.127 (-0.254 to +0.509) N/cm²	0.195	0.654	0.513	
Full 40:15	MaxPP	High: 15-24 - 12-24	-0.210 (-0.833 to +0.412) N/cm²	0.318	-0.662	0.508	
Full 40:15	MetaPP	Normal: 12-24 - 9-24	-0.191 (-0.573 to +0.191) N/cm²	0.195	-0.979	0.328	
Full 40:15	MetaPP	High: 12-24 - 9-24	-0.051 (-0.675 to +0.573) N/cm²	0.318	-0.161	0.872	
Full 40:15	MetaPP	Normal: 15-24 - 9-24	-0.071 (-0.453 to +0.311) N/cm²	0.195	-0.364	0.716	
Full 40:15	MetaPP	High: 15-24 - 9-24	-0.257 (-0.881 to +0.367) N/cm²	0.318	-0.806	0.420	
Full 40:15	MetaPP	Normal: 15-24 - 12-24	+0.120 (-0.262 to +0.502) N/cm²	0.195	0.615	0.539	
Full 40:15	MetaPP	High: 15-24 - 12-24	-0.205 (-0.829 to +0.419) N/cm²	0.318	-0.645	0.519	
Full 40:15	MIF	Normal: 12-24 - 9-24	+1.209 (+0.428 to +1.990) pp	0.398	3.034	0.002	
Full 40:15	MIF	High: 12-24 - 9-24	+0.642 (-0.633 to +1.917) pp	0.651	0.987	0.324	
Full 40:15	MIF	Normal: 15-24 - 9-24	+1.418 (+0.637 to +2.198) pp	0.398	3.558	<0.001	
Full 40:15	MIF	High: 15-24 - 9-24	+0.972 (-0.303 to +2.248) pp	0.651	1.495	0.135	
Full 40:15	MIF	Normal: 15-24 - 12-24	+0.208 (-0.572 to +0.989) pp	0.398	0.523	0.601	
Full 40:15	MIF	High: 15-24 - 12-24	+0.330 (-0.945 to +1.605) pp	0.651	0.507	0.612	
Full 40:15	HIF	Normal: 12-24 - 9-24	-0.364 (-1.129 to +0.402) pp	0.390	-0.931	0.352	
Full 40:15	HIF	High: 12-24 - 9-24	+0.082 (-1.167 to +1.332) pp	0.637	0.129	0.897	

Frame	Outcome	Contrast	Estimate (95% Wald CI)	SE	Wald z	Unadjusted p	Descriptive q†
Full 40:15	HIF	Normal: 15-24 - 9-24	-0.253 (-1.018 to +0.512) pp	0.390	-0.648	0.517	
Full 40:15	HIF	High: 15-24 - 9-24	+0.437 (-0.812 to +1.687) pp	0.637	0.686	0.493	
Full 40:15	HIF	Normal: 15-24 - 12-24	+0.110 (-0.655 to +0.876) pp	0.390	0.283	0.777	
Full 40:15	HIF	High: 15-24 - 12-24	+0.355 (-0.895 to +1.604) pp	0.637	0.556	0.578	
Full 40:15	MLI	Normal: 12-24 - 9-24	-2.065 (-3.694 to -0.436) pp	0.831	-2.484	0.013	
Full 40:15	MLI	High: 12-24 - 9-24	-2.032 (-4.692 to +0.629) pp	1.357	-1.497	0.134	
Full 40:15	MLI	Normal: 15-24 - 9-24	-2.329 (-3.958 to -0.700) pp	0.831	-2.802	0.005	
Full 40:15	MLI	High: 15-24 - 9-24	-2.149 (-4.810 to +0.511) pp	1.357	-1.584	0.113	
Full 40:15	MLI	Normal: 15-24 - 12-24	-0.264 (-1.893 to +1.365) pp	0.831	-0.317	0.751	
Full 40:15	MLI	High: 15-24 - 12-24	-0.118 (-2.778 to +2.543) pp	1.357	-0.087	0.931	

Note. All contrasts are derived from the fitted LMMs. The descriptive BH q values are defined only for the 15 individual interaction coefficients within each sample frame; blank cells indicate that no BH-15 adjustment was assigned to that contrast family. These model-based baseline, support, and configuration contrasts are provided as complete model output and do not replace the corresponding prespecified paired/Welch comparisons.

Supplementary Table S27. Mixed-effects model variance components, residual diagnostics, and influence analyses.

Panel A. Model convergence and variance components.

Frame	Outcome	Full converged	Reduced converged	Random-intercept variance	Residual variance	ICC
Balanced 15:15	MaxPP	True	True	0.470	0.775	0.377
Balanced 15:15	MetaPP	True	True	0.407	0.771	0.345
Balanced 15:15	MIF	True	True	6.032	4.001	0.601
Balanced 15:15	HIF	True	True	11.125	4.082	0.732
Balanced 15:15	MLI	True	True	41.968	11.796	0.781
Full 40:15	MaxPP	True	True	1.137	0.757	0.600
Full 40:15	MetaPP	True	True	1.127	0.760	0.597
Full 40:15	MIF	True	True	11.222	3.175	0.779
Full 40:15	HIF	True	True	13.906	3.048	0.820
Full 40:15	MLI	True	True	60.234	13.817	0.813

Panel B. Conditional-residual and random-effect diagnostics.

Frame	Outcome	Residual Shapiro p	Skewness	Excess kurtosis	Max |std residual|	N |std residual| >3	Max-residual ID	Arch	Condition	Spearman ρ |resid| vs fitted	Spearman p	Random-effect Shapiro p
Balanced 15:15	MaxPP	<0.001	2.852	16.040	5.829	2	H07	High	Single	0.148	0.106	0.226
Balanced 15:15	MetaPP	<0.001	2.874	16.049	5.875	2	H07	High	Single	0.079	0.393	0.752
Balanced 15:15	MIF	<0.001	-2.533	15.312	5.721	2	H07	High	Single	-0.208	0.022	0.847
Balanced 15:15	HIF	<0.001	0.711	2.069	3.322	2	H07	High	Single	0.084	0.363	0.001
Balanced 15:15	MLI	0.513	-0.210	0.468	2.579	0	N02	Normal	12-24	-0.046	0.615	0.735
Full 40:15	MaxPP	<0.001	1.650	8.342	5.565	2	H07	High	Single	0.322	<0.001	0.041
Full 40:15	MetaPP	<0.001	1.601	7.938	5.517	2	H07	High	Single	0.304	<0.001	0.086
Full 40:15	MIF	<0.001	-1.845	12.616	6.220	2	H07	High	Single	-0.094	0.165	0.003

Frame	Outcome	Residual Shapiro p	Skewness	Excess kurtosis	Max |std residual|	N |std residual| >3	Max-residual ID	Arch	Condition	Spearman ρ |resid| vs fitted	Spearman p	Random-effect Shapiro p
Full 40:15	HIF	<0.001	0.618	2.482	3.814	2	H07	High	Single	0.091	0.179	0.267
Full 40:15	MLI	0.002	0.401	2.208	4.269	1	N16	Normal	Single	-0.041	0.545	0.039

Panel C. Targeted omission of the participant with the largest absolute standardized residual in the full cohort.

Outcome	Omitted ID	Arch	Condition	Max |std residual|	Original omnibus p	Omit-one χ²	Omit-one p	Conclusion remained p≥0.05
MaxPP	H07	High	Single	5.565	0.627	2.053	0.562	True
MetaPP	H07	High	Single	5.517	0.824	2.965	0.397	True
MIF	H07	High	Single	6.220	0.255	0.751	0.861	True
HIF	H07	High	Single	3.814	0.405	1.419	0.701	True
MLI	N16	Normal	Single	4.269	0.990	0.054	0.997	True

Panel D. Complete full-cohort LMM leave-one-participant-out summary (55 omissions per outcome).

Outcome	Omissions	Minimum p	Maximum p	Median p	N p<0.05
MaxPP	55	0.412	0.774	0.617	0
MetaPP	55	0.397	0.911	0.821	0
MIF	55	0.099	0.861	0.260	0
HIF	55	0.246	0.714	0.403	0
MLI	55	0.884	0.999	0.988	0

Note. All full and reduced models converged and all random-intercept variances were positive. Residual diagnostics show departures from Gaussian normality for several outcomes; these departures were treated as limitations on model-based uncertainty estimates rather than as a reason to discard the full-cohort analysis. Neither targeted omission nor complete LMM leave-one-participant-out analysis produced a significant omnibus interaction.

Supplementary Table S28. Cross-check of model-based interaction contrasts against the prespecified Welch participant-level change-score comparisons.

Frame	Outcome	Condition	LMM estimate (95% Wald CI)	LMM p	Welch Δ estimate (95% CI)	Welch p	Welch q	Rank p
Balanced 15:15	MaxPP	9-24	-0.279 (-1.170 to +0.612)	0.540	-0.279 (-1.486 to +0.929)	0.640	0.934	0.340
Balanced 15:15	MaxPP	12-24	+0.143 (-0.749 to +1.034)	0.754	+0.143 (-1.184 to +1.469)	0.826	0.934	0.213
Balanced 15:15	MaxPP	15-24	-0.048 (-0.939 to +0.843)	0.915	-0.048 (-1.227 to +1.131)	0.934	0.934	0.340
Balanced 15:15	MetaPP	9-24	-0.083 (-0.971 to +0.806)	0.855	-0.083 (-1.289 to +1.123)	0.889	0.934	0.709
Balanced 15:15	MetaPP	12-24	+0.308 (-0.581 to +1.197)	0.497	+0.308 (-0.984 to +1.600)	0.626	0.934	0.089
Balanced 15:15	MetaPP	15-24	+0.147 (-0.741 to +1.036)	0.745	+0.147 (-1.041 to +1.335)	0.801	0.934	0.199
Balanced 15:15	MIF	9-24	+1.524 (-0.501 to +3.548)	0.140	+1.524 (-1.422 to +4.470)	0.298	0.934	0.199
Balanced 15:15	MIF	12-24	+0.453 (-1.571 to +2.478)	0.661	+0.453 (-2.487 to +3.394)	0.754	0.934	0.740
Balanced 15:15	MIF	15-24	+0.429 (-1.596 to +2.453)	0.678	+0.429 (-2.244 to +3.102)	0.744	0.934	0.934
Balanced 15:15	HIF	9-24	-0.866 (-2.911 to +1.179)	0.407	-0.866 (-3.221 to +1.489)	0.454	0.934	0.967
Balanced 15:15	HIF	12-24	-0.717 (-2.761 to +1.328)	0.492	-0.717 (-3.359 to +1.926)	0.582	0.934	0.803
Balanced 15:15	HIF	15-24	-1.054 (-3.099 to +0.991)	0.312	-1.054 (-4.037 to +1.928)	0.471	0.934	0.803
Balanced 15:15	MLI	9-24	+0.886 (-2.590 to +4.362)	0.617	+0.886 (-2.594 to +4.366)	0.606	0.934	0.590
Balanced 15:15	MLI	12-24	+0.728 (-2.748 to +4.204)	0.681	+0.728 (-3.968 to +5.424)	0.753	0.934	0.648

Frame	Outcome	Condition	LMM estimate (95% Wald CI)	LMM p	Welch Δ estimate (95% CI)	Welch p	Welch q	Rank p
Balanced 15:15	MLI	15-24	+1.819 (-1.657 to +5.295)	0.305	+1.819 (-2.714 to +6.352)	0.418	0.934	0.384
Full 40:15	MaxPP	9-24	-0.294 (-1.024 to +0.436)	0.430	-0.294 (-1.331 to +0.743)	0.562	0.902	0.902
Full 40:15	MaxPP	12-24	-0.125 (-0.855 to +0.605)	0.737	-0.125 (-1.356 to +1.106)	0.833	0.902	0.411
Full 40:15	MaxPP	15-24	-0.463 (-1.193 to +0.267)	0.214	-0.463 (-1.456 to +0.530)	0.345	0.902	0.917
Full 40:15	MetaPP	9-24	-0.101 (-0.833 to +0.631)	0.786	-0.101 (-1.143 to +0.941)	0.842	0.902	0.755
Full 40:15	MetaPP	12-24	+0.038 (-0.694 to +0.770)	0.918	+0.038 (-1.160 to +1.237)	0.947	0.947	0.195
Full 40:15	MetaPP	15-24	-0.287 (-1.019 to +0.445)	0.442	-0.287 (-1.296 to +0.722)	0.562	0.902	0.784
Full 40:15	MIF	9-24	+1.502 (+0.007 to +2.998)	0.049	+1.502 (-1.059 to +4.064)	0.234	0.902	0.269
Full 40:15	MIF	12-24	+0.935 (-0.560 to +2.431)	0.220	+0.935 (-1.623 to +3.494)	0.453	0.902	0.564
Full 40:15	MIF	15-24	+1.057 (-0.438 to +2.553)	0.166	+1.057 (-1.243 to +3.358)	0.347	0.902	0.577
Full 40:15	HIF	9-24	-1.256 (-2.721 to +0.209)	0.093	-1.256 (-3.454 to +0.942)	0.245	0.902	0.630
Full 40:15	HIF	12-24	-0.810 (-2.275 to +0.655)	0.278	-0.810 (-3.114 to +1.494)	0.470	0.902	0.755
Full 40:15	HIF	15-24	-0.566 (-2.031 to +0.899)	0.449	-0.566 (-3.350 to +2.218)	0.673	0.902	0.872
Full 40:15	MLI	9-24	+0.343 (-2.776 to +3.463)	0.829	+0.343 (-2.808 to +3.494)	0.827	0.902	0.887
Full 40:15	MLI	12-24	+0.376 (-2.743 to +3.496)	0.813	+0.376 (-3.465 to +4.218)	0.842	0.902	0.828
Full 40:15	MLI	15-24	+0.522 (-2.597 to +3.642)	0.743	+0.522 (-3.324 to +4.369)	0.782	0.902	0.977

Note. The LMM and Welch analyses target the same High Δ − Normal Δ point contrast but use different uncertainty models. The MIF 9-24 full-cohort point estimate is identical (+1.502 pp) in both analyses, while the LMM Wald interval is narrower and narrowly excludes zero; the corresponding MIF omnibus interaction, multiplicity-adjusted individual interaction coefficient, and prespecified Welch Δ comparison do not support a robust arch-specific response. Accordingly, the isolated unadjusted LMM coefficient was not interpreted as establishing effect modification.

Supplementary Table S29. Additional sampling-frame and outlier diagnostics for the full-cohort sensitivity analysis.

Panel A. Original 15 normal-arch participants versus the additional 25 eligible normal-arch participants.

Type	Condition	Outcome	Original15	Additional25	Additional25 − Original15	95% CI	Welch p	Exploratory q	Hedges g	Rank p
Condition level	Single	MaxPP	8.483	8.601	+0.118	-0.899 to 1.135	0.814	0.943	+0.076	0.801
Condition level	Single	MetaPP	8.477	8.572	+0.096	-0.920 to 1.111	0.849	0.943	+0.061	0.801
Condition level	Single	MIF	20.998	21.531	+0.533	-2.272 to 3.337	0.702	0.943	+0.119	0.911
Condition level	Single	HIF	12.188	12.015	-0.173	-2.844 to 2.498	0.896	0.944	-0.038	0.801
Condition level	Single	MLI	30.536	27.237	-3.299	-9.195 to 2.596	0.264	0.943	-0.337	0.451
Condition level	9-24	MaxPP	8.204	8.347	+0.143	-0.629 to 0.915	0.710	0.943	+0.100	0.615
Condition level	9-24	MetaPP	8.204	8.330	+0.125	-0.651 to 0.901	0.745	0.943	+0.088	0.557
Condition level	9-24	MIF	23.036	23.603	+0.567	-1.876 to 3.010	0.641	0.943	+0.132	0.911
Condition level	9-24	HIF	10.435	10.887	+0.451	-1.978 to 2.881	0.709	0.943	+0.110	0.823
Condition level	9-24	MLI	26.276	23.845	-2.431	-7.827 to 2.966	0.368	0.943	-0.262	0.485
Condition level	12-24	MaxPP	7.762	8.309	+0.546	-0.272 to 1.364	0.184	0.943	+0.369	0.557
Condition level	12-24	MetaPP	7.762	8.290	+0.527	-0.298 to 1.353	0.204	0.943	+0.352	0.576
Condition level	12-24	MIF	24.748	24.510	-0.239	-2.602 to 2.124	0.839	0.943	-0.061	0.716
Condition level	12-24	HIF	10.368	10.345	-0.023	-2.571 to 2.526	0.985	0.985	-0.006	0.911
Condition level	12-24	MLI	24.402	21.665	-2.737	-8.642 to 3.169	0.353	0.943	-0.287	0.576

Type	Condition	Outcome	Original15	Additional25	Additional25 − Original15	95% CI	Welch p	Exploratory q	Hedges g	Rank p
Condition level	15-24	MaxPP	7.743	8.524	+0.781	-0.009 to 1.571	0.052	0.537	+0.545	0.171
Condition level	15-24	MetaPP	7.718	8.508	+0.790	-0.013 to 1.594	0.054	0.537	+0.543	0.139
Condition level	15-24	MIF	25.103	24.630	-0.473	-2.656 to 1.710	0.664	0.943	-0.122	0.503
Condition level	15-24	HIF	11.061	10.106	-0.954	-3.197 to 1.289	0.394	0.943	-0.249	0.451
Condition level	15-24	MLI	23.193	21.968	-1.225	-5.892 to 3.443	0.598	0.943	-0.150	1.000
Support response Δ	9-24	MaxPP	-0.278	-0.253	+0.025	-0.934 to 0.983	0.958	0.973	+0.017	0.434
Support response Δ	9-24	MetaPP	-0.272	-0.243	+0.030	-0.922 to 0.981	0.950	0.973	+0.021	0.468
Support response Δ	9-24	MIF	2.037	2.072	+0.034	-2.033 to 2.101	0.973	0.973	+0.012	0.576
Support response Δ	9-24	HIF	-1.753	-1.129	+0.624	-0.801 to 2.050	0.378	0.708	+0.290	0.199
Support response Δ	9-24	MLI	-4.260	-3.392	+0.868	-3.144 to 4.881	0.664	0.973	+0.126	0.485
Support response Δ	12-24	MaxPP	-0.720	-0.292	+0.428	-0.395 to 1.251	0.296	0.708	+0.346	0.557
Support response Δ	12-24	MetaPP	-0.714	-0.283	+0.432	-0.387 to 1.250	0.290	0.708	+0.348	0.434
Support response Δ	12-24	MIF	3.750	2.978	-0.772	-2.826 to 1.283	0.445	0.741	-0.271	0.675
Support response Δ	12-24	HIF	-1.820	-1.670	+0.150	-1.723 to 2.023	0.870	0.973	+0.057	0.867
Support response Δ	12-24	MLI	-6.134	-5.572	+0.563	-3.679 to 4.804	0.788	0.973	+0.087	0.557
Support response Δ	15-24	MaxPP	-0.740	-0.077	+0.663	-0.325 to 1.651	0.180	0.708	+0.450	0.099
Support response Δ	15-24	MetaPP	-0.759	-0.064	+0.695	-0.288 to 1.677	0.159	0.708	+0.473	0.088
Support response Δ	15-24	MIF	4.105	3.099	-1.005	-2.835 to 0.824	0.265	0.708	-0.417	0.386
Support response Δ	15-24	HIF	-1.128	-1.909	-0.781	-2.459 to 0.896	0.347	0.708	-0.322	0.402
Support response Δ	15-24	MLI	-7.343	-5.269	+2.074	-2.025 to 6.174	0.310	0.708	+0.324	0.162

Note. Condition-level rows compare the two normal-arch subsets at each observed condition. Support-response Δ rows compare participant-level changes from Single. These comparisons were diagnostic checks of the balanced normal-arch sampling frame, not new primary hypothesis tests. The q values shown here are exploratory. The absence of FDR-supported subset differences does not demonstrate that the two subsets are statistically equivalent.

Panel B. Outlier-flag audit summary in the full-cohort sensitivity data.

Analysis level	Flag records	Participants flagged	IQR flags	Modified-Z flags	Participants with IQR flag	Participants with modified-Z flag
Participant-level Δ	45	10	45	23	10	5
Participant × Condition	51	20	48	26	19	12

Note. IQR and modified-Z criteria were used only to flag observations for audit. Flagged observations were not automatically excluded from the sensitivity analyses. Influence was evaluated separately through leave-one-participant-out analyses.

Supplementary Table S30. Complete participant-level leave-one-out results for the full-cohort mixed-effects omnibus interaction tests.

Outcome	Omitted participant	Arch	LRT $\chi^2(3)$	p
MaxPP	H01	High	1.417	0.702
MaxPP	H02	High	1.863	0.601
MaxPP	H03	High	1.825	0.609
MaxPP	H04	High	2.215	0.529
MaxPP	H05	High	1.242	0.743
MaxPP	H06	High	1.841	0.606
MaxPP	H07	High	2.053	0.562
MaxPP	H08	High	1.983	0.576

Outcome	Omitted participant	Arch	LRT $\chi^2(3)$	p
MaxPP	H09	High	2.043	0.563
MaxPP	H10	High	2.016	0.569
MaxPP	H11	High	1.909	0.592
MaxPP	H12	High	2.284	0.516
MaxPP	H13	High	1.601	0.659
MaxPP	H14	High	1.573	0.665
MaxPP	H15	High	2.092	0.554
MaxPP	N01	Normal	1.646	0.649
MaxPP	N02	Normal	1.955	0.582
MaxPP	N03	Normal	1.283	0.733
MaxPP	N04	Normal	1.751	0.626
MaxPP	N05	Normal	1.802	0.614
MaxPP	N06	Normal	2.044	0.563
MaxPP	N07	Normal	1.707	0.635
MaxPP	N08	Normal	1.833	0.608
MaxPP	N09	Normal	1.673	0.643
MaxPP	N10	Normal	1.748	0.626
MaxPP	N11	Normal	1.813	0.612
MaxPP	N12	Normal	1.791	0.617
MaxPP	N13	Normal	1.403	0.705
MaxPP	N14	Normal	1.853	0.603
MaxPP	N15	Normal	2.874	0.412
MaxPP	N16	Normal	1.588	0.662
MaxPP	N17	Normal	1.775	0.620
MaxPP	N18	Normal	1.729	0.630
MaxPP	N19	Normal	1.113	0.774
MaxPP	N20	Normal	1.901	0.593
MaxPP	N21	Normal	1.709	0.635
MaxPP	N22	Normal	2.053	0.562
MaxPP	N23	Normal	1.791	0.617
MaxPP	N24	Normal	1.765	0.623
MaxPP	N25	Normal	1.620	0.655
MaxPP	N26	Normal	1.753	0.625
MaxPP	N27	Normal	1.790	0.617
MaxPP	N28	Normal	1.698	0.637
MaxPP	N29	Normal	1.365	0.714
MaxPP	N30	Normal	1.818	0.611
MaxPP	N31	Normal	1.858	0.602
MaxPP	N32	Normal	1.713	0.634
MaxPP	N33	Normal	2.438	0.487
MaxPP	N34	Normal	1.631	0.652
MaxPP	N35	Normal	1.882	0.597
MaxPP	N36	Normal	1.491	0.684
MaxPP	N37	Normal	1.663	0.645
MaxPP	N38	Normal	1.334	0.721
MaxPP	N39	Normal	1.857	0.603
MaxPP	N40	Normal	1.624	0.654
MetaPP	H01	High	0.583	0.900
MetaPP	H02	High	0.759	0.859
MetaPP	H03	High	0.775	0.856
MetaPP	H04	High	1.269	0.736
MetaPP	H05	High	0.646	0.886

Outcome	Omitted participant	Arch	LRT $\chi^2(3)$	p
MetaPP	H06	High	0.878	0.831
MetaPP	H07	High	2.965	0.397
MetaPP	H08	High	0.837	0.841
MetaPP	H09	High	1.162	0.762
MetaPP	H10	High	0.984	0.805
MetaPP	H11	High	0.854	0.837
MetaPP	H12	High	1.187	0.756
MetaPP	H13	High	0.743	0.863
MetaPP	H14	High	1.578	0.664
MetaPP	H15	High	1.091	0.779
MetaPP	N01	Normal	0.883	0.829
MetaPP	N02	Normal	1.022	0.796
MetaPP	N03	Normal	0.576	0.902
MetaPP	N04	Normal	0.913	0.822
MetaPP	N05	Normal	1.073	0.784
MetaPP	N06	Normal	1.150	0.765
MetaPP	N07	Normal	0.921	0.820
MetaPP	N08	Normal	1.016	0.797
MetaPP	N09	Normal	0.901	0.825
MetaPP	N10	Normal	0.944	0.815
MetaPP	N11	Normal	0.916	0.821
MetaPP	N12	Normal	0.965	0.810
MetaPP	N13	Normal	0.689	0.876
MetaPP	N14	Normal	0.975	0.807
MetaPP	N15	Normal	1.449	0.694
MetaPP	N16	Normal	0.698	0.874
MetaPP	N17	Normal	0.885	0.829
MetaPP	N18	Normal	0.918	0.821
MetaPP	N19	Normal	0.536	0.911
MetaPP	N20	Normal	1.043	0.791
MetaPP	N21	Normal	0.908	0.824
MetaPP	N22	Normal	1.044	0.791
MetaPP	N23	Normal	0.965	0.810
MetaPP	N24	Normal	0.968	0.809
MetaPP	N25	Normal	0.850	0.838
MetaPP	N26	Normal	0.923	0.820
MetaPP	N27	Normal	0.910	0.823
MetaPP	N28	Normal	0.781	0.854
MetaPP	N29	Normal	0.625	0.891
MetaPP	N30	Normal	1.045	0.790
MetaPP	N31	Normal	0.980	0.806
MetaPP	N32	Normal	0.878	0.831
MetaPP	N33	Normal	1.422	0.700
MetaPP	N34	Normal	0.845	0.839
MetaPP	N35	Normal	0.933	0.818
MetaPP	N36	Normal	0.754	0.860
MetaPP	N37	Normal	0.954	0.812
MetaPP	N38	Normal	0.774	0.856
MetaPP	N39	Normal	0.918	0.821
MetaPP	N40	Normal	0.875	0.832
MIF	H01	High	4.266	0.234
MIF	H02	High	3.389	0.335

Outcome	Omitted participant	Arch	LRT $\chi^2(3)$	p
MIF	H03	High	4.436	0.218
MIF	H04	High	3.913	0.271
MIF	H05	High	3.373	0.338
MIF	H06	High	4.365	0.225
MIF	H07	High	0.751	0.861
MIF	H08	High	5.113	0.164
MIF	H09	High	4.172	0.243
MIF	H10	High	4.661	0.198
MIF	H11	High	4.935	0.177
MIF	H12	High	4.481	0.214
MIF	H13	High	5.249	0.154
MIF	H14	High	3.848	0.278
MIF	H15	High	4.184	0.242
MIF	N01	Normal	3.805	0.283
MIF	N02	Normal	3.311	0.346
MIF	N03	Normal	3.780	0.286
MIF	N04	Normal	4.348	0.226
MIF	N05	Normal	4.045	0.257
MIF	N06	Normal	3.998	0.262
MIF	N07	Normal	3.890	0.274
MIF	N08	Normal	3.770	0.287
MIF	N09	Normal	3.760	0.289
MIF	N10	Normal	3.903	0.272
MIF	N11	Normal	4.338	0.227
MIF	N12	Normal	3.912	0.271
MIF	N13	Normal	3.919	0.270
MIF	N14	Normal	4.337	0.227
MIF	N15	Normal	6.271	0.099
MIF	N16	Normal	3.906	0.272
MIF	N17	Normal	3.971	0.265
MIF	N18	Normal	3.948	0.267
MIF	N19	Normal	4.131	0.248
MIF	N20	Normal	3.960	0.266
MIF	N21	Normal	4.096	0.251
MIF	N22	Normal	3.506	0.320
MIF	N23	Normal	3.900	0.272
MIF	N24	Normal	3.541	0.315
MIF	N25	Normal	4.015	0.260
MIF	N26	Normal	4.141	0.247
MIF	N27	Normal	4.337	0.227
MIF	N28	Normal	4.561	0.207
MIF	N29	Normal	4.742	0.192
MIF	N30	Normal	3.748	0.290
MIF	N31	Normal	4.047	0.256
MIF	N32	Normal	3.696	0.296
MIF	N33	Normal	4.086	0.252
MIF	N34	Normal	4.146	0.246
MIF	N35	Normal	4.309	0.230
MIF	N36	Normal	4.296	0.231
MIF	N37	Normal	3.658	0.301
MIF	N38	Normal	3.685	0.298
MIF	N39	Normal	4.203	0.240

Outcome	Omitted participant	Arch	LRT $\chi^2(3)$	p
MIF	N40	Normal	3.552	0.314
HIF	H01	High	3.994	0.262
HIF	H02	High	3.800	0.284
HIF	H03	High	2.987	0.394
HIF	H04	High	3.192	0.363
HIF	H05	High	2.602	0.457
HIF	H06	High	3.687	0.297
HIF	H07	High	1.419	0.701
HIF	H08	High	2.657	0.448
HIF	H09	High	4.146	0.246
HIF	H10	High	3.346	0.341
HIF	H11	High	3.533	0.317
HIF	H12	High	2.422	0.489
HIF	H13	High	4.049	0.256
HIF	H14	High	1.366	0.714
HIF	H15	High	3.247	0.355
HIF	N01	Normal	3.173	0.366
HIF	N02	Normal	2.701	0.440
HIF	N03	Normal	3.074	0.380
HIF	N04	Normal	2.612	0.455
HIF	N05	Normal	3.068	0.381
HIF	N06	Normal	3.171	0.366
HIF	N07	Normal	2.753	0.431
HIF	N08	Normal	3.237	0.357
HIF	N09	Normal	3.038	0.386
HIF	N10	Normal	2.924	0.403
HIF	N11	Normal	2.550	0.466
HIF	N12	Normal	3.117	0.374
HIF	N13	Normal	2.954	0.399
HIF	N14	Normal	2.577	0.462
HIF	N15	Normal	3.481	0.323
HIF	N16	Normal	2.557	0.465
HIF	N17	Normal	2.732	0.435
HIF	N18	Normal	2.975	0.396
HIF	N19	Normal	2.874	0.411
HIF	N20	Normal	2.829	0.419
HIF	N21	Normal	2.923	0.404
HIF	N22	Normal	2.661	0.447
HIF	N23	Normal	3.171	0.366
HIF	N24	Normal	3.332	0.343
HIF	N25	Normal	2.906	0.406
HIF	N26	Normal	2.880	0.411
HIF	N27	Normal	2.682	0.443
HIF	N28	Normal	2.752	0.431
HIF	N29	Normal	2.819	0.420
HIF	N30	Normal	2.969	0.396
HIF	N31	Normal	2.938	0.401
HIF	N32	Normal	2.878	0.411
HIF	N33	Normal	2.520	0.472
HIF	N34	Normal	2.735	0.434
HIF	N35	Normal	2.504	0.475
HIF	N36	Normal	2.878	0.411

Outcome	Omitted participant	Arch	LRT $\chi^2(3)$	p
HIF	N37	Normal	3.279	0.351
HIF	N38	Normal	2.996	0.392
HIF	N39	Normal	2.350	0.503
HIF	N40	Normal	3.234	0.357
MLI	H01	High	0.212	0.976
MLI	H02	High	0.025	0.999
MLI	H03	High	0.043	0.998
MLI	H04	High	0.450	0.930
MLI	H05	High	0.458	0.928
MLI	H06	High	0.125	0.989
MLI	H07	High	0.031	0.999
MLI	H08	High	0.498	0.919
MLI	H09	High	0.088	0.993
MLI	H10	High	0.127	0.988
MLI	H11	High	0.562	0.905
MLI	H12	High	0.051	0.997
MLI	H13	High	0.171	0.982
MLI	H14	High	0.653	0.884
MLI	H15	High	0.042	0.998
MLI	N01	Normal	0.138	0.987
MLI	N02	Normal	0.233	0.972
MLI	N03	Normal	0.032	0.998
MLI	N04	Normal	0.146	0.986
MLI	N05	Normal	0.121	0.989
MLI	N06	Normal	0.070	0.995
MLI	N07	Normal	0.158	0.984
MLI	N08	Normal	0.081	0.994
MLI	N09	Normal	0.077	0.994
MLI	N10	Normal	0.074	0.995
MLI	N11	Normal	0.073	0.995
MLI	N12	Normal	0.135	0.987
MLI	N13	Normal	0.101	0.992
MLI	N14	Normal	0.090	0.993
MLI	N15	Normal	0.310	0.958
MLI	N16	Normal	0.054	0.997
MLI	N17	Normal	0.052	0.997
MLI	N18	Normal	0.171	0.982
MLI	N19	Normal	0.294	0.961
MLI	N20	Normal	0.097	0.992
MLI	N21	Normal	0.272	0.965
MLI	N22	Normal	0.129	0.988
MLI	N23	Normal	0.082	0.994
MLI	N24	Normal	0.188	0.980
MLI	N25	Normal	0.123	0.989
MLI	N26	Normal	0.199	0.978
MLI	N27	Normal	0.082	0.994
MLI	N28	Normal	0.134	0.987
MLI	N29	Normal	0.166	0.983
MLI	N30	Normal	0.206	0.977
MLI	N31	Normal	0.145	0.986
MLI	N32	Normal	0.220	0.974
MLI	N33	Normal	0.093	0.993

Outcome	Omitted participant	Arch	LRT χ²(3)	p
MLI	N34	Normal	0.257	0.968
MLI	N35	Normal	0.097	0.992
MLI	N36	Normal	0.153	0.985
MLI	N37	Normal	0.198	0.978
MLI	N38	Normal	0.100	0.992
MLI	N39	Normal	0.050	0.997
MLI	N40	Normal	0.137	0.987

Note. Each row represents a complete re-fit of the full and reduced mixed-effects models after omitting one participant. The reported p value is the 3-df likelihood-ratio test for Arch Type × Condition in that leave-one-out dataset. There were 55 omissions for each of the five outcomes (275 re-fits in total), and no leave-one-out omnibus test had $p < 0.05$. These analyses were diagnostic robustness checks and did not define an additional multiplicity-controlled primary hypothesis family.
